# Heterogeneous stochastic bifurcations explain intrinsic oscillatory patterns in entorhinal cortical stellate cells

**DOI:** 10.1073/pnas.2202962119

**Published:** 2022-12-19

**Authors:** Divyansh Mittal, Rishikesh Narayanan

**Affiliations:** a Cellular Neurophysiology Laboratory, Molecular Biophysics Unit, Indian Institute of Science, Bangalore 560012, India

**Keywords:** stochastic resonance, intrinsic oscillations, parametric variability, nonlinear dynamical system, biophysical models of stellate cells

## Abstract

Stellate cells (SC) in the medial entorhinal cortex manifest intrinsic membrane potential oscillatory patterns. Although different theoretical frameworks have been proposed to explain these patterns, a robust unifying framework that jointly accounts for intrinsic heterogeneities and stochasticity is missing. Here, we first performed in vitro patchclamp electrophysiological recordings from rat SCs and found pronounced cell-to-cell variability in their characteristic physiological properties, including peri-threshold oscillatory patterns. We demonstrate that noise introduced into two independent populations (endowed with deterministic or stochastic ion-channel gating kinetics) of heterogeneous biophysical models yielded activity patterns that were qualitatively similar to electrophysiological peri-threshold oscillatory activity in SCs. We developed spectrogram-based quantitative metrics for the identification of valid oscillations and confirmed that these metrics reliably captured the variable-amplitude and arhythmic oscillatory patterns observed in electrophysiological recordings. Using these quantitative metrics, we validated activity patterns from both heterogeneous populations of SC models, with each model assessed with multiple trials of different levels of noise at distinct membrane depolarizations. Our analyses unveiled the manifestation of stochastic resonance (detection of the highest number of valid oscillatory traces at an optimal level of noise) in both heterogeneous populations of SC models. Finally, we show that a generalized network motif comprised of a slow negative feedback loop amplified by a fast positive feedback loop manifested stochastic bifurcations and stochastic resonance in the emergence of oscillations. Together, through a unique convergence of the degeneracy and stochastic resonance frameworks, our unifying framework centered on heterogeneous stochastic bifurcations argues for state-dependent emergence of SC oscillations.

Neurons in the entorhinal cortex are positioned at a crucial stage of information processing. They receive polymodal sensory information and provide spatial information to the hippocampus (1, 2). One of the spatially selective cell types in the medial entorhinal cortex (MEC) is the layer II stellate cells (SC). A subset of SCs that elicit action potentials (APs) forming a hexagonal or triangular grid-like repetitive pattern tiling the spatial environment are called grid cells (1–4). A signature intrinsic property of SCs is the emergence of peri-threshold oscillations upon depolarization closer to the spiking threshold (5–11).

Ever since their discovery more than three decades ago (5), peri-threshold oscillatory patterns in SCs have been central to several studies debating their origins and implications (5–23). These activity patterns have been considered to be emergent from interactions between hyperpolarization-activated cyclic nucleotide-gated (HCN) and persistent sodium (NaP) channels (9, 23), abstracted as a deterministic periodic oscillator. Other studies suggest these peri-threshold activity patterns to be arhythmic and stochastic (17, 19, 20), with theta-filtered noise as an illustrative abstraction (16). However, these frameworks do not account either for the pronounced cell-to-cell variability in ion channel and intrinsic properties of SCs (12, 14, 24) or for the stochasticity in ion channel and synaptic physiology (17, 19, 20). Here, accounting for both heterogeneities and stochasticity, we argue that neither the periodic oscillator nor the theta-filtered noise abstractions can fully explain peri-threshold oscillations in SCs. Instead, with several lines of evidence, we argue that these peri-threshold activity patterns and their characteristic features are fully explained by stochastic bifurcations in a heterogeneous neuronal population.

First, we performed in vitro electrophysiological recordings from rat SCs and found pronounced cell-to-cell variability in characteristic physiological measurements. These recordings showed characteristic oscillatory patterns with variable amplitude and frequency at disparate peri-threshold voltage ranges. Heterogeneities in electrophysiological recordings emphasized the need to employ a heterogeneous population of neuronal models to assess the theoretical underpinnings behind these oscillatory patterns. This is especially the case because biological neurons, including SCs, manifest ion-channel degeneracy, whereby disparate combinations of structural components could manifest similar characteristic physiological properties (12, 25, 26). Therefore, we employed a heterogeneous population of SC models that manifested degeneracy (12).

SC responses to depolarizing current injection with continually increasing amplitude transition from resting conditions to subthreshold oscillations to mixed-mode oscillations to continuous firing to depolarization-induced block. Mixed-mode oscillations refer to voltage traces that contain spikes riding atop a fraction of the peaks of coexistent subthreshold oscillations (5–11). In dynamical systems, a bifurcation is defined as an abrupt qualitative change in the topology or phase portrait in response to small smooth changes in the value of a bifurcation parameter (27). Each SC transition could therefore be considered as a bifurcation with injected current as the bifurcation parameter, and neurons are often modeled as dynamical systems manifesting bifurcations (28, 29). From a mechanistic standpoint, neuronal bifurcations are mediated by the gating properties and kinetics of the specific set of ion channels expressed in each neuron. As ion channels and synapses are fundamentally stochastic in their function, it is critical that neuronal bifurcations are not treated as deterministic bifurcations, but as stochastic bifurcations (9, 17, 19, 20).

To assess the impact of stochasticity on the emergence of oscillatory patterns, we introduced three different forms of noise (ion channel, synaptic, and additive) into the heterogeneous SC population. We observed the manifestation of heterogeneous stochastic bifurcations across the population of SC models, qualitatively demonstrating that noise could play a stabilizing role in yielding intrinsic oscillatory patterns. To quantify such beneficiary roles of noise, we developed five different quantitative metrics, derived from the spectrogram of activity patterns, to detect the presence of stable oscillatory traces. We set bounds on these measurements such that they were sufficient to capture the variable amplitude and irregular oscillatory patterns observed in rat SCs. We further assessed the reliability of spectrogram-based metrics in capturing the stability of the oscillations in a stochastic nonlinear dynamical system (Hopf bifurcation), employed here as an illustrative abstraction for SC oscillations. Validation of traces from the stochastic Hopf bifurcation system with different levels of noise provided a key quantitative insight into the manifestation of stochastic resonance (30). Specifically, our analyses of the stochastic Hopf bifurcation unveiled the presence of an optimal level of noise that facilitates stabilization of oscillatory patterns. In striking contrast, there was no stochastic resonance expressed with increasing noise levels in theta-filtered noise traces.

We performed this validation process on traces from the stochastic, heterogeneous population of SCs and found that noise-induced stabilization of peri-threshold oscillations occurred at an optimal level of noise. This expression of stochastic resonance provided a further line of evidence that activity patterns in SCs were consistent with stochastic bifurcations, and not with the theta-filtered noise abstraction. Within the heterogeneous stochastic bifurcation framework, stochasticity contributed to characteristic variability in amplitude and frequency of oscillatory patterns, while ion-channel heterogeneities across models translated to pronounced neuron-to-neuron variability in these patterns. The manifestation of stochastic resonance also explained why intrinsic oscillations have not been observed under in vivo conditions where noise levels are typically high (3, 21, 31). As the expression of peri-threshold oscillations under in vitro conditions could be attributed to ion-channel noise driving the stochastic bifurcations (17, 19, 20), we generated and validated an independent heterogeneous population of SC models with stochastically gated ion-channel models. Our analyses of these stochastic SC models confirmed the expression of ion-channel degeneracy in SCs and demonstrated the expression of stochastic resonance in the emergence of peri-threshold oscillations. Finally, we built a generalized simple model with a network motif (32) comprised of a slow negative feedback loop amplified by a fast positive feedback loop, and found this generalized simple system to manifest stochastic bifurcations and stochastic resonance in the emergence of oscillations.

Together, using a combination of theoretical, computational, and electrophysiological methods, we argue for heterogeneous stochastic bifurcations as a unifying framework that fully explains peri-threshold activity patterns in SCs. Within this framework, we argue that the manifestation of intrinsic oscillatory patterns in SCs should be considered as state-dependent, as several factors govern their emergence. These factors include heterogeneities in ion-channel composition and intrinsic properties, the overall synaptic drive that drives the bifurcation parameter, the levels of different forms of noise, neuromodulatory tone under different behavioral states, activity-dependent neural plasticity, and chan-nelopathies. From a broader perspective, the framework proposed here for the emergence of oscillatory patterns is a unique convergence of the degeneracy and the stochastic resonance frameworks, involving a generalized network motif comprising positive and negative feedback loops. Whereas the degeneracy framework provides an ideal substrate for achieving functional robustness through variable parametric combinations (12, 25, 26, 33), an optimal level of noise plays a beneficiary role in the emergence and stabilization of oscillatory patterns (30, 32). We postulate the convergence of these two frameworks as a universal template for the robust emergence of different biological phenomena across different scales of analysis.

## Results

The central hypothesis assessed in this study is that peri-threshold activity patterns observed in SCs are consistent with those elicited by stochastic bifurcations in a heterogeneous neuronal population.

### Electrophysiological Recordings from Rat SCs Manifested Heterogeneities in Characteristic Physiological Properties, Including Peri-Threshold Oscillations

**Fig. 1:**
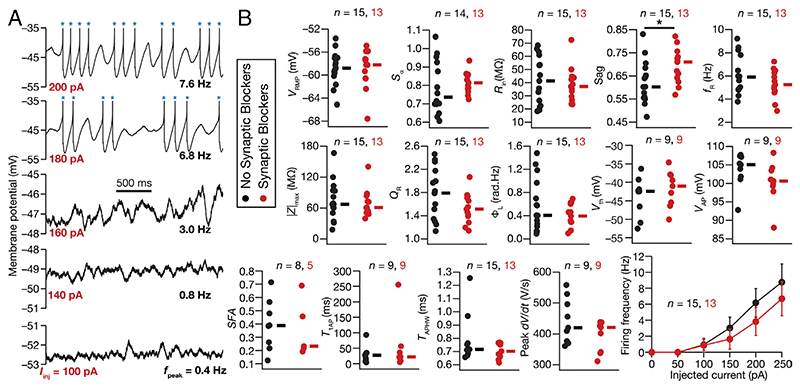
Heterogeneities in characteristic subthreshold and suprathreshold measurements from rat MEC SCs recorded using whole-cell patch clamp electrophysiology. (*A*) Example of peri-threshold membrane potential activity in the voltage response of the cell to different depolarizing pulse current injections (*I*_inj_). Note that when spikes occurred (blue asterisks), they were truncated to -35 mV to emphasize subthreshold dynamics. (*B*) Heterogeneities in 15 characteristic electrophysiological measurements of SC populations recorded with (red) or without (black) synaptic receptor blockers in the bath. Resting membrane potential, *V_RMP_*; input resistance, *R*_in_; temporal summation ratio, *S_a_*; Sag ratio, *Sag*; resonance frequency, *f_R_*; resonance strength, *Q*_R_; total inductive area, Φ_L_; AP half-width, *T*_APHW_; AP maximum slope, ${\frac{dv}{dt}|}_{\text{AP}}^{\max}$; AP threshold, *V*_th_; AP amplitude, *V*_AP_; spike frequency adaptation, *SFA*; latency to first AP, *T*_1AP_. None of the measurements other than *Sag* (*P* = 0.0341, Wilcoxonrank sum test) were significantly different between with vs. without synaptic blockers groups.

We performed two sets of intracellular recordings from visually identified rat SCs, in the presence or absence of synaptic receptor blockers. We characterized SCs using several intrinsic measurements (Fig. 1 and SI Appendix, Fig. S1) and confirmed that they manifested signature electrophysiological properties (Fig. 1B), especially the manifestation of peri-threshold oscillations with short- and long-pulse protocols (Fig. 1A and SI Appendix, Figs. S2 and S3). Importantly, we also found instances of spike clustering, a signature characteristic of SCs (8, 9, 11, 34), in peri-threshold mixed-mode oscillatory traces that contained spikes (Fig. 1A and SI Appendix, Figs. S2 and S3). The intrinsic properties of SCs manifested pronounced cell-to-cell variability, irrespective of whether recordings were performed in the presence (*n* = 13) or absence (*n* = 15) of synaptic blockers (Fig. 1B) and Tables S1 and S2). Consistent with earlier electrophysiological (18) and modeling (12) measurements, impedance phase manifested a lead in the lower frequency ranges, with the total inductive phase (Φ_L_) showing nonzero values (SI Appendix, Fig. S1E and Fig. 1B). These intrinsic measurements were dependent on the voltage at which recordings were performed (SI Appendix, Fig. S4A) and showed weak correlations across most pairs of measurements spanning all (*n* = 28) SCs (SI Appendix, Fig. S4 B and C). These weak correlations across most pairs confirmed that the different measurements employed here (SI Appendix, Fig. S1) were providing insights into different aspects of SC physiology.

### The Dynamics of a Nonlinear System Manifesting Stochastic Bifurcations Were Consistent with Peri-Threshold Activity Patterns of SCs

**Fig. 2:**
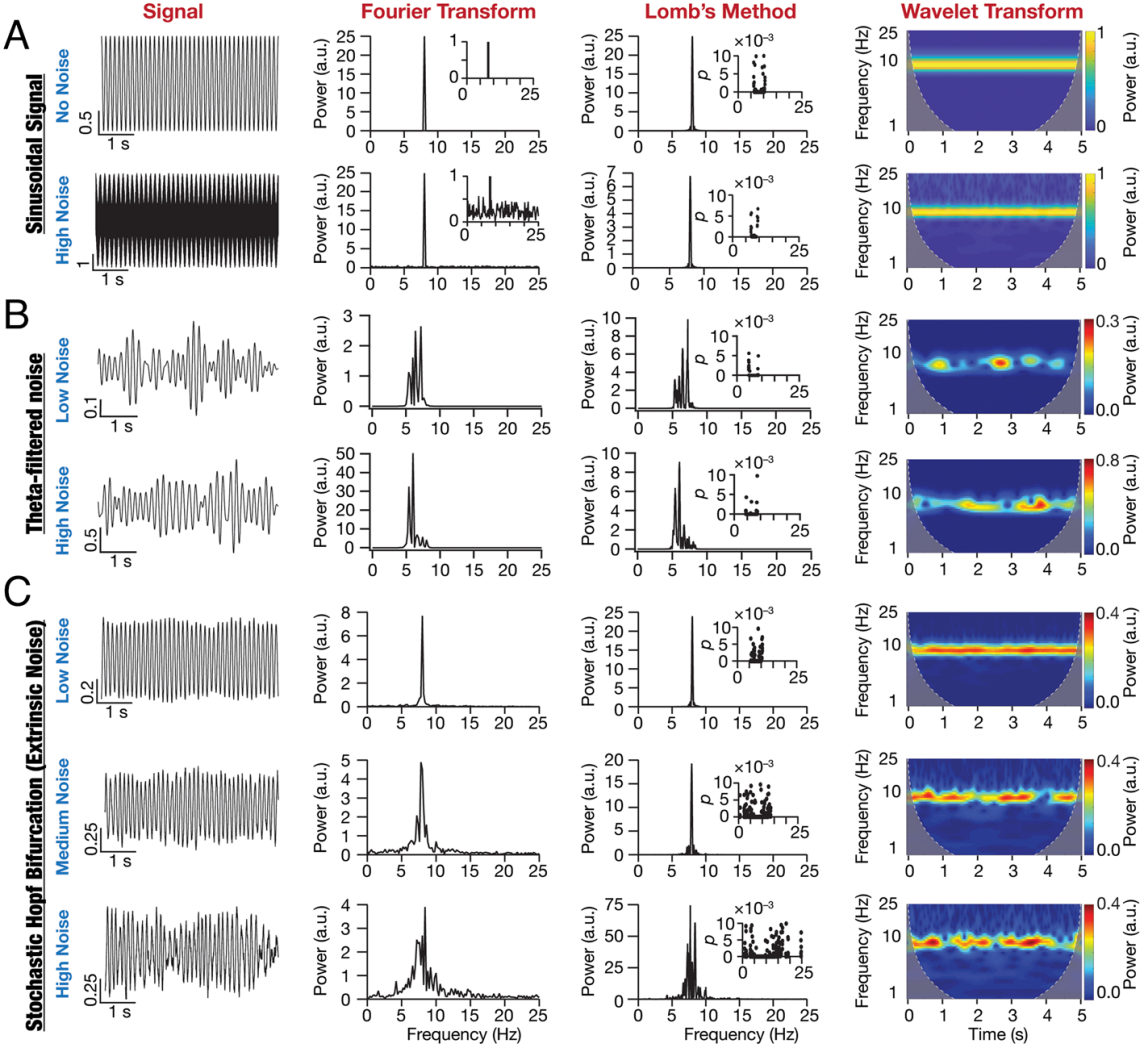
Oscillations emergent from a stochastic nonlinear dynamical system are illustrative abstractions of SC oscillations. (*A*) Impact of zero mean Gaussian white noise (GWN) on the frequency content of an 8 Hz sinusoidal signal. *Top row:* No noise, *Bottom row:* High noise. *Column 1:* time-domain signal, *Column 2:* Fourier transform (notice the 8 Hz peak) of the signal, *Column 3*: Lomb’s periodogram of the signal with *Inset* depicting the significance of each peak in the periodogram, and *Column 4:* spectrogram of the signal computed using wavelet transform. (*B*) Impact of altering the variance on filtered zero mean GWN traces. *Row 1:* Low noise and *Row 2:* High noise. *Columns 1 to 4:* same as panel *A. (C)* Impact of additive zero mean GWN (extrinsic noise) on oscillations emerging from a nonlinear dynamical system (Hopf bifurcation). *Row 1:* Low noise, *Row 2:* Medium noise, and *Row 3:* High noise. *Columns 1 to 4:* same as panel *A*.

An ongoing debate on the peri-threshold intrinsic activity patterns manifested by SCs (Fig. 1A and SI Appendix, Figs. S2 and S3) argues for them to be deterministic and rhythmic oscillatory patterns akin to a pure sinusoid (Fig. 2A) or consider them to be stochastic and arhythmic patterns (Fig. 2B). Different signal processing tools, including Fourier analysis, Lomb’s periodogram analysis, and wavelet spectrogram (Fig. 2 A and B), have been employed to assess these activity patterns for their consistency with either of these frameworks (8, 10, 13–18, 34, 35). Neurons in general (28, 29), and SCs in particular (8, 19, 22, 35), have been modeled as nonlinear systems manifesting bifurcations yielding stable limit cycles. Our central argument here is that perithreshold activity patterns in SCs are consistent with dynamical systems manifesting stochastic bifurcations. To systematically build our argument, we picked the Poincare–Andronov–Hopf bifurcation (equations 5 to 6), a simple nonlinear dynamical system that manifests a deterministic bifurcation yielding stable limit cycles, as an illustrative abstraction. This deterministic system switches between a no-oscillation state involving a stable spiral to an oscillatory state manifesting stable limit cycles with changes in a bifurcation parameter. The oscillatory patterns generated by this deterministic bifurcation system were rhythmic and translated to a single sharp peak in the spectral domain.

We introduced stochasticity into the system by means of extrinsic (equations 7 to 8) or intrinsic (equations 9 to 11) perturbations, which enabled the system to stochastically switch between different bifurcation states. The dynamics of this simple system manifesting stochastic bifurcations, with either extrinsic (Fig. 2C) or intrinsic (SI Appendix, Fig. S5) noise, were qualitatively and quantitatively consistent with subthreshold activity patterns from SCs (Fig. 1A and SI Appendix, Figs. S2 and S3). Specifically, qualitatively, these traces exhibited oscillations that were irregular, arhythmic, were of variable amplitude, and noisy (Fig. 2C and SI Appendix, Fig. S5). Quantitatively, we observed multiple peaks in their Fourier spectra, multiple statistically significant peaks in their Lomb’s periodogram, and their wavelet spectrograms reflected the variable amplitude structure (Fig. 2C and SI Appendix, Fig. S5). We emphasize that the Hopf bifurcation is an illustrative abstraction, akin to a pure sinusoid or theta-filtered noise, and not a model that captures the physiology of SCs.

### Different Forms of Noise Stabilized Peri-Threshold Oscillatory Activity in SC Models

Motivated by the ability of a simple system manifesting stochastic bifurcations to show activity patterns that were consistent with SC activity, we next introduced stochasticity into conductance-based SC models. SCs could be considered as a multi-dimensional nonlinear dynamical system endowed with multiple bifurcation states achieved with increasing values of injected current: resting state, subthreshold oscillations, mixed-mode oscillations (involving subthreshold oscillations and AP firing), regular AP firing, and depolarization-induced block. Specific sets of ion channels, their expression profiles, characteristic passive properties, and interactions among these different components together allow these neurons to manifest these signature bifurcation states (5–9, 11, 12, 16, 17, 19, 20).

**Fig. 3:**
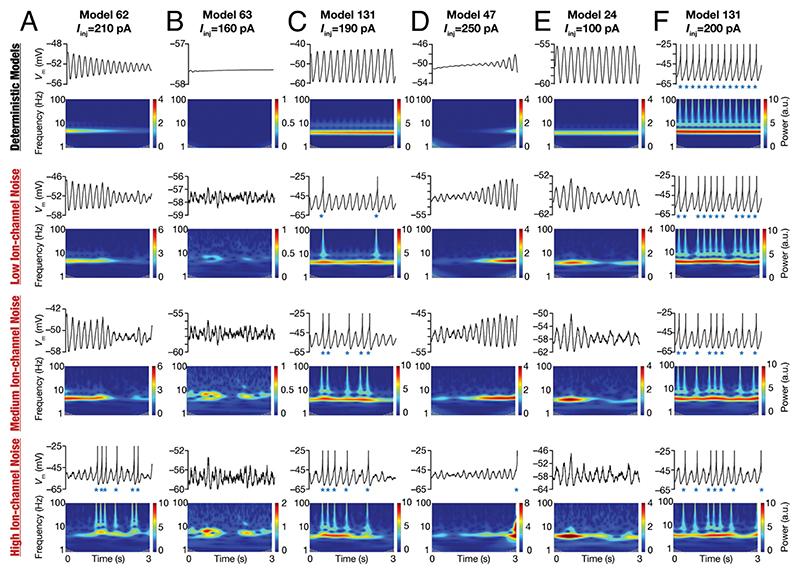
Illustrative examples depicting the role of ion-channel noise in stabilizing peri-threshold oscillatory patterns in a heterogeneous population of SC models. Each row in panels (*A*–*F*) depicts intrinsic activity patterns from different models for a 3-s period and the corresponding spectrograms computed using wavelet transform. Each column is identified by the corresponding model number along with identified values of injected current (*I*_inj_) employed to generate the activity patterns. The first row depicts activity patterns in the deterministic model in the absence of any form of noise. Rows 2 to 4 depict activity patterns from the same model, injected with the same *I*_inj_ value, with low (0.3), medium (1.2), and high (4.8) ion-channel noise. Note that when spikes occurred (blue asterisks), they were truncated to –25 mV to emphasize the subthreshold dynamics.

SCs manifest pronounced heterogeneities in their parameters and intrinsic properties (Fig. 1), including cell-to-cell variability in the injected current required for transitions across these bifurcation states (5–9, 11, 16, 20). To capture these heterogeneities in our model, we employed a heterogeneous population of 155 models that was generated previously (12) through an unbiased search and was validated against 10 different characteristic physiological properties of SCs. Importantly, the ranges of measurements and the interdependence among measurements obtained by electrophysiologically recorded SCs (Fig. 1 and SI Appendix, Fig. S4) were comparable to their counterparts in this heterogeneous SC model population (12). These 155 models also exhibited heterogeneities in all 55 parameters that governed ion channel, passive, and calcium-handling properties of the model, together expressing degeneracy in matching signature SC physiology (12). By virtue of the validation process, these deterministic models manifested theta-frequency oscillations at specific peri-threshold voltage ranges (12). However, there were some models that exhibited nonphysiological activity patterns at certain current injection values. For instance, there were deterministic models that manifested unstable oscillations with inward (Fig. 3A) or outward (Fig. 3 C and D) spirals.

We introduced three different forms of stochasticity into this heterogeneous population of SC models to assess the ability of noise to stabilize peri-threshold oscillations. Ion-channel noise was introduced to mimic intrinsic stochasticity in ion-channel gating. Synaptic noise impinged on the neuronal model as fluctuations imposed by balanced excitatory and inhibitory conductance-based synapses. Additive noise was injected as a current into the model to mimic external perturbations. The impact of these three forms of noise on a set of models manifesting heterogeneous peri-threshold activity patterns provided important insights about the stabilizing role of stochasticity (Fig. 3 and SI Appendix, Figs. S6 and S7). Specifically, deterministic models that manifested unphysiological and unstable oscillations that were decaying (Fig. 3A and SI Appendix, Figs. S6A and S7A) or expanding (Fig. 3 C and D and SI Appendix, Figs. S6 C and D and S7 C and D) amplitudes switched to arhythmic, variable amplitude oscillations. Deterministic models that did not manifest oscillatory patterns for a specific current injection showed arhythmic, variable amplitude oscillations with the introduction of noise (Fig. 3B and SI Appendix, Figs. S6B and S7B). Importantly, deterministic models that manifested regular subthreshold oscillations also switched to showing arhythmic, variable amplitude oscillations (Fig. 3E and SI Appendix, Figs. S6E and S7E). Certain deterministic models showing purely subthreshold dynamics switched to mixed-mode oscillations at certain levels of noise (Fig. 3 A–D and SI Appendix, Figs. S6 A–D and S7 A–D), whereby there were spikes riding on top of subthreshold variable amplitude oscillations (Fig. 3 and SI Appendix, Figs. S6 and S7). Deterministic models manifesting spiking also switched to mixed-mode oscillations with the introduction of stochasticity (Fig. 3F and SI Appendix, Figs. S6F and S7F). Strikingly, models that exhibited mixed-mode oscillations manifested theta skipping in their spiking activity, also resulting in the clustering of spikes (Fig. 3 and SI Appendix, Figs. S6 and S7) that is observed in SC activity (Fig. 1A and SI Appendix, Figs. S2 and S3).

Qualitatively, the heterogeneous population captured important stabilizing roles for stochasticity in yielding intrinsic oscillatory patterns in SC models (Fig. 3 and SI Appendix, Figs. S6 and S7) that were similar to their physiological counterparts (Fig. 1A and SI Appendix, Figs. S2 and S3): i) the ability to trigger oscillatory patterns even when oscillations are absent in the deterministic model; ii) the ability to convert decaying or expanding oscillations in the deterministic model to variable amplitude, arhythmic oscillations; iii) the ability to transform regular subthreshold oscillations in the deterministic model to arhythmic, variable amplitude oscillations; and iv) the ability to generate mixed-mode oscillations with theta-skipping and clustered spikes in deterministic models showing subthreshold or suprathreshold activity patterns. The specific intrinsic activity patterns and the switches between the different bifurcation states in these model neurons were driven by their intrinsic heterogeneities, the value of the bifurcation parameter (injected current), the form, and the level of noise. Together, these observations pointed to the manifestation of heterogeneous stochastic bifurcations across the population of SC models.

### Development of Quantitative Metrics Based on the Wavelet Spectrogram for Detecting Stable Oscillatory Activity

**Fig. 4:**
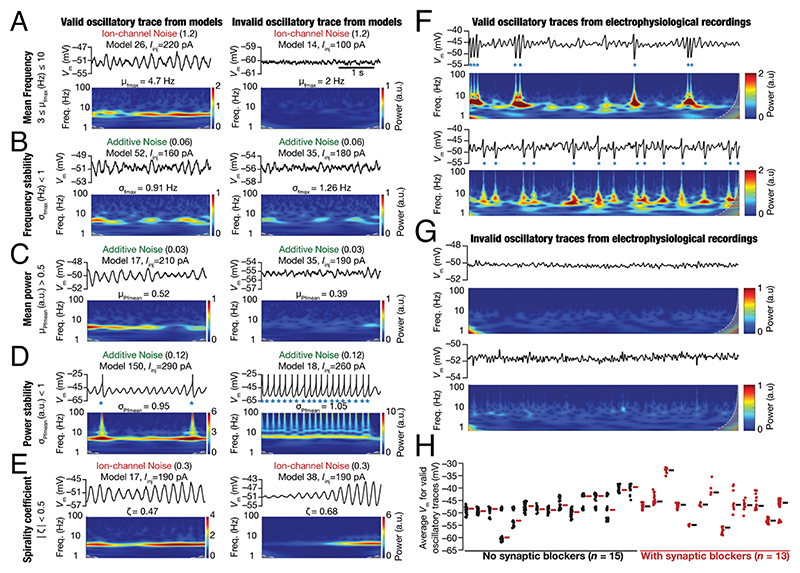
Spectrogram-based quantitative metrics used for assessing robustness in theta-frequency oscillatory activity in model outcomes and electrophysiological recordings. (*A*−*E*) Examples of valid (*Left*) and invalid (*Right*) oscillatory traces obtained from peri-threshold oscillatory patterns from different neuronal models at identified values of injected current (*I*_inj_). The form and the level of noise employed to generate the activity pattern are also provided. For each case, shown are the time-domain traces (*Top*) and the respective spectrogram computed through wavelet transform (*Bottom*). The example valid and invalid traces are shown with reference to each of the five spectrogram-based quantitative metrics for assessing robustness in theta-frequency oscillatory activity: (*A*) Mean frequency at maximal power, μ_fmax_; (*B*) SD of frequency at maximal power, *σ*_fmax_; (*C*) Mean power at mean frequency, μ_Pfmean_; (*D*) SD of power at mean frequency, *σ*_Pfmean_; (*E*) Spirality coefficient, *ζ*. (*F* and *G*) Examples of valid (*F*) and invalid (*G*) oscillatory voltage traces (15 s long) from electrophysiological recordings and their respective wavelet transforms. (*H*) Mean membrane potentials where valid oscillatory traces were observed for each cell, recorded in the presence (red, *n* = 13) or absence (black, *n* =15) of synaptic receptor blockers, highlighting the heterogeneity across this population of 28 distinct cells.

Although visual inspection of activity patterns obtained with noise qualitatively suggested a stabilizing role for noise in the emergence of peri-threshold oscillations, it was essential to develop quantitative metrics for detecting oscillatory patterns. The complexity of the validation task was enormous, considering the combinatorics of 155 different models, each assessed at 21 different levels of depolarizing current values, with 10 trials for several levels of three different forms of noise. Together, these yielded a total requirement of validating 458,955 model traces. We developed five different validation criteria on quantitative metrics from the spectrogram of these voltage traces, toward detecting stable thetafrequency oscillations. Representative valid and invalid oscillatory traces that respectively passed and failed each of these five criteria are shown in Fig. 4 A–E. Two measurements were derived from the frequency values at maximal power computed from the wavelet spectrogram. Specifically, at each time point, we noted down the frequency at which maximal power was observed, yielding an array (**F**_mp_) of frequency values spanning the duration of the trace (3 s). The mean frequency, μ_fmax_ (Fig. 4A), was computed as the average value of **F**_mp_ and was required to be within the theta-frequency range (3 to 10 Hz). We used the SD, *σ*_fmax_, of the array **F**_mp_ as a measure of frequency variability within the trace and required this to be < 1 Hz for frequency stability (Fig. 4B).

Three other measurements placed constraints on the power at the mean frequency. Here, we noted down the power at μ_fmax_ at each time point, yielding an array (**P**_*μ*f_) of power values of the same duration as the original trace (3 s). The mean power, μ_pfmean_, was computed as the average value of **P**_*μ*f_. μ_pfmean_ was required to be >0.5, setting a threshold on the minimal power in the oscillatory traces (Fig. 4C). The SD, *σ*_pfmean_, of the array **P**_*μ*f_ was a measure of power variability (Fig. 4D) and was required to be <1 for power stability. The final measurement that we defined to validate oscillatory traces was the spirality coefficient (*ζ*), computed as the slope of a linear fit of the plot of **P**_*μ*f_ vs. time. The absolute value of the spirality coefficient was required to be <0.5, designed to avoid inward or outward spirals (Fig. 4E) in valid oscillatory traces.

Quantitatively, voltage traces that satisfied all five criteria were classified as valid oscillatory traces showing robust theta-frequency peri-threshold oscillations: 3 < μ_fmax_ < 10 Hz; *σ*_fmax_ < 1 Hz, μ_pfmean_ > 0.5; *σ*_pfmean_ < 1: |*ζ*| < 0.5. Importantly, these metrics were derived from the spectrogram of the unfiltered original trace, thereby avoiding artifacts associated with assessing filtered traces (e.g., Fig. 2B). Of all valid oscillatory patterns, ~6% were purely subthreshold and ~55% showed few spikes (ratio between spike frequency and peri-threshold oscillation frequency <0.1; spike frequency ≤1 Hz). Of the remaining ~39%, ~38% had this ratio less than 0.8 and only ~1% of valid oscillatory traces had the spike frequency greater than the peri-threshold oscillation frequency. Importantly, in these 1% traces, spike bursts contributed to the ratio being above 1 and there were sub-threshold oscillatory cycles without spikes (SI Appendix, Figs. S8–S11). Thus, valid oscillatory traces manifested either pure subthreshold oscillations or mixed-mode oscillations where spikes occurred atop a fraction of sub-threshold oscillatory cycles.

### Validation of Peri-Threshold Oscillatory Traces Obtained from Electrophysiological Recordings of Rat SCs

The validation criteria defined above were designed to eliminate non-physiological, non-theta, and weak oscillations, and imposed specific constraints on frequency and power stability. Were these criteria sufficient to capture the variable-amplitude and irregular oscillatory patterns observed in electrophysiological recordings of SCs? To directly assess this, we identified oscillatory traces from our electrophysiological recordings (Fig. 1A and SI Appendix, Figs. S2 and S3) that satisfied all validation criteria (Fig. 4 F–H and SI Appendix, Fig. S12). We observed pronounced neuron-to-neuron variability in the mean voltages (–60 to –30 mV), frequencies (3 to 10 Hz), and amplitudes of valid oscillatory traces (Fig. 4H and SI Appendix, Fig. S12). Valid oscillatory traces were subthreshold with the peak-to-peak amplitude <10 mV (1 to 10 mV range; 6.05 mV mean; 2.9 mV SD; SI Appendix, Fig. S12) or mixed-mode oscillations that elicited APs in some oscillatory cycles (Figs. 1A and 4F and SI Appendix, Figs. S2 and S3). There were ~17% valid oscillatory traces (~17% without synaptic blockers; ~18% with) that were purely subthreshold, and the remaining ~83% showed mixed-mode oscillations containing spikes (SI Appendix, Fig. S13). The characteristic features of these valid oscillatory traces across all recorded cells were assessed in raw traces or in traces that were median filtered to remove spikes, and manifested comparable heterogeneities in recordings obtained without or with synaptic blockers (SI Appendix, Fig. S12). Together, analyses of peri-threshold activity patterns from our electrophysiological recordings confirmed that the five quantitative criteria (Fig. 4 A–E) were sufficient to capture the characteristic variable-amplitude, noisy, and arhythmic oscillatory patterns in SCs (Figs. 1A and 4F–H and SI Appendix, Figs. S2, S3, S12, and S13).

### Manifestation of Stochastic Resonance in the Detectability of Valid Oscillatory Traces in the Stochastic Hopf Bifurcation System

To further validate the five metrics and to gain insights about the impact of noise on nonlinear dynamical systems showing bifurcations, we applied these validation criteria (Fig. 4 A–E) on traces obtained from the deterministic and stochastic Hopf bifurcations. We generated the temporal evolution traces from the Hopf bifurcation with different bifurcation parametric (*λ*) values and with different levels of stochasticity (SI Appendix, Fig. S14A). As stochasticity translates to considerable trial-to-trial variability in responses, we generated and validated 50 traces for each level of noise and each value of *λ*. In the deterministic Hopf bifurcation, stable limit cycles were observed in the dynamics with *λ* > 0 (SI Appendix, Fig. S14A; *λ* = 0.025; “No noise”) and were detected as a valid oscillatory trace. However, with *λ* ≤ 0, the deterministic system manifested decaying spirals (SI Appendix, Fig. S14A; *λ* = – 0.05, – 0.025, 0; “No noise”), which were not identified as valid oscillatory traces. Therefore, in the absence of noise, validation was binary depending on *λ:* no valid traces with *λ* ≤ 0 and all traces were valid when *λ* > 0.

Importantly, with an increase in noise levels, the decaying spirals in the deterministic system (when *λ* ≤ 0) switched to oscillatory patterns that manifested arhythmic and variable-amplitude oscillations (SI Appendix, Fig. S14A). As a consequence of these stochastic bifurcations introduced in the system with the presence of noise, the number of valid oscillatory traces increased with progressively higher noise levels when *λ* ≤ 0 (SI Appendix, Fig. S14B). However, this increase in the detectability of valid oscillatory traces was not monotonic, and there was a fall in the detectability of oscillatory traces beyond a certain optimal level of noise (SI Appendix, Fig. S14B, *λ* ≤ 0). This phenomenon involving the manifestation of peak performance at an optimal level of noise, with performance falling on either side of this optimal level, has been defined as stochastic resonance (30). With *λ* > 0, where stable oscillations were observed in the absence of noise, we observed a monotonic reduction in the number of valid oscillatory traces indicating the absence of stochastic resonance (SI Appendix, Fig. S14B).

As theta-filtered noise has been considered as an abstraction for activity patterns in the SCs, we assessed the impact of increasing noise intensity on theta-filtered noise traces. Specifically, we subjected Gaussian white noise (GWN) to band-pass filtering in the theta frequency range and validated the resultant traces with the five metrics (Fig. 4 A–E). We repeated this for 50 trials with different instances of the GWN and plotted the number of valid oscillatory traces as a function of GWN variance. Expectedly, and in striking contrast to stochastic bifurcations, filtered noise traces did not manifest stochastic resonance with increasing levels of noise, instead showed a saturating monotonic increase in the number of valid oscillatory traces with increasing noise levels (SI Appendix, Fig. S14C). These analyses provide a clear quantitative demarcation between filtered noise and a system manifesting stochastic bifurcations in terms of the expression of stochastic resonance in response to increasing noise levels.

Together, these analyses further confirmed the reliability of the spectrogram-based metrics in capturing the stability of the oscillations in a stochastic nonlinear dynamical system. Importantly, these analyses showed that an optimal level of noise facilitates stabilization of oscillatory patterns in stochastic bifurcation systems, but not with the theta-filtered noise abstraction. These results provided a quantitative handle to assess whether SC activity patterns are consistent with filtered noise or with stochastic bifurcations.

### The Ability of SC Models to Exhibit Deterministic Subthreshold Oscillations Translated to a Greater Number of Valid Oscillatory Traces with the Introduction of Noise

Our original goal was to quantitatively analyze activity traces from the 155 SC models, validating them for the presence of oscillations. We were now equipped with quantitative metrics for validation of oscillatory traces (Fig. 4 A–E), the reliability of which was verified with electrophysiological recordings (Fig. 4 F–H and SI Appendix, Fig. S12) and with oscillations in a nonlinear dynamical system (SI Appendix, Fig. S14 A and B). Thus, to quantitatively assess the stabilizing role of noise, we first computed the five quantitative metrics on all activity traces and plotted their distributions for each form of noise (SI Appendix, Fig. S15). We found 28.1% (129,309/458,955) of all oscillatory traces to satisfy the validation bounds on all five metrics. Although there were traces that failed each of the five criteria, the proportions of traces that failed the frequency stability and the minimum power criteria were the highest (SI Appendix, Fig. S15). Importantly, with the introduction of noise, there were only a small proportion of traces that manifested inward or outward spirals, with most traces satisfying the spirality constraint (SI Appendix, Fig. S15).

The distributions of the five metrics (SI Appendix, Fig. S15) were derived from a population of 155 neurons that manifested deterministic subthreshold oscillations (referred to as *θ*+ models; SI Appendix, Fig. S16A). Would these distributions be different if the deterministic models did not manifest subthreshold oscillations, but satisfied all the other characteristic physiological properties of SCs (referred to as *θ*− models; SI Appendix, Fig. S16A)? Would *θ*− models, which do not manifest deterministic subthreshold oscillations, switch to showing valid oscillatory traces with the introduction of noise? To address these questions, we exploited the advantages of the multi-objective validation procedure in generating the deterministic SC models. Specifically, we randomly picked 155 deterministic models that satisfied nine of the 10 characteristic physiological measurements that they were matched against (SI Appendix, Fig. S16B), but did not manifest theta-frequency subthreshold oscillations (SI Appendix, Fig. S16A). The number of *θ* – models was set as 155 to match with the 155 *θ*+ models. We then generated 458,955 activity traces from these *θ*− models, identical to the number of traces generated from *θ*+ models. We computed the five quantitative metrics on all traces from *θ*− models and compared their distributions with their counterparts from *θ*+ models (SI Appendix, Fig. S16A). Overall, there was a three-fold reduction in the number of valid oscillatory traces generated from *θ*− models (42,477 out of 458,955) compared to their *θ*+ counterparts (129,309 out of 458,955), owing largely to the overall low power of oscillations in traces from *θ*− models (SI Appendix, Fig. S17).

Together, the existence of a bifurcation state that manifested subthreshold oscillations in the deterministic model (*θ*+ models) translated to enhanced propensity of the model expressing peri-threshold oscillatory activity in the presence of noise. The absence of a bifurcation state that manifested subthreshold oscillations in the deterministic models (*θ*− models) resulted in peri-threshold activity patterns that were of lower power.

### Stochastic Resonance in the Emergence of Peri-Threshold Oscillatory Activity in SC Models

**Fig. 5:**
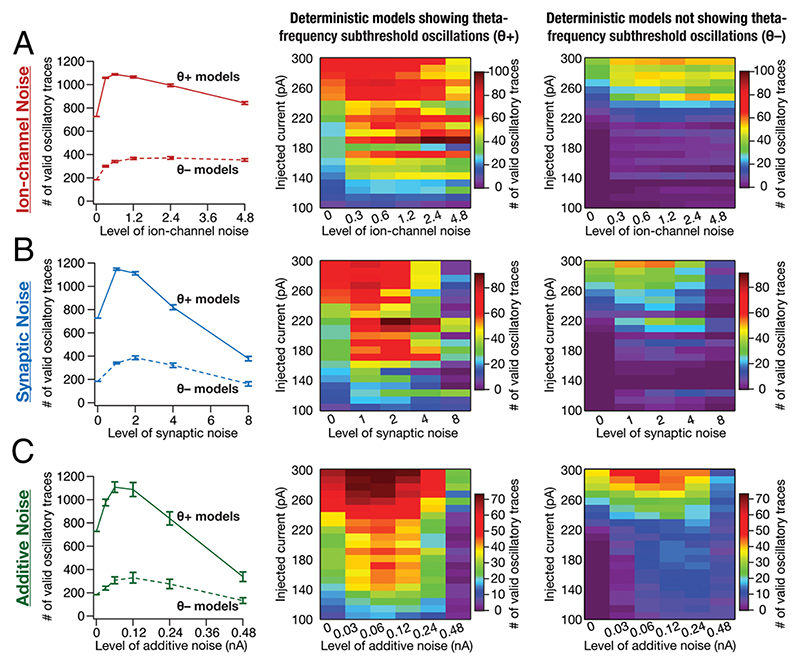
Stochastic resonance in the emergence of peri-threshold oscillations in a heterogeneous population of SC models. (*A–C*) *Left*, Mean and SEM of number of valid oscillatory traces from all *θ*+ (*n_θ+_* = 155) and *θ*− (*n_θ-_* = 155) model neurons. The number of valid oscillatory traces for deterministic (no noise) *θ*− models was non-zero because *θ*− models were picked based on the absence of subthreshold theta-frequency oscillations, implying a requirement that there were no spikes. However, the five criteria in Fig. 4 A–E employed for validating oscillatory traces were designed to also validate mixed-mode oscillations that manifest spikes. *Middle*, Number of valid oscillatory traces spanning all 21 current injection (*I*_inj_) values across different levels of noise across all *θ*+ model neurons. *Right*, Same as the *Middle* panel, but for *θ*− model neurons. The number of valid oscillatory traces is plotted as mean and SEM spanning 10 independent trials for each level of the three forms of noise: (A) ionchannel noise, (B) synaptic noise, or (C) additive noise, for all *θ*+ and *θ*− models. Note that valid oscillatory traces in *θ*− models were largely confined to higher current injection values between 220 and 300 pA. Total number of valid traces: ion-channel noise, *θ*+ (50,509/162,750) and *θ*− (17,320/162,750); synaptic noise, *θ*+ (34,588/130,200) and *θ*− (12,112/130,200); additive noise, *θ*+ (43,485/162,750) and *θ*− (12,861/162,750); deterministic models (0 noise), *θ*+ (727/3,255) and *θ*− (184/3,255).

We had earlier demonstrated the expression of stochastic resonance with increased levels of noise in a system manifesting stochastic bifurcation, but not with filtered noise (SI Appendix, Fig. S14 B and C). The expression of stochastic resonance in the detectability of valid oscillatory traces from SC models would provide evidence for the manifestation of stochastic bifurcations. To assess this, we plotted the number of valid oscillatory traces observed with each level of noise applied to the heterogeneous population of *θ*+ and *θ* – models (Fig. 5). Strikingly, we found that an optimal level of noise enhanced stabilization of oscillatory traces from both *θ*+ and *θ*− model populations. This manifestation of stochastic resonance covered all forms of noise and different injected current values (Fig. 5 A–C). Although traces from *θ*− models manifested stochastic resonance, the total number of valid traces at each noise level was considerably lower compared to those from *θ*+ models (Fig. 5 A–C). Importantly, at high levels of synaptic or additive noise within the tested range, the number of valid oscillatory traces was lower compared to the deterministic scenario where there was no noise (Fig. 5 B and C).

### Stochastic Resonance in the Emergence of Peri-Threshold Oscillatory Activity in Individual SC Models

**Fig. 6:**
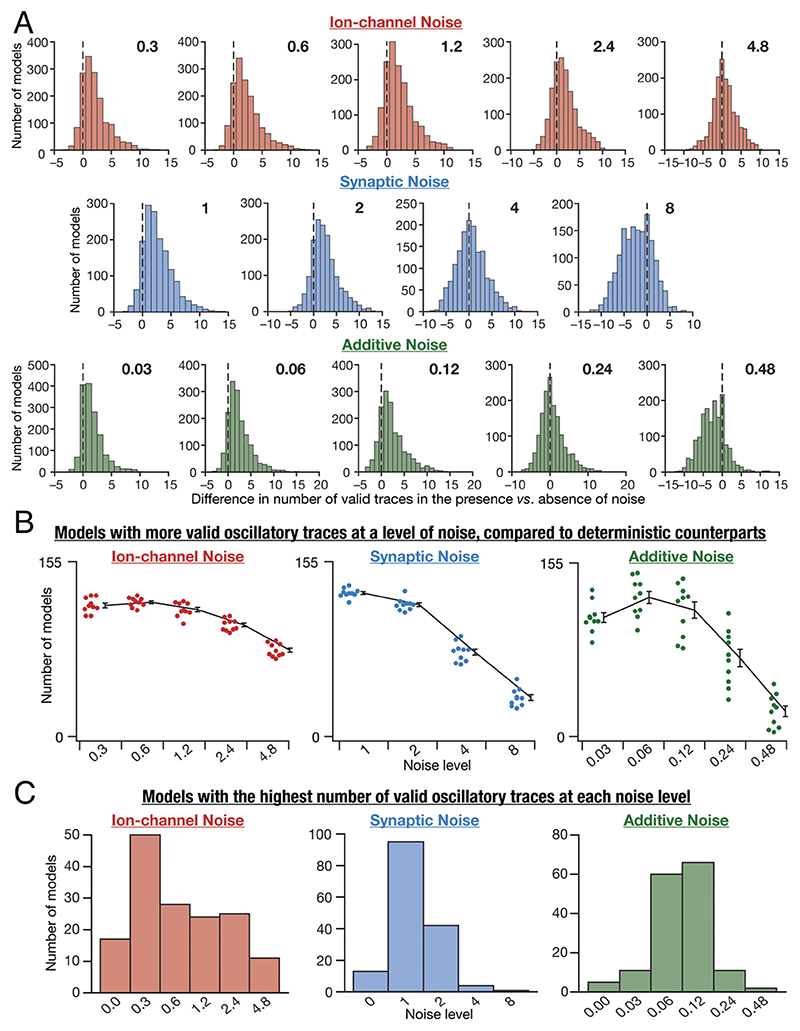
Stochastic resonance in the emergence of peri-threshold oscillations in individual SC models. (*A*) Histograms of the differences in the number of valid oscillatory traces in the presence vs. the absence of noise. Positive differences indicate a beneficial impact of noise on the emergence of oscillations. Histograms are shown for each level of noise (different columns) for all three forms of noise (different rows). These histograms indicated pooled data from 10 different trials for each level of all forms of noise, across all 155 *θ*+ models. (*B*) Summary data derived from panel *A*, showing the number of models that yield more valid oscillatory traces in the presence of noise (compared to deterministic models with no noise), at each of the different levels of all forms of noise. The individual data points represent each of the 10 trials for a given level of noise, and the summary statistics are represented as mean and SEM across trials. (*C*) The number of neuronal models (out of the maximum possible 155 *θ*+ models) exhibiting the highest number of valid oscillatory traces at each level of the three forms of noise. Noise level 0 indicates deterministic models. The maximum for each model was computed by considering the number of valid oscillatory traces in the model for all levels of a specific form of noise.

Our analyses of the manifestation of stochastic resonance in observing valid peri-threshold oscillations in SC models were at the population level, involving traces from all models (Fig. 5). Thus, these analyses did not provide evidence for the existence of an optimal noise level that increases the probability of observing peri-threshold oscillations in individual neurons. To address this, we performed two sets of analyses assessing the number of valid traces in individual models for different forms and levels of noise (Fig. 6 A and B).

As each model was assessed at 21 different current values, 21 is the maximum number of valid oscillatory traces from an individual model for a given noise level of a specific form of noise, a bound that holds for the zero-noise deterministic scenario as well. Let $N_{\det}^{i}$ and $N_{\text{noise}}^{i}$ be the number of valid oscillatory traces in model *i* in the absence (deterministic) or presence of noise, respectively. We computed the difference $N_{\text{noise}}^{i}-N_{\det}^{i}$ for all *θ* + models (1 ≤ *i* ≤ 155) for 10 trials at each level of the three forms of noise and plotted the distributions of these differences spanning all models and trials (Fig. 6A). These distributions could theoretically span the range from –21 to +21, with positive and negative values respectively indicating beneficiary and deleterious impact of noise on stable oscillations. A significant proportion of models manifested more oscillatory traces in the presence of noise, across all 10 trials, indicating a role for noise in stabilizing oscillation (Fig. 6B). Importantly, the number of neurons with positive values for $\left(N_{\text{noise}}^{i}-N_{\det}^{i}\right)$ was the highest at optimal noise levels, with number falling on either side, together providing evidence for the expression of stochastic resonance at the single-neuron level (Fig. 6 A and B).

Second, we noted down the noise level at which each model showed the highest number of valid oscillatory traces, with the maximum computed across all levels of a given form of noise for that model. We noted that different models showed the maximal number of valid oscillatory traces for different levels of noise, thus demonstrating heterogeneities in the specific value of the optimal level of noise across models (Fig. 6C). We binned all 155 *θ*+ models based on the specific noise level that they showed the maximum number of valid oscillatory traces (Fig. 6C). We observed that very few models showed maximal number of valid oscillatory traces in the absence of noise, thus emphasizing a beneficiary role for noise in the manifestation of peri-threshold oscillations (Fig. 6C). Notably, these analyses also unveiled neuron-to-neuron heterogeneities in the optimal level of noise required for achieving the maximum number of peri-threshold oscillatory traces in individual neurons.

As a final line of evidence, we normalized the number of valid traces in each model with reference to the maximum number of valid traces spanning all noise levels of a specific form of noise. This allowed us to account for the heterogeneities across different models, whereby each model manifested different numbers of maximal valid oscillatory traces at disparate levels of noise (Fig. 6C). By normalizing the number of oscillatory traces across all noise levels for individual models, we arrived at a plot for each model which was 1 at the specific noise level where it attained its maximal value and would be ≤1 at other noise levels. This normalization ensured that there was no domination by models with a greater number of valid traces. We collated these plots for each of the 155 *θ*+ models and computed their mean for each noise level (SI Appendix, Fig. S18A). Consistent with our earlier conclusions (Fig. 6), we found that the average fraction of valid traces in individual models was highest at an optimal level of noise, for all three forms of noise (SI Appendix, Fig. S18A). Although there were heterogeneities in the frequency of oscillations across traces, we found frequency ranges to be comparable across noise forms and levels (SI Appendix, Fig. S18B).

### Virtual Knockout Analyses: Role of Individual Ion Channels in the Emergence of Peri-Threshold Oscillatory Activity of SCs

**Fig. 7:**
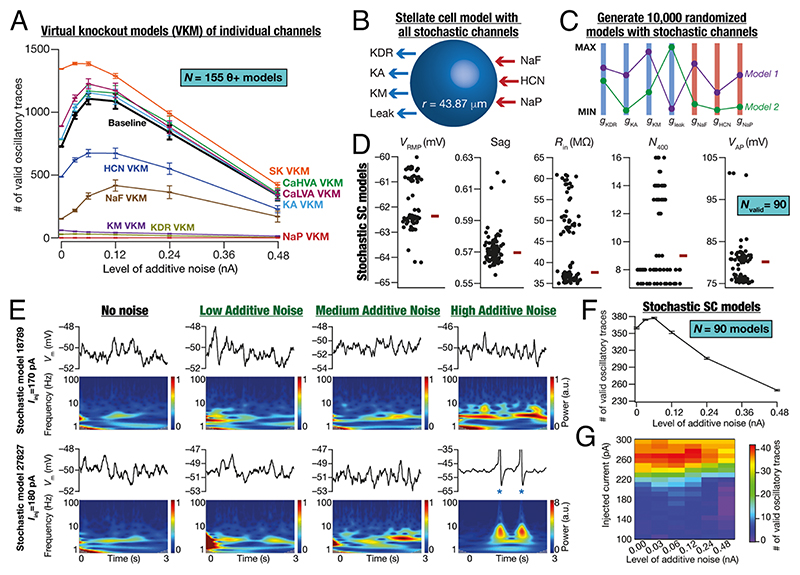
Minimal SC models endowed with stochastically gated ion channels manifested ion-channel degeneracy and stochastic resonance in the emergence of peri-threshold oscillations. (*A*) Mean and SEM of number of valid oscillatory traces from all *θ*+ (*n*_θ_+__ = 155) model neurons at six different levels of additive noise, each for 10 independent trials. For each virtual knockout, the entire run was repeated for 21 current injection values and six distinct additive noise levels, each set with 10 independent trials. The baseline model depicts the scenario where all ion-channels were intact and is the same as the trace shown in Fig. 5C for *θ*+ models. (*B*) Description of the minimal SC model endowed with all stochastically gated ion channels. (*C*) An independent multi-parametric multi-objective stochastic search was performed spanning seven different parameters (SI Appendix, Table S3), with 10,000 randomized models. Illustration of two randomized models shown to be sampled from the 7-dimensional parametric space. (*D*) Validation against six electrophysiological measurements (SI Appendix, Table S4) yielded a heterogeneous population of SC models (*N*_valid_ = 90). (*E*) Illustrative examples of the role of different levels of additive noise (low noise, 0.06 nA; medium noise, 0.12 nA; high noise, 0.24 nA) in stabilizing peri-thresholds oscillatory patterns in two models (different rows) derived from the heterogeneous population of SC models with stochastic gating ion channels. Each panel depicts intrinsic activity patterns (*Top*) from a given model for the specified step current injection (*I*_inj_) over a 3-s period and the corresponding spectrogram (*Bottom*) computed using wavelet transform. Note that when spikes occurred (blue asterisks), they were truncated to –35 mV to emphasize the subthreshold dynamics. (*F*) Mean and SEM of number of valid oscillatory traces from all SC models (*N* = 90) with stochastic gating ion channels, computed at six different levels of additive noise for 10 independent trials. (*G*) Number of valid oscillatory traces spanning all 21 current injection (*I*_inj_) values across different levels of noise across all stochastic SC neurons.

How do different ion channels contribute to the emergence of oscillatory activity at different noise levels? To address this, we performed analyses on virtual knockout models (VKM) (12, 36) on all *θ*+ (*n*_*θ*+_ = 155) models. Specifically, for each *θ*+ model, a specified conductance was independently set to zero and the peri-threshold activity was recorded, for 21 current injection values and six different levels of additive noise, with 10 independent trials for each pair. This process was repeated for all the nine active ion channels independently, and all the recorded traces were validated using our criteria (Fig. 4 A–E). Strikingly, we found the expression of stochastic resonance in the emergence of oscillations across all VKMs (HCN, NaF: fast sodium, KA: *A*-type K^+^, LVA Ca: low-voltage activated Ca^2+^, HVA Ca: high-voltage activated Ca^2+^, and SK: small-conductance calcium activated K^+^ VKMs) where valid oscillatory traces were observed (Fig. 7A). Consistent with prior literature (12, 37, 38), there was a complete loss of valid oscillatory traces with NaP VKMs and deleting HCN and *M*-type K^+^ (KM) channels had a major impact on the emergence of peri-threshold oscillatory activity (Fig. 7A). Virtual knockout of spike generating conductances (NaF and KDR: delayed rectifier K^+^) resulted in a major reduction of valid oscillatory traces, predominantly due to incomplete repolarization or insufficient depolarization. Virtual knockout of KA, LVA, HVA, and SK channels appeared to have minimal effect on the emergence of oscillatory activity, although they played modulatory roles in the emergence of oscillations (Fig. 7A and SI Appendix, Table S5). Importantly, these analyses extend earlier results on ion-channel degeneracy in SCs (12) to stochastic oscillatory activity, demonstrating a many-to-one mapping between ion channels and MPOs.

### Stochastic Resonance in the Emergence of Peri-Threshold Oscillatory Activity of SCs with Stochastically Gated Ion Channels

Our analyses thus far involved conductance-based models with deterministic ion-channel gating kinetics, except for scenarios where ion-channel noise was introduced into NaP dynamics artificially through a GWN. However, biological ion channels are stochastic in nature, with the stochasticity driven by their respective gating kinetics. Do neurons endowed with stochastically gated ion channels also exhibit stochastic bifurcations and manifest stochastic resonance? To address this, we developed a minimal stochastic SC model that contained the core set of ion channels essential for the emergence of peri-threshold oscillations (inferred from Fig. 7A) and other electrophysiological properties (12). We built models with stochastically gated versions of seven ionchannel subtypes (Fig. 7B), whose gating properties and kinetics were identical their deterministic counterparts (12). Consistent with our heterogeneities-based approach of assessing SCs and their oscillations, we performed an unbiased stochastic search on these seven ion channel the conductances (SI Appendix, Table S3) to find valid models (Fig. 7C). We generated 10,000 unique randomized models and validated them against six electrophysiological properties of SCs (SI Appendix, Table S4) to find 90 valid stochastic SC models (Fig. 7D). These stochastic SC models manifested heterogeneities in their intrinsic properties within the valid range (Fig. 7D) and manifested ion-channel degeneracy where disparate combinations of ion channels could elicit characteristic electrophysical properties (SI Appendix, Fig. S19), extending our prior conclusions on ionchannel degeneracy (12) to stochastic SC models. Identical to our earlier analyses with deterministic SC models (Fig. 5), we introduced different levels of additive noise to this heterogeneous population of stochastic SC population of models with 21 different current injections and validated them (Fig. 4 A–E). Our analyses confirmed the expression of stochastic bifurcations and stochastic resonance even in stochastic SC models (Fig. 7 F and G). The proportions of purely subthreshold vs. mixed-mode oscillations containing spikes in stochastic SC models (SI Appendix, Figs. S20 and S21) were comparable to their deterministic counterparts (SI Appendix, Figs. S8–S11). Notably, stochastic resonance was observed independently in valid sub-threshold and mixed-mode oscillatory traces from *θ*+ and stochastic SC models (SI Appendix, Fig. S22). Together, these provided further validation to our prior conclusions on ionchannel degeneracy and strengthen our postulate that peri-threshold oscillatory activity in SC models is consistent with heterogeneous stochastic bifurcations manifesting stochastic resonance.

### A Generalized Network Motif for Oscillatory Activity through Stochastic Bifurcations Manifesting Stochastic Resonance in the Emergence of Oscillations

**Fig. 8:**
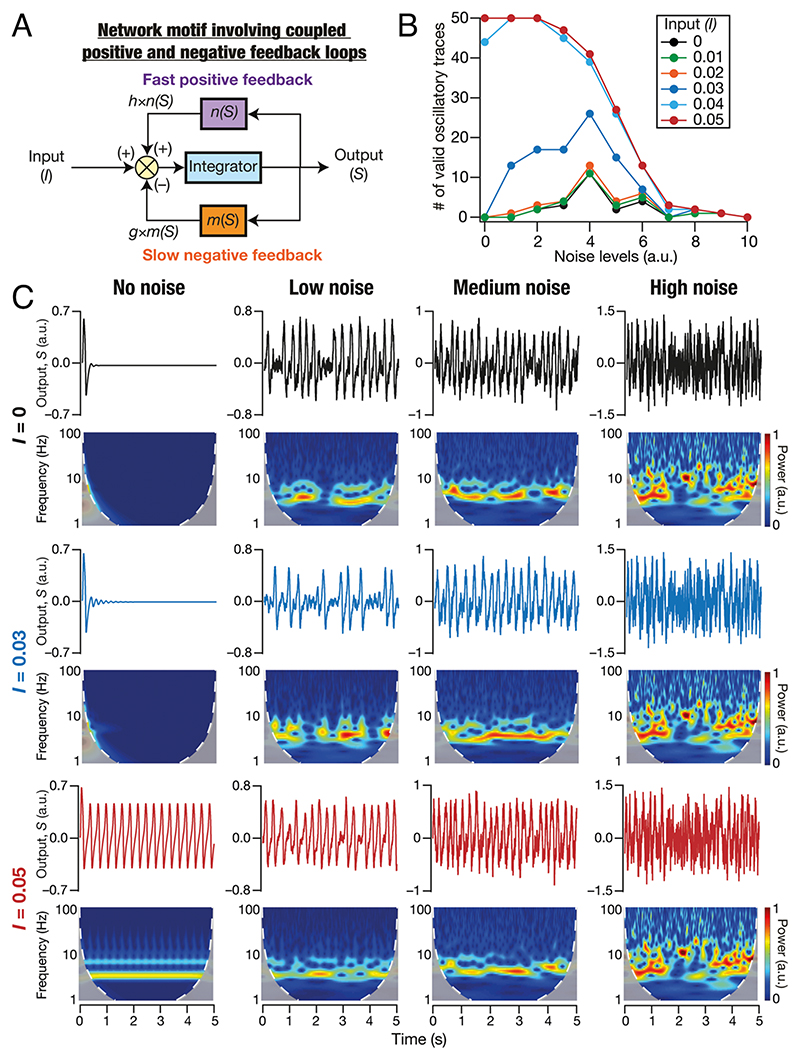
Simulations with an abstract network motif manifesting stochastic bifurcations expressed stochastic resonance in the emergence of peri-threshold oscillations. (*A*) A generalized network motif to describe intrinsic oscillatory activity. The model consists of integrator dynamics (mimicking RC circuit in neurons), a slow negative feedback loop (mimicking resonating conductances), and a fast positive feedback loop (mimicking amplifying conductances). (*B*) The number of valid oscillatory traces at different levels of GWN for various values of the bifurcation parameter *I*. Validation was performed on the outcomes of 50 trials for each value of *I* at different levels of noise. Note the manifestation of stochastic resonance when *I* ≤ 0.03: there is an optimal level of noise where the number of valid oscillatory traces is maximal, with the number falling on either side of this optimal level of noise. (*C*) Impact of different levels of GWN (Low, Medium, High) on the dynamics of the abstract model, shown for different values of the bifurcation parameter *I*. Note the emergence of stable oscillations in the deterministic system (“No Noise”) with *I* > 0.04, and inward spirals with *I* ≤ 0.03. Also note stochastic bifurcations resulting in manifestation of valid oscillatory traces in the presence of noise, even when *I* ≤ 0.03.

Intrinsic oscillatory activity in neurons is widely prevalent in other neuronal subtypes (39–42). To generalize the framework presented here to study the emergence and stability across other neuronal subtypes, we asked whether a generalized and simple network motif could reproduce the signature characteristics of the stochastic bifurcations-based framework. To this end, we built a model with a common network motif employed for achieving biological oscillations across scales (32): a slow negative feedback loop amplified by a fast positive feedback loop (Fig. 8A). In the cellular scale of analyses, the loops that are part of this universal motif for generating oscillations are implemented by ion channels (23, 43). The conductances mediating the slow negative feedback loop are called resonating conductances and those mediating the fast positive feedback loop are referred to as amplifying conductances (23). Our framework emphasizes the specific need to account for heterogeneity and stochasticity intrinsic to such a system or in external inputs to the system. To account for stochasticity, we introduced additive noise to this network motif and assessed the emergence of valid oscillations at different noise levels. We found that this generalized simple system manifested stochastic bifurcations (with the input to the system acting as the bifurcation parameter) and stochastic resonance in the emergence of oscillations (Fig. 8 B and C). We noted that this abstract model does not manifest mixed-mode oscillations as they are not endowed with an additional millisecond-scale pair of positive and negative feedback loops (with the negative feedback slower than the positive feedback) that mimic spike-generating conductances (NaF and KDR).

## Discussion

The principal goal of this study was to identify a theoretical framework that best describes the peri-threshold activity patterns observed in SCs (5, 11). It is evident from recordings from SCs that the deterministic periodic oscillator abstraction fails because activity patterns from these neurons manifest noisy oscillations with variable frequency, phase, and amplitude. In addition, there are lines of evidence for the absence of such oscillatory patterns in intracellular whole cell in vivo recordings from these SCs, conditions where they are typically faced with high noise levels (3, 31). However, if SC oscillations were theta-filtered noise, such high-noise conditions should instead have yielded stable oscillations (SI Appendix, Fig. S14C), thus providing evidence against filtered noise as an abstraction for these activity patterns. In this study, using a combination of theoretical, computational, and electrophysiological methods coupled with rigorous quantitative analyses (SI Appendix, Table S6), we argue for heterogeneous stochastic bifurcations as a unifying framework that explains all aspects of these peri-threshold activity patterns.

### Peri-Threshold Activity Patterns in SCs as Emergent Dynamics in a Heterogeneous Population of Neurons Manifesting Stochastic Bifurcations

We demonstrate that activity patterns in SCs, spanning different experimental conditions, can be explained by a theoretical framework that considers them as emergent dynamics of activity in a heterogeneous population of neurons manifesting stochastic bifurcations. These stochastic bifurcations were heterogeneous and manifested considerable variability, which was dependent on ion-channel heterogeneities, the level, and the form of noise. Our analyses unveiled the expression of stochastic resonance in the emergence of peri-threshold oscillations in deterministic (Figs. 5 and 6) and stochastic (Fig. 7) SC models with different noise. Importantly, there are lines of evidence for the expression of stochastic resonance in SCs from in vitro experiments. Specifically, it has been shown that additional ion channel, introduced under in vitro conditions using a dynamic clamp setup, plays a critical role in the emergence of peri-threshold oscillations (19, 20).

Heterogeneous stochastic bifurcations as the theoretical framework for explaining intrinsic patterns in SCs imply state dependence of both synaptic integration and the specific types of patterns emerging from these neurons. For instance, the expression of stochastic resonance provides a quantitative explanation for the absence of peri-threshold oscillations in intracellular in vivo recordings from SCs (3), where the noise levels are high beyond the optimal level of noise (Figs. 5 and 6) required for their emergence (21). Within this framework, under in vitro conditions, even with the blockade of synaptic receptors and the absence of additional external noise (Figs. 1A and 4 F–H and SI Appendix, Figs. S2, S3, and S12), the intrinsic ion-channel noise is sufficient to elicit peri-threshold oscillations with variable amplitude and frequency (Fig. 7 B–G). The rich diversity in peri-threshold activity patterns and how they emerge as functions of increasing noise levels, even with the same levels of noise across models, underscores the need to employ a heterogeneous population of models in assessing them (e.g., Fig. 3). A heterogeneous and stochastic population (Fig. 7 B–G) is essential in matching model outcomes with electrophysiological counterparts that manifest pronounced neuron-to-neuron variability and stochastic gating of biological ion channels.

The heterogeneous stochastic bifurcations framework also implies that synaptic integration in SCs depends on the composition and properties of ion channels in each cell, the form and level of noise encountered by individual compartments, and the specific timings and location of synaptic inputs (19–21). As stochastic bifurcations that are central to this framework are mediated by stochastic ion channels, differences in these oscillatory patterns with the blockade of different ion channels (Fig. 7A) are also readily explained within this framework (5, 6, 8, 9, 11, 12, 18, 34, 37, 38). Therefore, activity-dependent plasticity, neuromodulation, or channelopathies that affect the ion channels that are active in the peri-threshold range could alter these activity patterns.

### Degeneracy and Stochastic Bifurcations in Biological Systems

Degeneracy, the ability of disparate structural components to yield similar function, is a ubiquitous characteristic of biological system spanning all scales (33). From the perspective of single neuron function, the ability of different ion-channel combinations to yield characteristic physiological properties of neurons has been observed across different cell types (12, 25, 26). The expression of degeneracy has been argued as a mechanism to enhance biological robustness (25, 26, 33).

There are several examples within the dynamical systems literature showing a considerable impact of stochasticity on the bifurcations, including shift in the bifurcation points, introduction of secondary bifurcations, and manifestation of bistability (44–48). The stochastic fluctuations in microscopic ion-channel dynamics result in considerable differences in macroscopic properties and need to be specifically accounted for if they were to be matched with biological neuronal properties (16, 17, 19, 20, 49). The presence of stochastic bifurcations and stochastic resonance in biological systems has been studied not just at the cellular scale, but across all scales of biological systems from the perspective of bistability as well as in terms of stabilizing oscillatory properties (30, 32, 50–55). Thus, in assessing bifurcations in neural activity patterns, it is important that they are not mapped onto deterministic bifurcations emergent from macroscopic models of ion-channel function, but as stochastic bifurcations that account for fluctuations in microscopic components. Deterministic models that deal with macroscopic dynamics in a deterministic fashion run the risk of not matching biological properties under different contexts with distinct forms of extrinsic and intrinsic perturbations.

The framework proposed here to explain SC peri-threshold activity is a unique convergence of the degeneracy and the stochastic resonance frameworks. The heterogeneous ion-channel composition in the model population is a consequence of the expression of ion-channel degeneracy, where different ion-channel combinations yielded characteristic physiological properties (12). Whereas ion-channel degeneracy in SC models with deterministic ion-channel gating models was demonstrated earlier, our analyses here extend the manifestation of ion-channel degeneracy (with reference to several signature properties, including intrinsic oscillations) to SC models with stochastically gated ion-channel models (Fig. 7 and SI Appendix, Fig. S19). Such heterogeneous ion-channel composition across neurons allowed the deterministic model population to manifest different kinds of peri-threshold activity patterns [regular subthreshold oscillations, regular spiking, no oscillations, decaying oscillations, or expanding oscillations (e.g., Fig. 3)] at different current injections. In other words, heterogeneities in ion-channel properties in a population of neurons translated to heterogeneities in the emergence of bifurcation states across neurons. These heterogeneities allowed us to effectively account for the neuron-to-neuron variability observed in physiological characteristics and oscillatory patterns in electrophysiological recordings from SCs (Figs. 1A and 4 F–H and SI Appendix, Fig. S2, S3, and S12).

The strength, the form, and the specific instance of noise, and interactions of noise with heterogeneous bifurcation states across neurons added additional layers of variability in how these oscillatory patterns emerged. Importantly, the manifestation of maximum number of valid oscillatory traces with an optimum level of noise (Figs. 5–7) emphasized the beneficiary roles of noise in stabilizing oscillations emergent from heterogeneous bifurcations across models. Thus, the expression of ion-channel degeneracy in heterogeneous deterministic (Figs. 5 and 6) or stochastic (Fig. 7 B–G) populations of neurons showing parametric variability and the presence of noise were central to our heterogeneous stochastic bifurcation framework. This unique convergence between the degeneracy and the stochastic resonance frameworks allowed us to fully explain all aspects of peri-threshold activity in SCs across disparate experimental conditions. We postulate the convergence proposed here, between the degeneracy and the stochastic resonance frameworks, as a general substrate for achieving biological robustness spanning different scales of analysis.

### Limitations and Future Directions

The computational complexity of assessing heterogeneous stochastic bifurcations in our neuronal model population was enormous, requiring each of the multiple models to be assessed at different current injections with different levels and forms of noise. Therefore, we had resorted to the use of single compartmental conductance-based models, accounting for all the channel kinetics and intrinsic properties of the SCs. However, to assess the impact of stochasticity and heterogeneity on neuronal activity patterns, it is essential that electrophysiological studies characterize dendritic ion-channel and intrinsic properties across the arbor of SCs. A heterogeneous population of morphologically realistic models could then be built to assess the impact of stochasticity (ion channel and synaptic noise) and heterogeneities (in biophysical, synaptic, and morphological properties) on location-dependent peri-threshold oscillatory patterns and synaptic integration. Such models would also enable assessment of context-dependence of SC physiology, with levels and forms of noise, activity-dependent plasticity of channels and receptors, neuromodulation, and pathological channelopathies driving the context for physiological changes. The morphologically realistic models also would enable the introduction of balanced synaptic noise in a location-dependent manner, thus providing a more detailed assessment of the impact of synaptic noise and balance therein on intrinsic activity patterns. Networks of such heterogeneous SC models, along with similar models for interneurons, could then be connected to explore the implications of heterogeneous stochastic bifurcations to different models of grid-patterned activity (1–3, 14, 16, 24, 31, 43).

Important electrophysiologically testable predictions from our study are the state-dependence and the expression of stochastic resonance in the emergence of peri-threshold oscillations in SCs. While it is impossible to achieve zero-noise conditions in biological neurons endowed with intrinsically stochastic ion channels, interventional experiments involving introduction of additional noise (19–21) or suppressing high synaptic noise conditions are feasible. Specifically, in vivo peri-threshold activity patterns could be recorded from SCs across the dorso-ventral axis under different behavioral states, in the presence and absence of synaptic receptor blockers. Importantly, it is critical that recordings are performed with multiple amplitudes of current injection (or synaptic drive), as this constitutes the bifurcation parameter within our framework. These activity patterns could then be subjected to our validation metrics to address questions of whether oscillations emerge when the high synaptic noise is suppressed, and if there are differences in dorsal vs. ventral oscillatory patterns in vivo. If there are more valid oscillatory traces in the presence of synaptic blockers, that would provide direct electrophysiological evidence for the expression of stochastic resonance. Similar in vivo electrophysiological experiments could be performed with blockers of different ion channels and neuromodulators to assess the number of valid oscillatory traces in their presence *vs*. absence. These experiments would provide evidence for the role of disparate ion channels in mediating state-dependent stochastic bifurcations that are postulated here to mediate peri-threshold oscillations.

## Materials and Methods

We used theoretical, computational, and electrophysiological methods to understand the mechanism behind the emergence of peri-threshold oscillatory activity in SCs. The detailed descriptions of the procedures are provided in SI Appendix. Briefly, all surgical and electrophysiological protocols were in strict compliance with protocols approved by Institute Animal Ethics Committee of the Indian Institute of Science, Bangalore, India. Single-neuron electrophysiological recordings were performed under current-clamp configuration at physiological temperatures (33 to 35 °C) using 350-μm-thick horizontal medial entorhinal cortical slices from 5 to 9-wk-old male Sprague Dawley rats. Signature sub- and supra-threshold measurements (including peri-threshold oscillatory activity patterns) were computed from the electrophysiological data recorded from SCs. A simple nonlinear dynamical system (Hopf bifurcation system) was used for the abstraction of deterministic oscillations. The effect of stochasticity on such bifurcating system was observed by introducing intrinsic or extrinsic sources of stochasticity. Last, different levels of noise were introduced into two heterogeneous model populations of SCs [with deterministic (12) or stochastically gated ion-channel models], which were independently validated against the biologically observed ranges of signature electrophysiological measurements. Peri-threshold oscillatory activity was assessed using spectrogram-based quantitative measurements developed as part of this study.

## Supplementary Material

Supplementary Information

## Data Availability

All data are available in the main text or the SI Appendix. The source code files used for generating model data are freely available at https://doi.org/10.5281/zenodo.7413666.

## Significance

Stellate cells of the medial entorhinal cortex manifest intrinsic oscillatory activity, which has been implicated in network physiology and plasticity. Current theories about the emergence of these oscillations do not jointly account for heterogeneities and stochasticity, two ubiquitous characteristics of biological systems. Analyses that set aside neural heterogeneities assume a unique ionic basis for the oscillatory activity, disregarding ion-channel degeneracy. Frameworks that ignore stochasticity result in unstable systems that collapse with the slightest perturbations. In this unified synthesis, we demonstrate that heterogeneous stochastic bifurcations can fully explain stellate cell oscillatory patterns. The heterogeneities account for cell-to-cell variability in intrinsic oscillations and their ionic basis. Stochasticity imparts stability to the variable amplitude oscillations through the manifestation of stochastic resonance.
