# Supplementary material for: Heterogeneous stochastic bifurcations explain intrinsic oscillatory patterns in entorhinal cortical stellate cells
Source: Proc Natl Acad Sci U S A. 2022 Dec 19;119(52):e2202962119. doi: 10.1073/pnas.2202962119 (PMC7613999; doi:10.1073/pnas.2202962119)
Supplement: Supplementary file 1 — Appendix 01 (PDF) [file pnas.2202962119.sapp.pdf]

PNAS
www.pnas.org

Supplementary Information for

Heterogeneous stochastic bifurcations explain intrinsic oscillatory patterns in entorhinal cortical stellate cells

Divyansh Mittal and Rishikesh Narayanan*

Rishikesh Narayanan*
Email: rishi@iisc.ac.in

This PDF file includes:

SUPPLEMENTARY MATERIALS AND METHODS
Figures S1 to S22
Tables S1 to S6
SUPPLEMENTARY REFERENCES

SUPPLEMENTARY MATERIALS AND METHODS

Electrophysiology: Brain slice preparation and whole-cell current clamp recordings.

Ethical approval. All experiments reported in this study were performed in strict compliance with the protocols cleared by the Institutional Animal Ethics Committee (IAEC) of the Indian Institute of Science, Bangalore.

For all the electrophysiological experiments, male Sprague Dawley rats of age 5–9 weeks were used. The rats were maintained in 12 hours light – 12 hours dark cycle. Food and water were provided *ad libitum*. Surgical and *in vitro* electrophysiology procedures followed previously established protocols in the laboratory (1-3) and are detailed below. Rats were anesthetized by intraperitoneal injection of a mixture of ketamine-xylazine. The onset of deep anesthesia was assessed by the cessation of toe-pinch response. After deep anesthesia, transcardial perfusion was performed with ice-cold cutting solution (composition: 2.5 mM KCl, 1.25 mM NaH_2PO_4, 25 mM $NaHCO_3$, 0.5 mM $CaCl_2$, 7 mM $MgCl_2$, 7 mM dextrose, 3 mM sodium pyruvate, and 200 mM sucrose; pH 7.3; Osmolarity: ~300 mOsm; and saturated with 95% O_2 and 5% CO_2). Decapitation was performed in the presence of the cutting solution and the brain was removed quickly. Near-horizontal middle entorhinal cortical (bregma –6.5 mm to –5.1 mm) slices of thickness of 350 μm were obtained using a vibrating blade microtome (Leica Vibratome) while the brain was submerged in oxygenated ice-cold cutting solution. Brain slices were carefully transferred to the holding chamber solution (composition: 125 mM NaCl, 2.5 mM KCl, 1.25 mM NaH_2PO_4, 25 mM $NaHCO_3$, 2 mM $CaCl_2$, 2 mM $MgCl_2$, 10 mM dextrose, and 3 mM sodium pyruvate) and incubated for 10 mins at 34 ℃, followed by incubation at room temperature for at least 45 mins. The holding chamber was continuously oxygenated with 95% O_2 and 5% CO_2 gas mixture.

A slice was transferred to the recording chamber and was continuously perfused with oxygenated (95% O_2 and 5% CO_2 gas mixture) ACSF (125 mM NaCl, 3 mM KCl, 1.25 mM NaH_2PO_4, 25 mM $NaHCO_3$, 2 mM $CaCl_2$, 1 mM $MgCl_2$, and 10 mM dextrose; pH 7.3; Osmolarity: ~300 mOsm) at flow rate of ~4 mL/min. Recordings were performed under whole-cell current clamp configuration at physiological temperature of 33–35 ℃, which was ensured by an inline heater that was part of a closed-loop temperature control system (Harvard Apparatus). The slice was visualized using a 10× objective for locating LII MEC and then visualized under 63× water immersion objective through a Dodt contrast microscope (Carl Zeiss Axioexaminer) to visually identify a stellate cell in layer II MEC. Whole-cell current-clamp recordings were performed from stellate cells using a BVC-700A (Dagan) amplifier. Borosilicate glass electrodes were pulled (P-97 Flaming/Brown micropipette puller; Sutter) from capillaries (1.5 mm outer diameter and 0.86 mm inner diameter; Sutter) to yield 3–7 MΩ tip resistance. These electrodes were filled with internal solution (120 mM K-gluconate, 20 mM KCl, 10 mM HEPES, 4 mM NaCl, 4 mM Mg-ATP, 0.3 mM Na-GTP, and 7 mM K_2-phosphocreatine; pH 7.3 adjusted with KOH; osmolarity ~300 mOsm). For experiments requiring blockade of synaptic events, a combination of synaptic receptor blockers was added in the bath solution: 10 μM 6-cyano-7-nitroquinoxaline-2,3-dione (CNQX), an AMPA receptor blocker; 10 μM (+) bicuculline and 10 μM picrotoxin, both $GABA_A$ receptor blockers; 50 μM D,L-2-amino-5-phosphonovaleric acid (D,L-APV), an NMDA receptor blocker, and 2 μM CGP55845, a $GABA_B$ blocker. All synaptic blockers were procured from Allied Scientific.

For identification of stellate cells, three properties of these cell types were considered: the distance (100–150 μm) from the edge of the slice, the size of stellate cells are larger compared to interneurons or LIII EC pyramidal neurons, and range of characteristic electrophysiological measurements (depolarized resting membrane potential; large sag potential; low values of intrinsic excitability measurements: input resistance, impedance amplitude, firing frequency, and temporal summation; strong frequency selectivity in the theta-frequency range: 3–10 Hz) which were computed online during the course of the experiment.

All data acquisition was done using custom-written software in the Igor Pro (Wavemetrics) environment. Unless mentioned otherwise, the sampling rate was set at 40 kHz. Series resistance was continuously monitored and compensated using the bridge balance circuit in the amplifier. Cells were discarded from final analyses if the initial RMP was more depolarized than –50 mV or if the series resistance increased above 25 MΩ, or if there were temperature fluctuations observed during the experiment. All the experiments were performed at the initial resting membrane potential of the specific cell, unless otherwise specified. Voltages have not been corrected for the liquid junction potential, which was experimentally measured to be ~8 mV. Analyses and plotting of electrophysiological data were performed using custom-written programs

in IGOR Pro (WaveMetrics) and in MATLAB 2018a (Mathworks). Statistical analyses of electrophysiological data were executed using the R computing and statistical package (R core Team 2013).

Electrophysiology: Subthreshold Measurements.

Resting membrane potential (V_{RMP}) was computed by averaging 11 trials of the membrane potential without any current injection for 100 ms. Input resistance (R_{in}) was calculated from the steady-state voltage response (after 700 ms of current injection) of the cell to the subthreshold current pulses of amplitudes spanning –50 pA to 50 pA in steps of 10 pA (Fig. S1*A*). The slope of a linear fit to this steady-state voltage-current plot was assigned as the input resistance of the cell (Fig. S1*A*). To compute temporal summation ratio (S_{α}), five alpha-excitatory postsynaptic potentials (α-EPSPs) with 50 ms interval were evoked by current injection with the following dynamics:

$$I_{\alpha} = I_{\max}\, t \exp(-\alpha t) \qquad (1)$$

with α=0.1 ms^{-1}. The temporal summation ratio (S_{α}) was calculated as the ratio of the amplitudes of the last and first α-EPSPs (Fig. S1*B*). For estimating the sag ratio, the voltage response to a hyperpolarizing step current of 100 pA amplitude for 500 ms was recorded. Sag ratio was computed as the $100 \times (1 - V_{\mathrm{ss}}/V_{\mathrm{peak}})$, where V_{ss} and V_{peak} denoted the steady-state and peak voltage deflections from V_{RMP}, respectively (Fig. S1*C*).

Stellate cells of layer II of MEC are within the oscillatory network. Hence, it is essential to study the excitability of these cells with time-varying inputs of different frequencies, rather than limiting excitability measurements to pulse currents (4-9). We used chirp stimulus (Fig. S1*D*), a sinusoidal current stimulus with constant subthreshold amplitude (200 pA peak to peak) with frequency linearly spanning from 0 to 15 Hz in 15 s (10), for characterizing the impedance profile of stellate cells. Frequency-dependent impedance, $Z(f)$, was calculated as the ratio between the Fourier transform of the voltage response to the chirp stimulus and the Fourier transform of the chirp stimulus. The magnitude of this complex quantity defined the impedance amplitude profile (Fig. S1*E*):

$$|Z(f)| = \sqrt{\left(\mathrm{Re}\left(Z(f)\right)\right)^2 + \left(\mathrm{Im}\left(Z(f)\right)\right)^2} \qquad (2)$$

where $\mathrm{Re}(Z(f))$ and $\mathrm{Im}(Z(f))$ were the real and imaginary parts of $Z(f)$, respectively. Maximum impedance amplitude ($|Z|_{\max}$, Fig. S1*E*) was defined as the maximum value of impedance across all frequencies. The frequency at which $|Z(f)|$ reached its maximum value was considered as the resonance frequency (f_{R}). Resonance strength (Q_{R}) was defined as the ratio of $|Z(f_{\mathrm{R}})|$ to $|Z(0.5)|$ (Fig. S1*E*). The impedance phase profile was computed as (Fig. S1*E*):

$$\varphi(f) = \tan^{-1}\frac{\mathrm{Im}\left(Z(f)\right)}{\mathrm{Re}\left(Z(f)\right)} \qquad (3)$$

The total inductive area, Φ_{L}, defined as the area under the inductive part (Fig. S1*E*) of $\varphi(f)$, was calculated based on the impedance phase profile (11):

$$\Phi_{\mathrm{L}} = \int_{\varphi(f)>0} \varphi(f)\, df \qquad (4)$$

Electrophysiology: Suprathreshold Measurements.

Action potential (AP) firing frequency was computed by extrapolating the number of spikes obtained during a 700 ms current injection to 1 s. The amplitude of these step-like current injections (I_{inj}) was varied from 0 pA to 250 pA in steps of 50 pA, to construct the firing frequency *vs.* injected current ($f-I$) plot (Fig. S1*H*). Several AP related measurements were derived from the voltage response of the cell, resting at V_{RMP}, to a 250 pA pulse-current injection (Fig. S1*F*–*G*). AP half-width (T_{APHW}) was the temporal width measured at the half-maximal points between the AP peak and V_{RMP} (Fig. S1*F*). The peak slope of the action potential ($\left.\frac{dV}{dt}\right|_{\mathrm{AP}}^{\max}$) was calculated from the maximum temporal derivative of the AP trace (Fig. S1*F*). The voltage

in the AP trace corresponding to the time point at which the d*V*/d*t* crossed 20 V/s defined AP threshold, V_{th} (Fig. S1*F*). AP amplitude (V_{AP}) was the maximal depolarizing deflection of the AP from V_{RMP} (Fig. S1*G*). The temporal distance between the timing of the first spike and the onset of the pulse-current injection was defined as latency to the first spike, $T_{1\mathrm{AP}}$ (Fig. S1*G*). The ratio of duration between the first and the second spikes (the first interspike interval) and last and second last spike (the last interspike interval) was defined as a measure of spike frequency adaptation, *SFA* (Fig. S1*G*).

Electrophysiology: Peri-threshold Activity Patterns.

Peri-threshold membrane potential activity was assessed in the voltage response of the cell to depolarizing pulse current injections (Fig. 1*A*) spanning 100–300 pA in steps of 20 pA, each lasting for 5 s. Due to the intrinsic heterogeneities, every cell manifested oscillations at different peri-threshold membrane potentials (Fig. 4*H*, Fig. S8). Therefore, we used this short-pulse protocol for visually identifying the range of membrane potentials where peri-threshold oscillations manifested in individual cells (Fig. 1*A*, Fig. S2). The holding voltage was then maintained around the identified range for each cell and multiple 15 s long voltage responses were recorded (Fig. S3). Voltage traces from the long-pulse protocol were subjected to quantitative criteria to find valid oscillatory traces.

Stochastic Hopf Bifurcation Simulations.

Neurons are often considered as high-dimensional nonlinear dynamical systems (12, 13). Upon receiving increasing amount of injected current, neurons typically switch from a stable resting membrane potential to a state where they manifest robust subthreshold oscillations or regular action potential firing. This property can be modeled by a nonlinear dynamical system that switches from a stable equilibrium point to manifesting limit cycles upon changes in a bifurcation parameter (injected current in the case of neurons). We employed a simple nonlinear dynamical system with such characteristics to assess the impact of stochasticity on robustness and spectral characteristics of the emergent oscillations. We specifically used the supercritical Poincare-Andronov-Hopf's bifurcation (referred to as Hopf bifurcation), a nonlinear dynamical system capable of showing bifurcation behavior resulting in stable limit cycles. The dynamics of Hopf bifurcation were governed by the following set of coupled differential equations:

$$\frac{dy_1}{dt} = \lambda y_1 - y_2 + \beta y_1(y_1^2 + y_2^2) \quad (5)$$

$$\frac{dy_2}{dt} = y_1 + \lambda y_2 + \beta y_2(y_1^2 + y_2^2) \quad (6)$$

where y_1 and y_2 were state variables that evolved temporally in a coupled manner. The dynamics followed the supercritical Hopf bifurcation if $\beta = -1$, with equilibrium at the origin ($y_1 = y_2 = 0$). λ is the bifurcation parameter, with the system showing asymptotically stable dynamics for $\lambda \leq 0$ and manifesting stable limit cycles for $\lambda > 0$. We set the integration time constant for the Hopf bifurcation simulations at 25 μs (40 kHz sampling rate) to match with the integration time constant of neuronal models.

Equations (5)–(6) define a deterministic Hopf bifurcation. We introduced either *extrinsic* (external) or *intrinsic* (parametric) noise to assess the impact of stochasticity on this dynamical system. Extrinsic noise was introduced as additive GWN:

$$\frac{dy_1}{dt} = \lambda y_1 - y_2 + \beta y_1(y_1^2 + y_2^2) + \xi_1 \quad (7)$$

$$\frac{dy_2}{dt} = y_1 + \lambda y_2 + \beta y_2(y_1^2 + y_2^2) + \xi_2 \quad (8)$$

where, ξ_1 and ξ_2 were independent, identically distributed, and were drawn at every time step from normal distributions with variance σ^2 ($\xi_i \sim \mathcal{N}(0, \sigma^2),\ i \in \{1, 2\}$). The level of noise was varied by adjusting σ^2. Intrinsic noise was introduced by introducing additive GWN to the bifurcation parameter λ that governs the emergence of stable limit cycle:

$$\lambda_\xi = \lambda + \xi \tag{9}$$

$$\frac{dy_1}{dt} = \lambda_\xi y_1 - y_2 + \beta y_1(y_1^2 + y_2^2) \tag{10}$$

$$\frac{dy_2}{dt} = y_1 + \lambda_\xi y_2 + \beta y_2(y_1^2 + y_2^2) \tag{11}$$

where ξ was drawn from a normal distribution with variance σ^2 ($\xi \sim \mathcal{N}(0, \sigma^2)$). The parameter λ_ξ was updated at each time step of the simulation, and the level of noise was varied by adjusting σ^2.

Heterogeneous population of MEC layer II stellate cell models.

We used 155 conductance-based heterogeneous population of medial entorhinal cortex (MEC) layer II stellate neuronal models constructed previously (14). The methodology for the generation of these 155 models and the measurements employed for their validation as MEC LII stellate neurons are as detailed before (14). Briefly, each neuron in this heterogeneous population was designed as a single compartment cylindrical model of length 75 μm and diameter 70 μm. The passive properties of the model were defined by a specific membrane resistance (R_m) and a specific membrane capacitance (C_m). A set of nine distinct voltage- or calcium-gated ion channel subtypes, whose gating properties and kinetics were derived from MEC LII stellate cells, controlled the active properties of these neuronal models (14). These channels were fast sodium (NaF), delayed rectifier potassium (KDR), hyperpolarization-activated cyclic-nucleotide gated (HCN) nonspecific cationic, persistent sodium (NaP), *A*-type potassium (KA), low-voltage-activated (LVA) calcium, high-voltage-activated (HVA) calcium, *M*-type potassium (KM), and small-conductance calcium-activated potassium (SK) channels. All channel kinetics were based on the Hodgkin-Huxley formulation (15), except for the SK channel, which followed six-state Markovian kinetics (14). The increase in cytosolic calcium was defined by voltage-gated calcium channel activity and decayed following a first-order kinetics with a decay time constant of τ_Ca.

We had employed a multi-parametric multi-objective stochastic search (MPMOSS) algorithm for identifying this heterogeneous population of 155 biophysically valid MEC layer II stellate cell models (14). The stochastic search was performed over 55 parameters, which governed the passive and active properties of the neurons. The validation process required that the model satisfied multiple objectives, specifically requiring ten characteristic electrophysiological measurements to fall within their respective experimental ranges: resting membrane potential (V_RMP), standard deviation of the resting membrane potential (V_SD), sag ratio (*Sag*), input resistance (R_in), resonance frequency (f_R), resonance strength (Q_R), subthreshold oscillation frequency (f_osc), number of action potential elicited for a 100 pA (N_{100}) or 400 pA (N_{400}) current injection, and amplitude of action potential (V_AP). In performing the stochastic search process, we randomly picked a value for each of 55 parameters from their respective uniform distribution to construct a single model neuron. This model was considered a "valid" neuronal model if it passed all the multiple objectives involving the 10 electrophysiological measurements. This procedure of random selection followed by validation of models was repeated 50,000 times and had yielded a heterogenous population of 155 valid models (Set I in (14)). These 155 models manifested theta-frequency subthreshold membrane potential oscillations in the theta-frequency range ($3 \leq f_\mathrm{osc} \leq 10\ \mathrm{Hz}$) as they were validated with f_osc as one of the criteria (14). These 155 valid models that satisfied all the 10 validation criteria were referred to as θ+ models (Fig. S16*A*).

Of the 50,000 models that were tested, there were a total of 623 models (including the 155 θ+ models) which satisfied 9 of the 10 characteristic electrophysiological measurements, sparing the 10th criterion on intrinsic subthreshold oscillations (f_osc). f_osc was computed as the frequency at the maximum power of the Fourier spectrum of the entire trace, for a subthreshold voltage trace near action potential threshold (14). Of these 623 deterministic models, 468 models did not manifest theta-frequency subthreshold membrane potential oscillations. We randomly picked 155 models from this set of 468 models — which satisfied 9 characteristic electrophysiological properties but did not manifest subthreshold intrinsic oscillations — for further analysis and called them as θ– models (Fig. S16*A*).

Introducing stochasticity into the heterogeneous population of MEC layer II stellate cell models.

The heterogeneous MEC LII stellate cell models were deterministic in nature, with the dynamics entirely driven by the fixed active and passive channel properties of the specific neuronal model. However, biological neurons are inherently stochastic, with two predominant forms of noise governing the function of individual neurons (16): (i) ion-channel noise, resultant from the stochastic nature of the gating properties of individual ion channels; and (ii) synaptic noise, consequent to background synaptic activity and the stochastic nature of synaptic function. We introduced ion-channel noise by making the activation gating parameter (m_{NaP}) of NaP channel in the model to be stochastic. Specifically, a zero mean GWN was added to the value of m_{NaP} at every time step and the level of noise was governed by the value of standard deviation of the GWN. Synaptic noise was introduced through a balanced high conductance state, which was introduced such that the average resting membrane potential was not altered in the presence of balanced excitatory and inhibitory synapses impinging on the model neuron. The balance was achieved by adjusting the number and frequency of activation of the different excitatory ($N_e = 100$, each with an average firing frequency of 3 Hz) and inhibitory ($N_i = 20$, each with an average firing frequency of 10 Hz) synapses. Both excitatory and inhibitory synaptic conductances were modelled as the sum of two exponentials, one defining the rise (τ_r= 2 ms) and other governing the decay (τ_d= 10 ms). The reversal potentials for excitatory and inhibitory synapses were 0 mV and –80 mV, respectively. Different levels of synaptic noise were achieved by increasing the value of scalar multipliers to the synaptic conductance values. A third form of stochasticity was introduced as extrinsic additive noise, introduced by injection of a zero mean GWN current into the model, with noise level adjusted by altering the standard deviation.

Assessment of peri-threshold oscillations in the stochastic heterogeneous population of models.

The protocol for generating peri-threshold activity traces of a given model, for assessment for the expression of membrane potential oscillations, involved step current injections ranging from 100 pA to 300 pA in steps of 10 pA, for 5 s each (21 traces generated by the protocol). This protocol was repeated for all noise levels (5 levels for each of additive and ion-channel noise and 4 levels for synaptic noise), for all three sources of noise, across each of the 155 θ+ and the 155 θ– models (21×(5+5+4)×(155+155)=91,140 traces). As noise is stochastic by definition, different instances of the same level of the same form of noise could result in very different outcomes even in the same model. Thus, there was a need to obtain multiple activity patterns for each of these different cases. We performed 10 independent trials for each model at each current injection, for each level and each form of noise (with different seed values), together yielding 911,400 stochastic activity traces (each of 5 s duration). In addition, there were 21 traces for each level of current injection from the deterministic (no noise) version of the 155 θ+ and the 155 θ– models, yielding a further 6510 traces. Each of these 917,910 traces from the θ+ and θ– models (458,955 each) were individually validated for the expression of robust oscillations using the five quantitative metrics (Fig. 4).

We used these 5 s voltage recordings for quantitative analyses of peri-threshold oscillatory activity. While using Fourier transform or Lomb's periodogram, we let the simulations to settle by removing the initial 2 s of neuron response to current injection and performed all the analyses for last 3 s of voltage traces. For comparison purposes, spectral analyses of traces from Hopf bifurcation simulations were also performed over the last 3 s of the time series. We used wavelet transform to perform time-frequency analysis of *unfiltered* outputs from the Hopf bifurcation simulations, from the neuronal models, from electrophysiological recordings, and from the model involving a generalized network motif.

Identification of distinct types of peri-threshold activity in valid oscillatory traces.

We identified three distinct types of peri-threshold activity patterns among valid oscillatory traces (satisfying all validation criteria from Fig. 4*A*–*E*). This classification was based on the ratio (f_{AP}/f_{osc}) between f_{AP}, the action-potential firing rate of the neuron during the recorded period and f_{osc}, the oscillation frequency within the theta-frequency range (Fig. S8; Fig. S20*A*–*B*). The first type of traces showed valid oscillations but did not fire any action potentials ($f_{AP} = f_{AP}/f_{osc} = 0$). Thus, this group of traces manifested purely sub-threshold oscillations with no spiking during the period. The second group contained traces where the action potential firing rate was lesser than the oscillatory frequency ($0 < f_{AP}/f_{osc} < 0.8$). The

third group contained traces where the action potential firing rate was comparable to the oscillatory frequency ($f_{\mathrm{AP}}/f_{\mathrm{osc}} \geq 0.8$). The threshold value of 0.8 was arrived from the trimodal nature of the histogram of $f_{\mathrm{AP}}/f_{\mathrm{osc}}$ across different models and noise levels (Fig. S9, Fig. S20*C*–*D*). Although the third group would imply the presence of spikes in every cycle of the sub-threshold oscillations, we noted that these traces manifested several cycles of the subthreshold oscillations where there were no spikes and others with doublets/bursts of action potentials (Fig. S8, Fig. S20*A*–*B*). Therefore, the second and third type of traces showed mixed-mode oscillations where spikes and sub-threshold oscillations coexisted, with spikes riding atop the sub-threshold oscillations (Fig. S8, Fig. S20*A*–*B*). We also computed the fraction of valid oscillatory traces, across pooled traces (Fig. S10, Fig. S21*A*–*C*) or within individual valid models (Fig. S11, S21*D*), belonging to each of these three groups.

Virtual knockout of individual ion channels in LII MEC stellate cell models.

The impact of removing individual ion channels on the different electrophysiological measurements have been assessed earlier (14) using the virtual knockout model (VKM) approach (17). Here, we assessed the impact of virtually removing different ion channels on the number of valid oscillatory traces at different levels of noise, employing the five metrics (Fig. 4) that have been developed to assess valid peri-threshold oscillatory traces. We performed these analyses on the θ+ ($n_{\theta+}$ = 155) model neuron population. In performing these VKM simulations, we first set the conductance values of each of the 9 active ion channels independently to zero in each of the 155 θ+ model neurons. For each of the 9 different channel knockouts, we generated activity traces at 21 different current injection values and 6 distinct levels of additive noise, with each pair repeated for 10 independent trials. We then validated the oscillatory activity for each instance based on the spectrogram-based custom measurements and assessed the sensitivity of stable oscillatory activity to different channels (Fig. 7*A*).

Heterogeneous population of LII MEC stellate cell models with stochastically gated ion channels: Model specification.

We used PSICS- the parallel stochastic ion channel simulator (http://www.psics.org/) for building a LII MEC stellate cell model with stochastically gated ion channels (18), with an integration time constant of 50 µs. We employed a single compartmental spherical model of 43.87 µm radius (Fig. 7*B*), which was adjusted to obtain the same surface area as the cylindrical LII MEC SC model with deterministic ion channels. The passive properties were incorporated into the model as an RC circuit that was defined through a stochastically gated leak ion channel conductance, $g_{leak} = 1/R_{\mathrm{m}}$, where R_{m} specified the specific membrane resistance and a specific membrane capacitance, C_{m}, was set at a default value of 1 µF/cm^2.

We incorporated 6 different active stochastically gated ion channels into the model (Fig. 7*B*): fast sodium (NaF), delayed rectifier potassium (KDR), hyperpolarization-activated cyclic-nucleotide gated (HCN) nonspecific cationic, persistent sodium (NaP), *A*-type potassium (KA), and *M*-type potassium (KM) channels (Fig. 7*B*). The kinetics and gating properties of these stochastically gated channels were matched to their respective deterministic counterparts used in this study (14). Each inactivating stochastic ion channel subtype was modeled with both activation (m) and inactivation (h) gating variables, whereas non-inactivating stochastic ion channels were endowed with a single activation gating variable (m). Each gating variable was modeled with a two-state model representing closed (C) or open (O) states. The kinetics of the transitions from C-to-O and O-to-C for each gating variable were governed by voltage-dependent rates α and β, respectively (19). The kinetic model for g_{leak} was adopted from (19) with a forward reaction rate of 4 s^{-1} and reverse reaction rate of 1 s^{-1}. The single leak channel conductance was 20 pS. The rates associated with the gating particles for each of the different active channels are listed below.

Fast sodium channel: The NaF model was adopted from (19), and the single channel conductance was 15 pS.

The activation gating (m^{NaF}) kinetics was defined by:

$$\alpha_m^{NaF} = \frac{4\big((V+33)/9\big)}{\big(1-\exp(-(V+33)/9)\big)} \qquad \beta_m^{NaF} = \frac{27.6\big((V+58)/-12\big)}{\Big(1-\exp\big((V+58)/12\big)\Big)}$$

The inactivation gating (h^{NaF}) kinetics was defined by:

$$\alpha_h^{NaF} = \frac{0.36\big((V+48)/-12\big)}{\Big(1-\exp\big((V+48)/12\big)\Big)} \qquad \beta_h^{NaF} = \frac{0.4\big((V+11)/6\big)}{\big(1-\exp(-(V+11)/6)\big)}$$

There were three instances of m^{NaF} and one instance of h^{NaF}.

Delayed rectifier potassium channel: The KDR model was adopted from (19), and the single channel conductance was 20 pS.

The activation gating particle (m^{KDR}) kinetics was defined by:

$$\alpha_m^{KDR} = \frac{0.2\big((V+38)/10\big)}{\big(1-\exp(-(V+38)/10)\big)} \qquad \beta_m^{KDR} = \frac{0.6294\big((V+47)/-35\big)}{\Big(1-\exp\big((V+47)/35\big)\Big)}$$

There were four instances of m^{KDR}.

Hyperpolarization-activated cyclic-nucleotide gated nonspecific cationic channel: The HCN model was adopted from (19), and the single channel conductance was 1 pS.

The activation gating particle (m^{HCN}) kinetics was defined by:

$$\alpha_m^{HCN} = \frac{0.0183}{1+\exp\left(\frac{V+114.2}{20.33}\right)} \qquad \beta_m^{HCN} = \frac{0.033}{1+\exp\left(\frac{-(V+51.5)}{10.94}\right)}$$

There was one instance of m^{HCN}.

Persistent sodium channel: The NaP model was adopted from (19), and the single channel conductance was 10 pS.

The activation gating particle (m^{NaP}) kinetics was defined by:

$$\alpha_m^{NaP} = \frac{8.821}{1+\exp\left(\frac{-(V-10)}{20.75}\right)} \qquad \beta_m^{NaP} = \frac{3.855}{1+\exp\left(\frac{(V+69.92)}{11.4}\right)}$$

The inactivation gating particle (h^{NaP}) kinetics was defined by:

$$\alpha_h^{NaP} = \frac{0.00016\big((V+64.409)/2.63\big)}{\big(1-\exp(-(V+64.409)/2.63)\big)} \qquad \beta_h^{NaP} = \frac{0.00012\big((V+17.014)/-4.63\big)}{\Big(1-\exp\big((V+17.014)/4.63\big)\Big)}$$

There was one instance of m^{NaP} and one instance of h^{NaP}.

***A*-type potassium channel:** The KA model was adopted from (19), and the single channel conductance was 6 pS.

The activation gating particle (m^{KA}) kinetics was defined by:

$$\alpha_m^{KA} = \frac{0.15\big((V+18.3)/15\big)}{\big(1-\exp(-(V+18.3)/15)\big)} \qquad \beta_m^{KA} = \frac{0.15\big((V+18.3)/-15\big)}{\Big(1-\exp\big((V+18.3)/15\big)\Big)}$$

The inactivation gating particle (h^{KA}) kinetics was defined by:

$$\alpha_h^{KA} = \frac{0.082\big((V+58)/{-8.2}\big)}{\big(1-\exp\big((V+58)/8.2\big)\big)} \qquad \beta_h^{KA} = \frac{0.082\big((V+58)/8.2\big)}{\big(1-\exp(-(V+58)/8.2)\big)}$$

There was one instance of m^{KA} and one instance of h^{KA}.

***M*-type potassium channel:** The KM model was adopted from (20), and the single channel conductance was 10 pS. The activation gating particle (m^{KM}) kinetics was defined by:

$$\alpha_m^{KM} = \frac{0.01675}{1+\exp\left(\frac{-(V+32.87)}{9.046}\right)} \qquad \beta_m^{KM} = \frac{0.01756}{1+\exp\left(\frac{(V+52.35)}{13.23}\right)}$$

There was one instance of m^{KM}.

Heterogeneous population of LII MEC stellate cell models with stochastically gated ion channels: Unbiased stochastic search and electrophysiological validation.

A single hand-tuned model manifests a specific form of oscillatory pattern mediated by one set of ion channels and would not be representative of the biophysical heterogeneities in SCs. Interpretations from a single hand-tuned model would thus be biased by this specific choice of parameters that were introduced in that single model (14, 21, 22). To avoid such biased conclusions, we generated a population of stochastically gated SC models that matched characteristic electophysiological properties of stellate cells. To generate intrinsically heterogeneous population of LII MEC stellate cells with stochastically gated ion channels, we employed the multi-parametric multi-objective stochastic search (MPMOSS) algorithm employed earlier to generate the heterogenous population of SC models with deterministic ion-channel models (14). In this independent implementation of MPMOSS performed on models with stochastically gated ion channels, we performed a randomized search on the 7 channel conductances (the bounds on each search parameter are given in Table S3) to generate 10,000 randomized models (Fig. 7*C*). We validated these randomly generated models against an array of 7 signature electrophysiological measurements of SC calls (Table S4). Of the 10,000 models, we found 90 valid models (0.9%) that satisfied all these 7 electrophysiological bounds (Fig. 7*D*).

The measurement protocols for each of the 7 different validation measurements (Table S4) were similar to protocols in (14), and were adjusted to account for the stochastic nature of these models. Specifically, for each valid stochastic stellate cell model, each intrinsic measurement was computed as an average of 10 independent trials. The single-trial protocol for each measurement is provided below. The average resting membrane potential (V_{RMP}) was measured as the mean voltage value recorded over 50 ms in the absence of any current injection. *Sag* was measured as the ratio of steady-state deflection to the peak deflection in membrane potential for a hyperpolarizing pulse of 100 pA for 1000 ms. Input resistance (R_{in}) was computed as the slope on the *V–I* plot containing the steady-state voltage values for hyperpolarizing and depolarizing pulses of 100 pA for 1000 ms, plotted against the respective current value. The number of action potentials at 100 pA (N_{100}) and 400 pA (N_{400}) were obtained from a 500 ms step current injection. We ensured that N_{100} was zero for all valid models across for each of the 10 trials. The spike amplitude (V_{AP}) was computed as the peak of first action potential (from V_{RMP}) in response to a 400 pA current injection. We measured al intrinsic measurements after allowing the membrane potential to settle at a steady-state potential when no current was injected for a period of 5 s (14). The passive membrane potential, in the absence of any active conductance, was set to be –77 mV (14). During the initial 5 s period, the interplay between the different active subthreshold conductances leads to membrane potential fluctuation and eventually settling to V_{RMP}.

Peri-threshold oscillations in valid LII stochastic stellate models were assessed using the same protocols as those for their deterministic counterparts. Specifically, after the initial 5 s delay period, we recorded the voltage response of the

model to depolarizing pulse current injections spanning 100–300 pA in steps of 10 pA, each lasting for 5 s (21 traces generated by the protocol for each model for every trial). The same protocol was used to obtain peri-threshold activity across 6 different levels of additive noise for 10 independent trials. It is important to note that ion-channel noise (consequent to stochastic gating of all ion channels) was always present in these stochastic SC models, irrespective of the level of additive noise (including the zero-noise condition) introduced. The spectrogram-based custom-made quantitative measurements developed in this study were used to assess validity of oscillatory traces.

A generalized network motif for intrinsic stochastic oscillatory activity in neurons: Coupled slow negative and fast positive feedback loops with noise.

In order to generate input-dependent oscillatory activity with simple integrator dynamics (mimicking the parallel RC circuit of neurons), the addition of a slow negative feedback loop motif (mimicking resonating conductances like HCN or KM channels) coupled to a fast positive feedback loop motif (mimicking amplifying conductances like persistent sodium channels) is required (Fig. 8*A*). The dynamics of such a simplified stochastic oscillatory model system was simulated as the following system of coupled differential equations:

$$\tau \frac{dS}{dt} = -S - g\, m(S) + h\, n(S) + I + \xi \tag{12}$$

$$\frac{dm}{dt} = \frac{m_\infty - m}{\tau_m} \tag{13}$$

$$\frac{dn}{dt} = \frac{n_\infty - n}{\tau_n} \tag{14}$$

where, S defined model activity, τ was the integration time constant (default value of 10 ms) of the RC circuit, I represented the input to the model and the bifurcation parameter for manifestation of oscillations, and ξ defined additive noise to the model system. ξ was drawn from a normal distribution with variance σ^2 ($\xi \sim \mathcal{N}(0, \sigma^2)$) at every time step of the simulation. The level of noise was varied by adjusting σ^2. m and n defined the positive and negative feedback state variables, respectively. g (default value 0.1) and h (default value 0.1) represented feedback strength for m and n state variables, respectively. The slow kinetics of the negative feedback was controlled by the feedback time constant (τ_{m}) with a default value of 75 ms. The fast kinetics of the positive feedback was controlled by the feedback time constant (τ_{n}) with a default value of 5 ms. The steady-state feedback kernel (m_∞) of the negative feedback loop was set to be sigmoidally dependent on the output of the model (S), with default value of the half-maximal activity ($S_{1/2}^m$) to be 0.15 and the slope (k^m) to be 0.1:

$$m_\infty = \left(1 + \exp\left(\frac{S_{1/2}^m - S}{k^m}\right)\right)^{-1} \tag{15}$$

The steady-state feedback kernel (n_∞) of the positive feedback loop was set to be sigmoidally dependent on the output of the model (S), with default value of the half-maximal activity ($S_{1/2}^n$) to be 0.18 and the slope (k^n) to be 0.1:

$$n_\infty = \left(1 + \exp\left(\frac{S_{1/2}^n - S}{k^n}\right)\right)^{-1} \tag{16}$$

Computational details.

All simulations involving conductance-based stellate cell models with deterministic ion channels models were performed using the NEURON programming environment (23) at 34 ℃, with a simulation step size of 25 µs. We used PSICS-the parallel stochastic ion channel simulator (http://www.psics.org/) for simulating conductance-based stellate cell models with stochastically gated ion channels (18), at 34 ℃, with a simulation step size of 50 µs. All Hopf bifurcation simulations, the generation of theta-filtered noise traces, and the simulations associated with the generalized network motif for stochastic intrinsic oscillations were performed in MATLAB 2018a (Mathworks). All data analyses and plotting were implemented using custom-written scripts on MATLAB 2018a (Mathworks) or IGOR Pro (WaveMetrics) environments. All statistical analyses were performed using the R computing and statistical package (R core Team 2013).

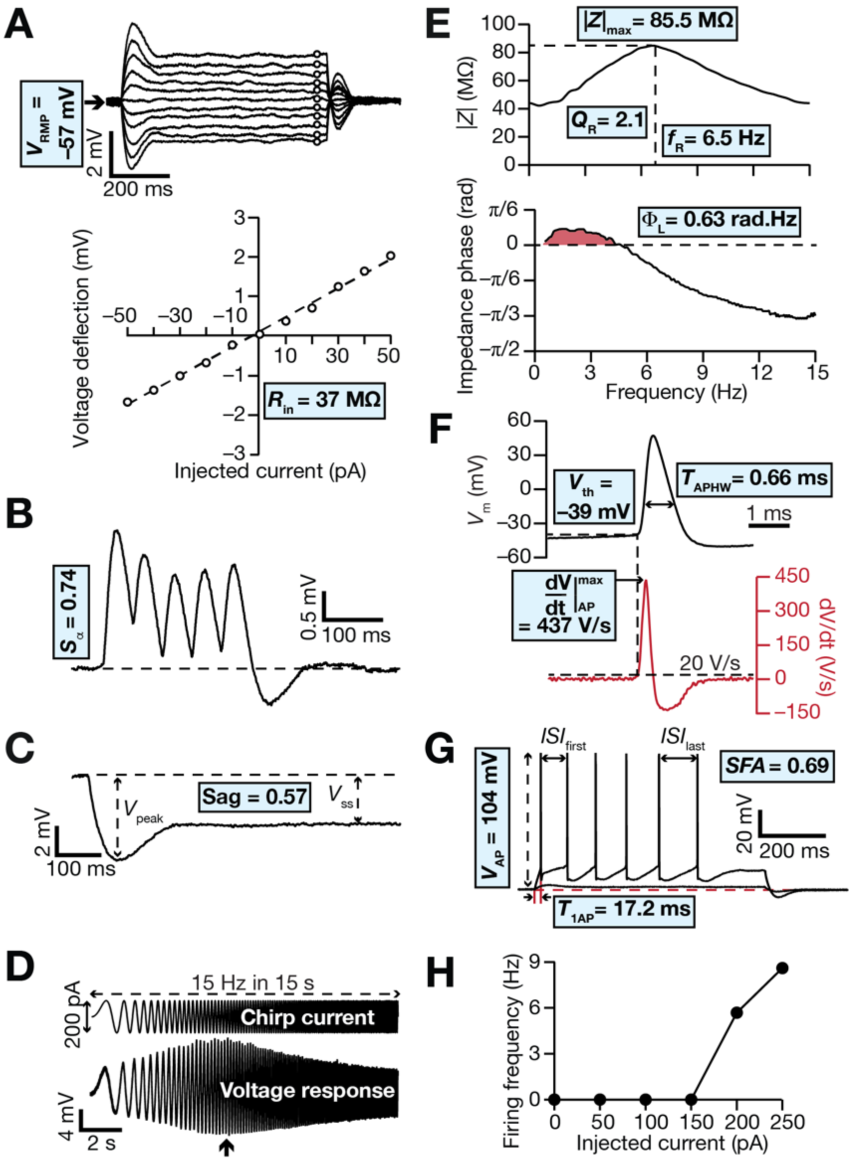

Supplementary Figure S1: Illustration of characteristic subthreshold and suprathreshold measurements of rat MEC layer II stellate cells recorded using whole-cell patch clamp electrophysiology. (A–H) Examples of electrophysiological recordings from a stellate cell, depicting the different measurements. (A) Resting membrane potential, V_{RMP}; input resistance, R_{in}; (B) Temporal summation ratio, S_{α}; (C) Sag ratio, *Sag*; (D–E) Resonance frequency, f_{R}; resonance strength, Q_{R}; total inductive area, Φ_{L} (F) Action potential (AP) half-width, T_{APHW}; AP maximum slope, $\left.\frac{dV}{dt}\right|_{\mathrm{AP}}^{\max}$; AP threshold, V_{th} (G) AP amplitude, V_{AP}; spike frequency adaptation, *SFA*; latency to first AP, $T_{1\mathrm{AP}}$. (H) AP firing frequency for different current injections.

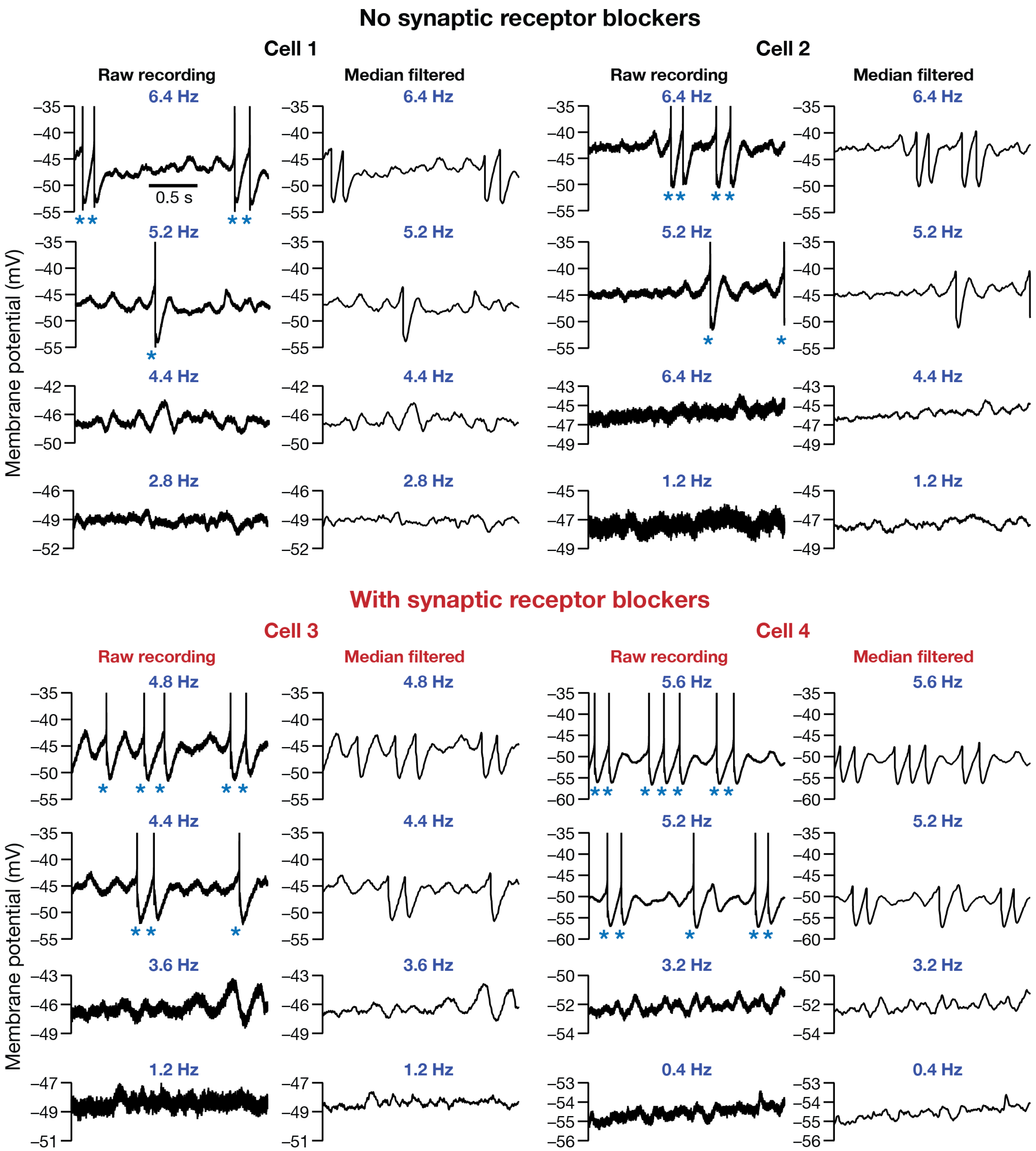

Supplementary Figure S2: Example voltage traces from electrophysiologically recorded LII MEC stellate cells using the short-pulse protocol. Each column depicts four sets of traces recorded at different voltage levels from a single neuron. For each cell, the raw recordings (*Left*) along with the median filtered version of same trace (*Right*) are shown. Cells 1–2 were recorded in the absence of synaptic blockers (black), whereas Cells 3–4 were recorded in the presence of synaptic blockers (red). Note that when spikes occurred (blue asterisks), they were truncated to –35 mV for emphasizing the subthreshold dynamics.

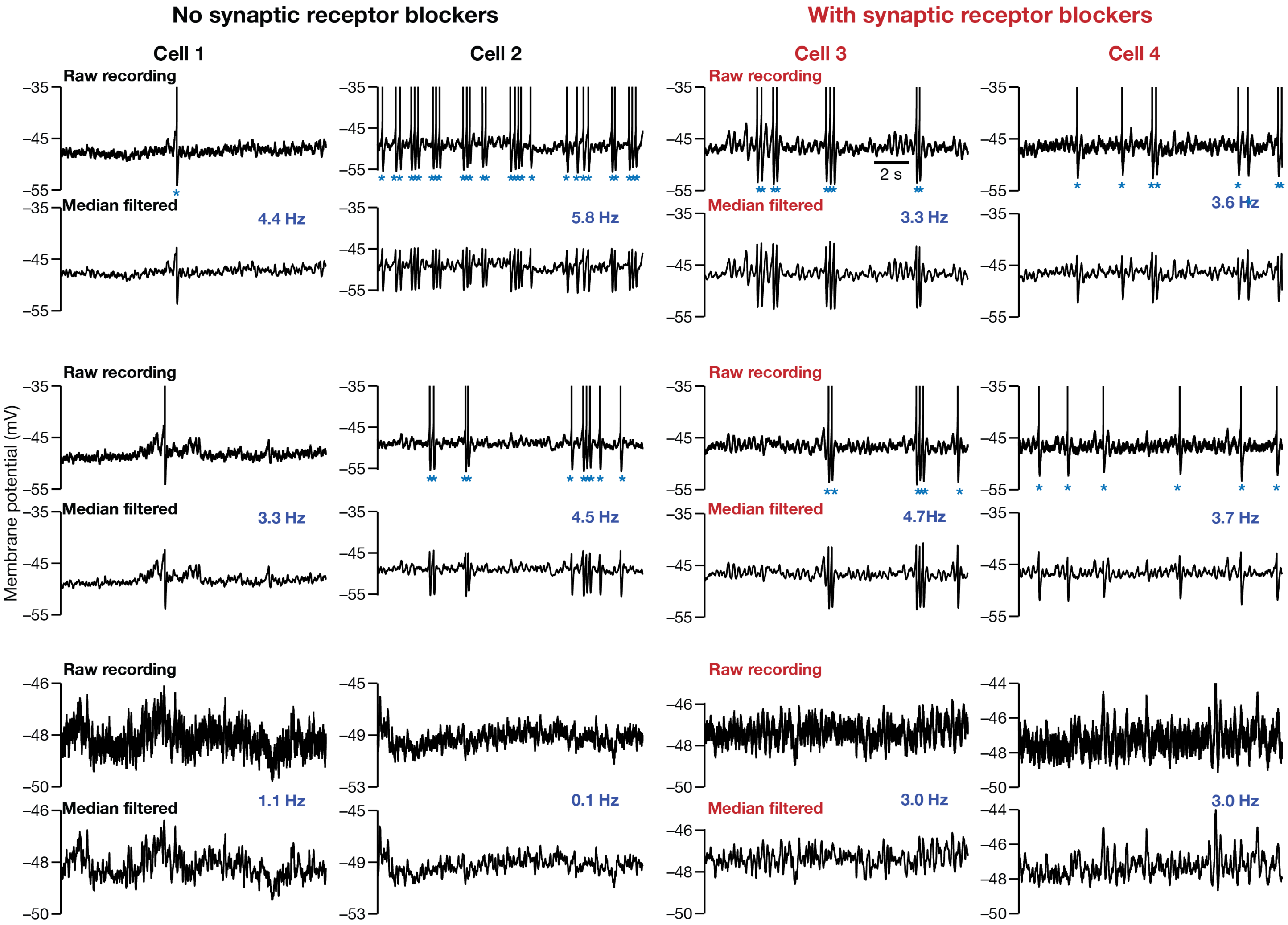

Supplementary Figure S3: Example voltage traces from electrophysiologically recorded LII MEC stellate cells using the long-pulse protocol. Each column depicts three sets of traces recorded at different voltage levels from a single neuron. For each cell, the raw recordings (*Top*) along with the median filtered version of same trace (*Bottom*) are shown. Cells 1–2 were recorded in the absence of synaptic blockers (black), whereas Cells 3–4 were recorded in the presence of synaptic blockers (red). Note that when spikes occurred (blue asterisks), they were truncated to –35 mV to emphasize subthreshold dynamics. Note the clustered patterns of spiking observed in these recordings, where a role for afterhyperpolarization and associated activation of HCN channels has been demonstrated in previous studies (19, 24, 25).

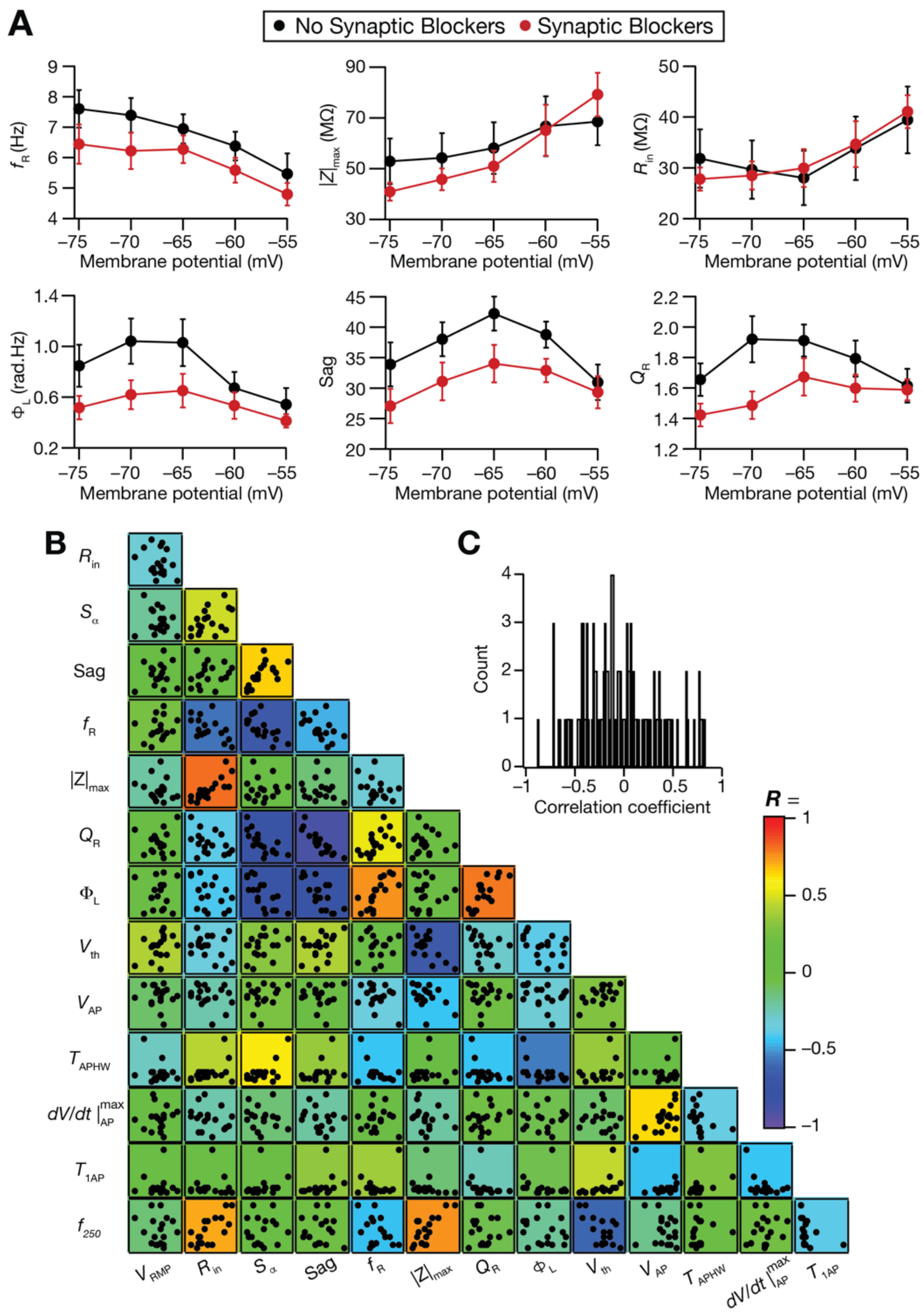

Supplementary Figure S4: Voltage-dependent properties of sub-threshold measurements and pairwise correlations across sub and supra-threshold measurements for electrophysiologically recorded LII MEC stellate cells. (A) Voltage dependence of sub-threshold response properties of stellate cells recorded with (red) or without (black) synaptic receptor blockers. Six sub-threshold measurements for each recorded cell were recorded by holding the cell at different voltages spanning –75 mV to –55 mV in steps of 5 mV. (B) Lower diagonal matrix depicting the pairwise scatter plot between 14 sub- and supra-threshold measurements from 28 LII MEC stellate cells (13 cells with and 15 without synaptic blockers). Individual scatterplots are overlaid on a heat map that depicts the pairwise correlation coefficient computed for that scatter plot. Inset: distribution of the correlation coefficient values from scatter plots in B. While most measurements showed weak pairwise correlations, some of measurements that showed relatively strong correlations are known to be dependent on the same sets of ion channels (14).

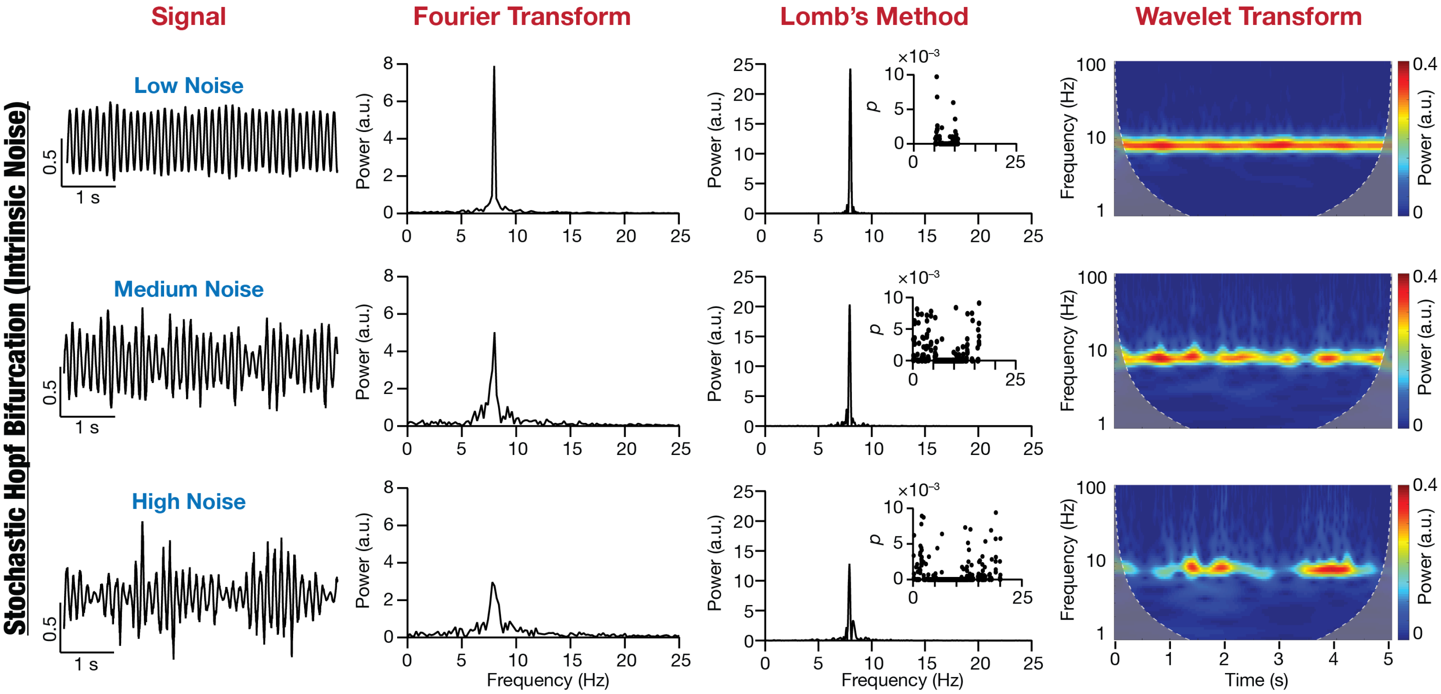

Supplementary Figure S5: Spectral properties of oscillations emergent from a stochastic non-linear dynamical system with intrinsic noise. Impact of parametric (intrinsic) noise on oscillations emerging from a nonlinear dynamical system (Hopf bifurcation). *Row 1*: Low noise, *Row 2*: Medium noise, and *Row 3*: High noise. *Column 1*: time-domain signal, *Column 2*: Fourier Transform (notice the 8 Hz peak) of the signal, *Column 3*: Lomb's periodogram of the signal with inset depicting the significance of each peak in the periodogram, and *Column 4*: spectrogram of the signal computed using wavelet transform.

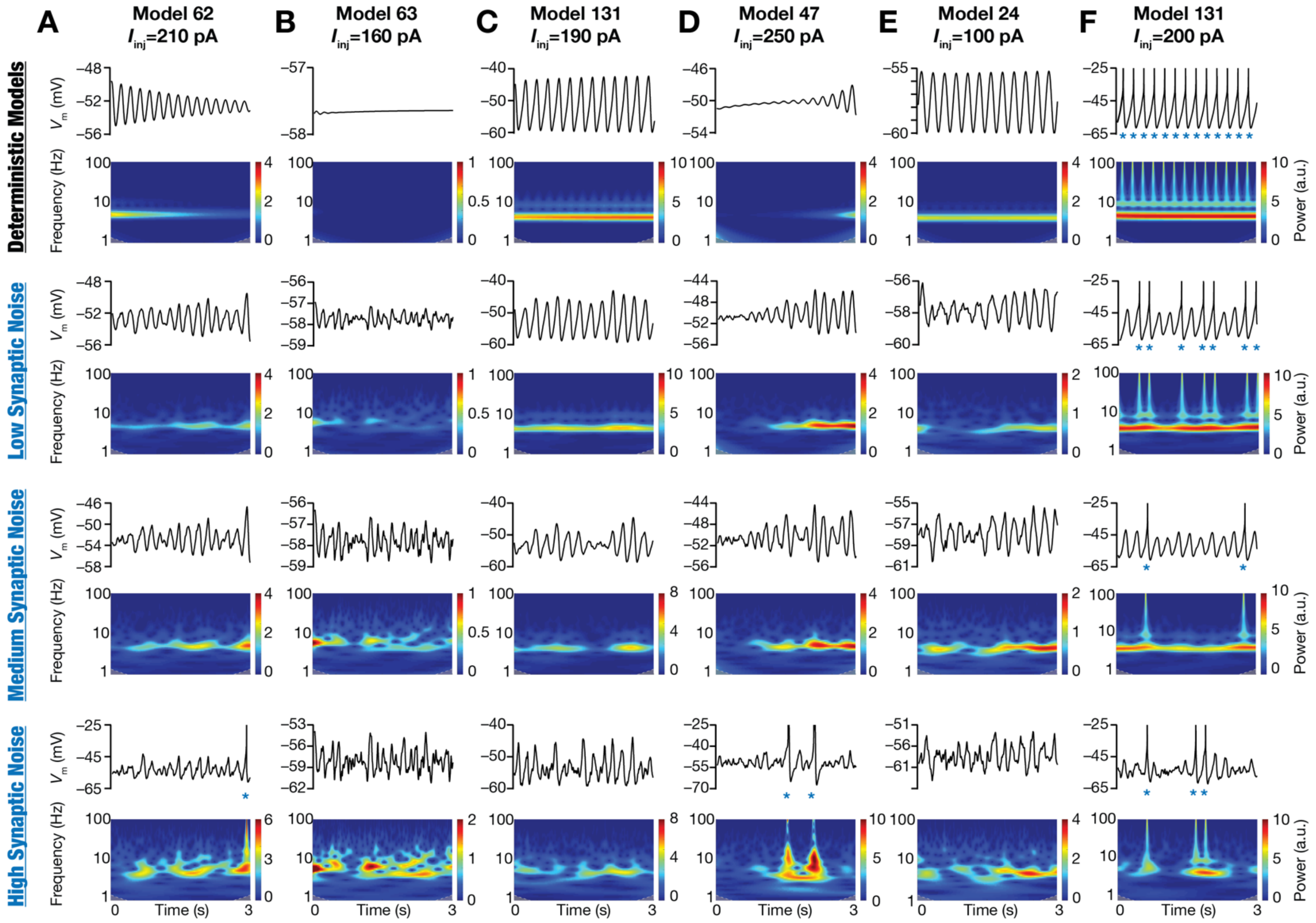

Supplementary Figure S6: Illustrative examples of the role of synaptic noise in stabilizing peri-threshold oscillatory patterns in a heterogeneous population of LII MEC stellate cell models. Each row in panels (A–F) depicts intrinsic activity patterns from different models for a 3 s period and the corresponding spectrograms computed using wavelet transform. Each column is identified by the corresponding model number along with identified values of injected current (I_{inj}) employed to generate the activity patterns. The first row depicts activity patterns in the deterministic model, in the absence of any form of noise. Rows 2–4 depict activity patterns from the same model, injected with the same I_{inj} value, with low, medium, and high levels of synaptic noise. Note that when spikes occurred (blue asterisks), they were truncated to –25 mV to emphasize subthreshold dynamics.

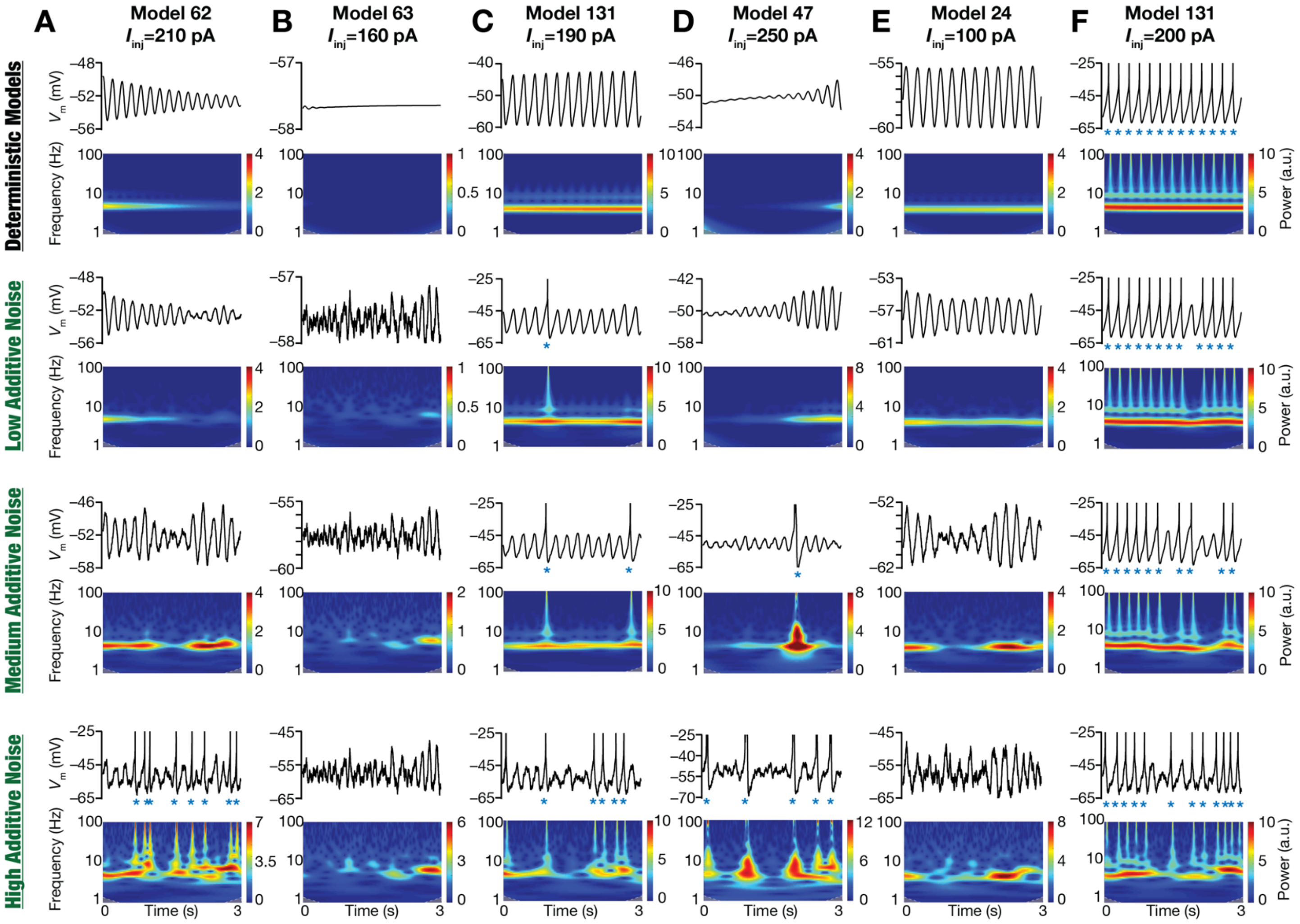

Supplementary Figure S7: Illustrative examples of the role of external additive noise in stabilizing peri-threshold oscillatory patterns in a heterogeneous population of LII MEC stellate cell models. Each row in panels (A–F) depicts intrinsic activity patterns from different models for a 3 s period and the corresponding spectrograms computed using wavelet transform. Each column is identified by the corresponding model number along with identified values of injected current (I_{inj}) employed to generate the activity patterns. The first row depicts activity patterns in the deterministic model, in the absence of any form of noise. Rows 2–4 depict activity patterns from the same model, injected with the same I_{inj} value, with low (0.03), medium (0.12), and high (0.48) levels of additive noise. Note that when spikes occurred (blue asterisks), they were truncated to –25 mV to emphasize subthreshold dynamics.

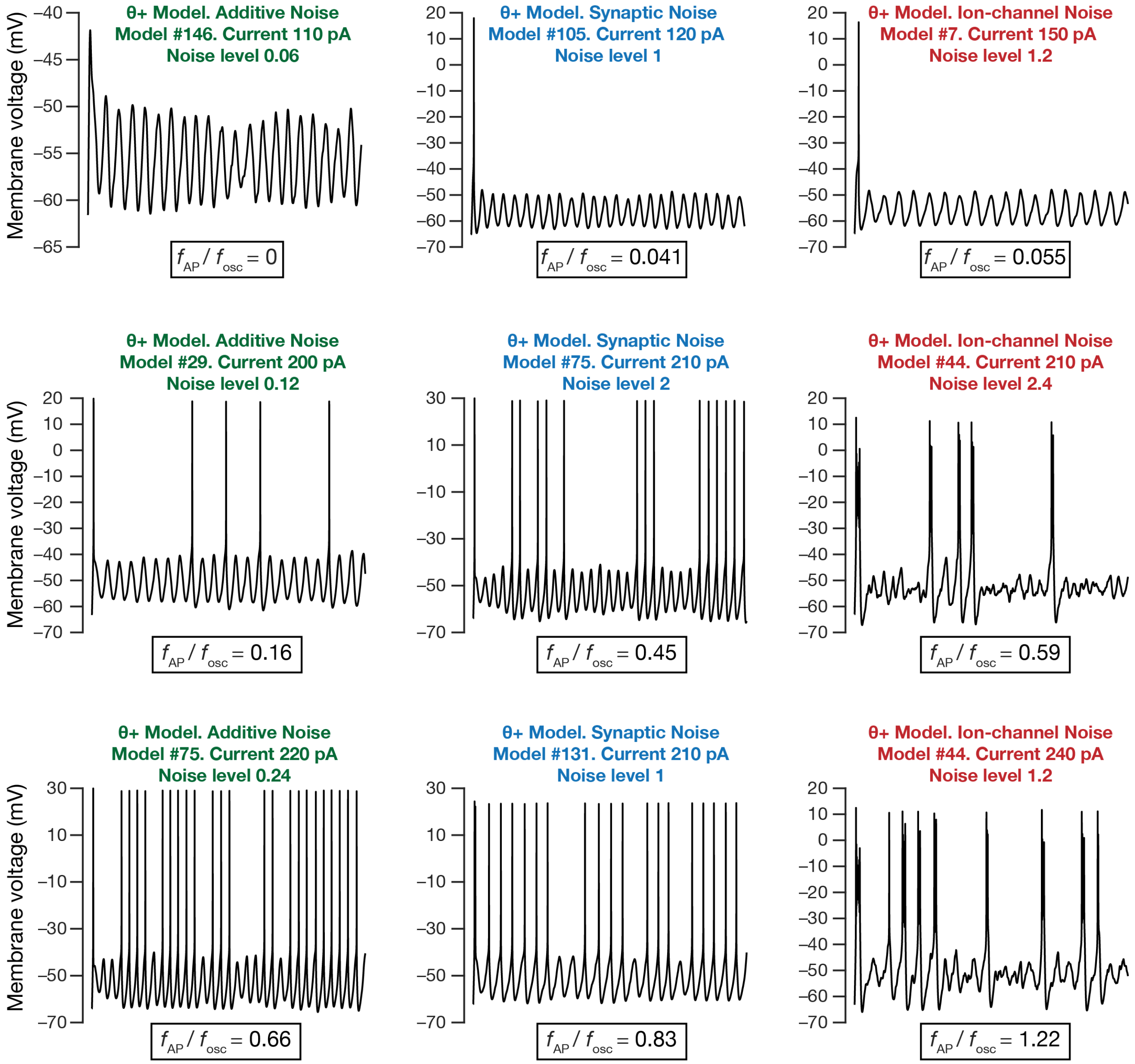

Supplementary Figure S8: Example traces of valid oscillatory patterns with different values of the ratio between the action potential firing rate and the oscillatory frequency. f_{AP} represents action potential firing rate and f_{osc} denotes oscillation frequency of the valid oscillatory trace. The example traces were taken from θ+ model neurons with different forms and levels of noise and varied values of the injected current, the bifurcation parameter. The traces show the continuum of traces from purely subthreshold traces ($f_{\mathrm{AP}}/f_{\mathrm{osc}} = 0$) to mixed mode oscillations where spikes or bursts ride atop peri-threshold oscillatory patterns. It may be noted that even under scenarios where the value of $f_{\mathrm{AP}}/f_{\mathrm{osc}}$ was greater than 1, there were theta oscillatory cycles where there were no spikes. In these cases, the ratio was greater than 1 because of bursts in some or all cycles. There were several valid oscillatory traces across models (with different noise configurations and current values) where the rest of the trace manifested sub-threshold oscillations, with merely a single spike at the beginning where the current pulse was initiated. All traces are for a period of 5 s. It may be noted that the presence of spike(s) enhanced the amplitude of the oscillation in that cycle owing the large afterhyperpolarization (AHP) and associated rebound. The role of spike-associated AHP and AHP-associated post-inhibitory rebound in amplifying peri-threshold oscillations and in mediating spike clustering is limited to the cycles where spikes are observed. The ability of the rebound to translate to another spike translated to spike clustering in traces (19, 24, 25).

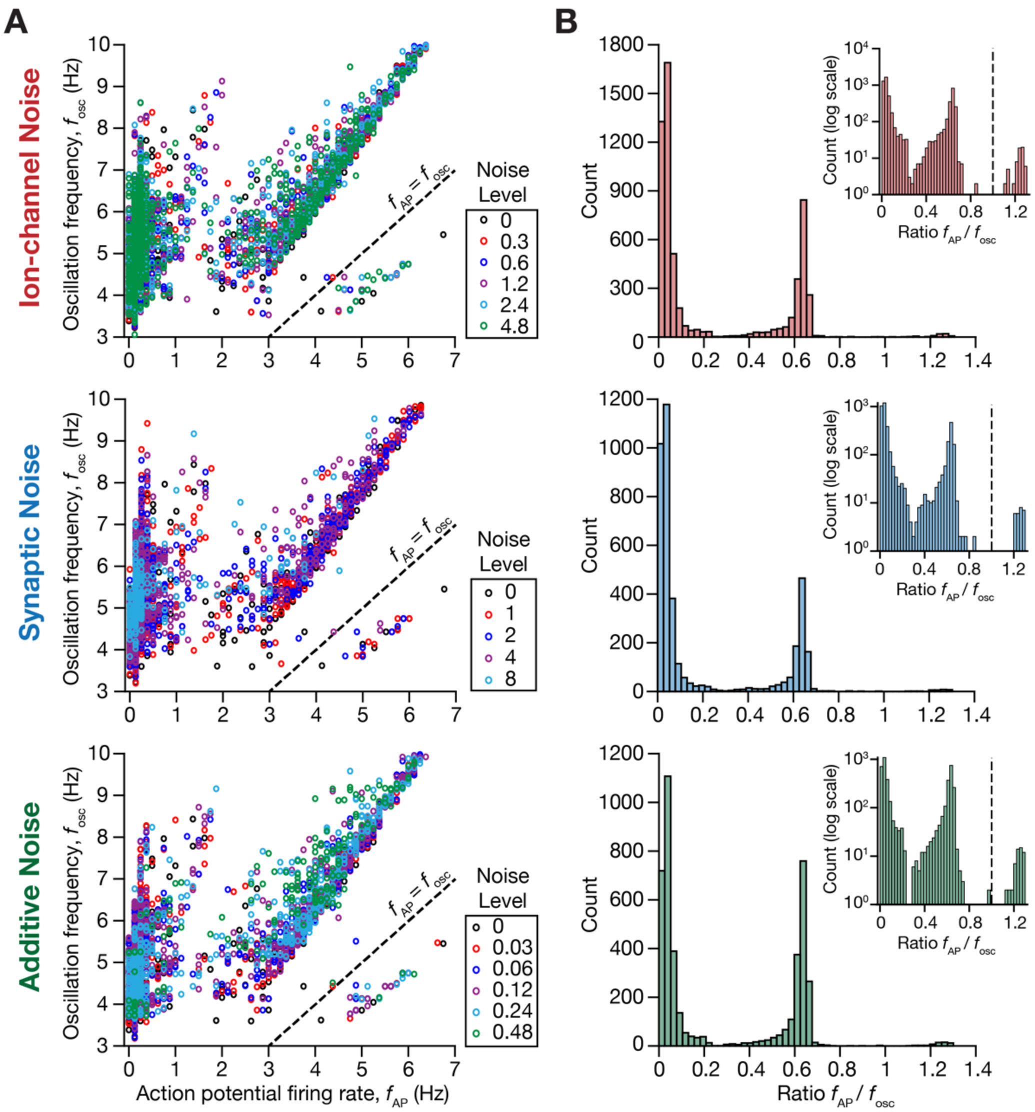

Supplementary Figure S9: The relationship between action potential firing rate and oscillation frequency distinguished different peri-threshold activity patterns with different forms of noise. (A) Oscillation frequency is plotted against action potential firing rate for each valid voltage trace. The dashed line represents the $f_{\mathrm{AP}}/f_{\mathrm{osc}} = 1$ line. f_{AP}: action potential firing rate. f_{osc}: oscillation frequency of the valid oscillatory trace. In each plot, different colors represent different noise levels. (B) Histogram of ratio between action potential firing rate f_{AP} and oscillation frequency f_{osc} across all valid oscillatory traces. Inset: histogram of $f_{\mathrm{AP}}/f_{\mathrm{osc}}$ in log scale. The dashed line represents the $f_{\mathrm{AP}}/f_{\mathrm{osc}} = 1$ line. The trimodal nature of the histogram may be noted. A large proportion of traces showed no spikes or very few spikes (proportion of traces with no spikes ($f_{\mathrm{AP}}/f_{\mathrm{osc}} = 0$): ion-channel noise: 4.99%, synaptic noise: 8.04%, additive noise: 6.11%; proportion of traces with $0 < f_{\mathrm{AP}}/f_{\mathrm{osc}} < 0.1$ where there are very few spikes ($f_{\mathrm{AP}} \leq 1$ Hz): ion-channel noise: 57.7%, synaptic noise: 59.7%, additive noise: 47.2%). The second mode contained traces where $f_{\mathrm{AP}}/f_{\mathrm{osc}}$ centered around 0.6 (proportion of traces with $0.5 \leq f_{\mathrm{AP}}/f_{\mathrm{osc}} \leq 0.7$: ion-channel noise: 29.5%, synaptic noise: 24.5%, additive noise: 38.0%), where the firing frequency was still less than the oscillation frequency. A very small proportion of traces had $f_{\mathrm{AP}}/f_{\mathrm{osc}} \geq 1$ owing to bursting behavior in some cycles (proportion of traces with $f_{\mathrm{AP}}/f_{\mathrm{osc}} \geq 1$: ion-channel noise: 1.1%, synaptic noise: 0.76%, additive noise: 1.26%). Together, valid oscillatory traces showed pure sub-threshold oscillations or mixed-mode oscillatory patterns where sub-threshold oscillations and spikes coexisted, with spikes skipping oscillatory cycles in these traces even in scenarios where $f_{\mathrm{AP}}/f_{\mathrm{osc}} > 1$ (examples with different $f_{\mathrm{AP}}/f_{\mathrm{osc}}$ are in Fig. S8). These analyses showed that spikes were sparse in most valid oscillatory traces showing mixed mode oscillations. These analyses were performed for valid traces from the 155 θ+ model neurons (Fig. 5*A*–*C*). All analyses were performed on the entire 5 s duration of the recorded traces.

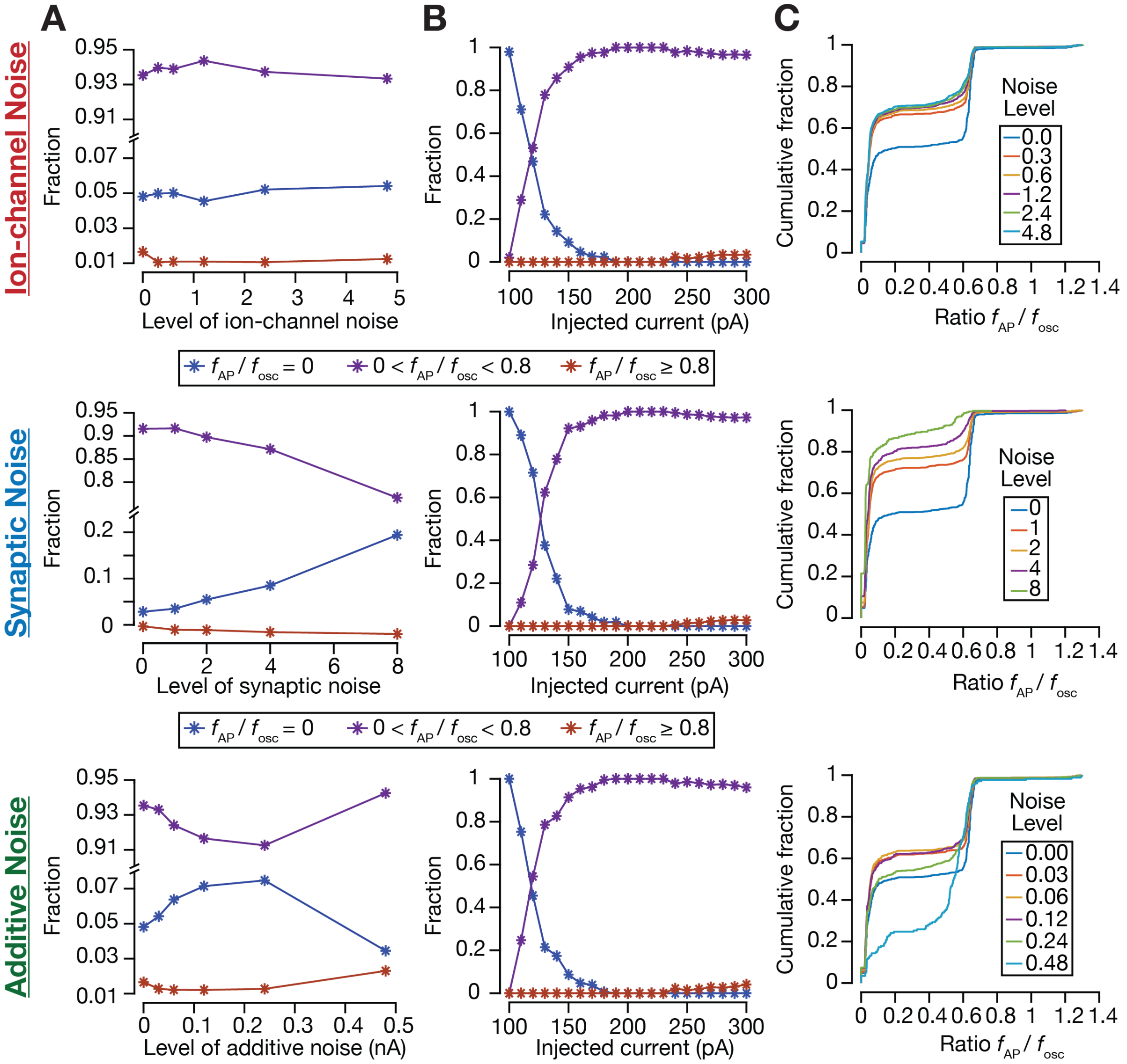

Supplementary Figure S10: Proportions of distinct types of peri-threshold oscillatory activity. (A–B) Valid oscillatory traces were divided into three groups based on the relationship between action potential firing rate $f_{\rm AP}$ and oscillation frequency $f_{\rm osc}$. Group 1 (blue): No spikes, only subthreshold oscillations ($f_{\rm AP}/f_{\rm osc} = 0$). Group 2 (purple): Rate of spikes less than oscillation frequency ($0 < f_{\rm AP}/f_{\rm osc} < 0.8$). Group 3 (red): Rate of spikes comparable to oscillation frequency ($f_{\rm AP}/f_{\rm osc} \geq 0.8$). The fraction of traces that belonged to each of these three groups were plotted for different types of noise as functions of level of noise (A) or the injected current, the bifurcation parameter (B). Across different types of noise, most traces belonged to the second group where the rate of spikes was less than the oscillation frequency ($0 < f_{\rm AP}/f_{\rm osc} < 0.8$), with a small fraction of traces falling into the first or the third groups (*A*). As a function of injected current, expectedly more valid Group 1 (sub-threshold oscillations) traces were present for lower current injections, switching to Group 2 with increasing values of current injections (*B*). Plots in panels A and B contain pooled valid traces for all current injection values and all noise levels, respectively. (C) Cumulative distribution of $f_{\rm AP}/f_{\rm osc}$ plotted for different levels of different forms of noise. Across different models and forms of noise, it may be noted that a vast majority of traces fall into either Group 1 or Group 2, indicating that the AP firing rate in these valid oscillatory traces was less than the oscillation frequency. For panel C, plots accounted for valid traces for all values of injected current at each noise level. These analyses were performed for valid traces from the 155 θ+ model neurons shown in Fig. 5*A*–*C*. All analyses were performed on the entire 5 s duration of the recorded traces.

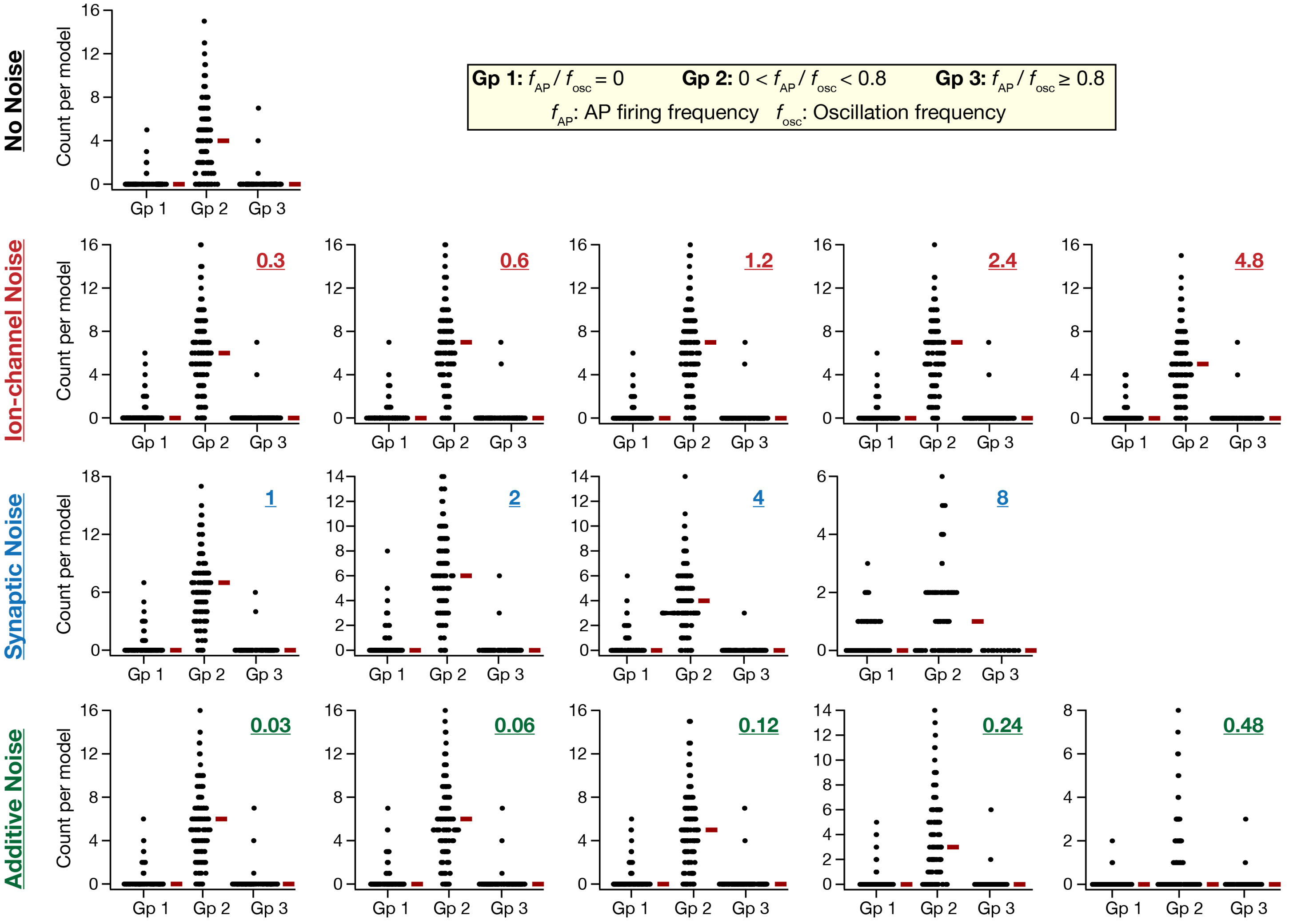

Supplementary Figure S11: Number of valid traces exhibiting distinct types of peri-threshold oscillatory activity for each θ+ model across different levels and types of noise. Valid oscillatory traces from any given model were divided into three groups based on the relationship between action potential firing rate f_{AP} and oscillation frequency f_{osc}. The maximum number of valid oscillatory traces could be 21, the number of current injection values used for each model. Gp 1: No spikes, only subthreshold oscillations ($f_{\mathrm{AP}}/f_{\mathrm{osc}} = 0$). Gp 2: Rate of spikes less than oscillation frequency ($0 < f_{\mathrm{AP}}/f_{\mathrm{osc}} < 0.8$). Gp 3: Rate of spikes comparable to oscillation frequency ($f_{\mathrm{AP}}/f_{\mathrm{osc}} \geq 0.8$). Bee-swarm plots represent the number of valid oscillatory traces in each model belonging to these groups. The thick red line by the side of the bee-swarm plot represents the respective median value of the represented distribution. The numbers provided on the top right of each plot indicate the noise level. The no-noise scenario is plotted on *Row 1*. These analyses were performed for valid traces from the 155 θ+ model neurons shown in Fig. 5*A–C*. All analyses were performed on the entire 5 s duration of the recorded traces.

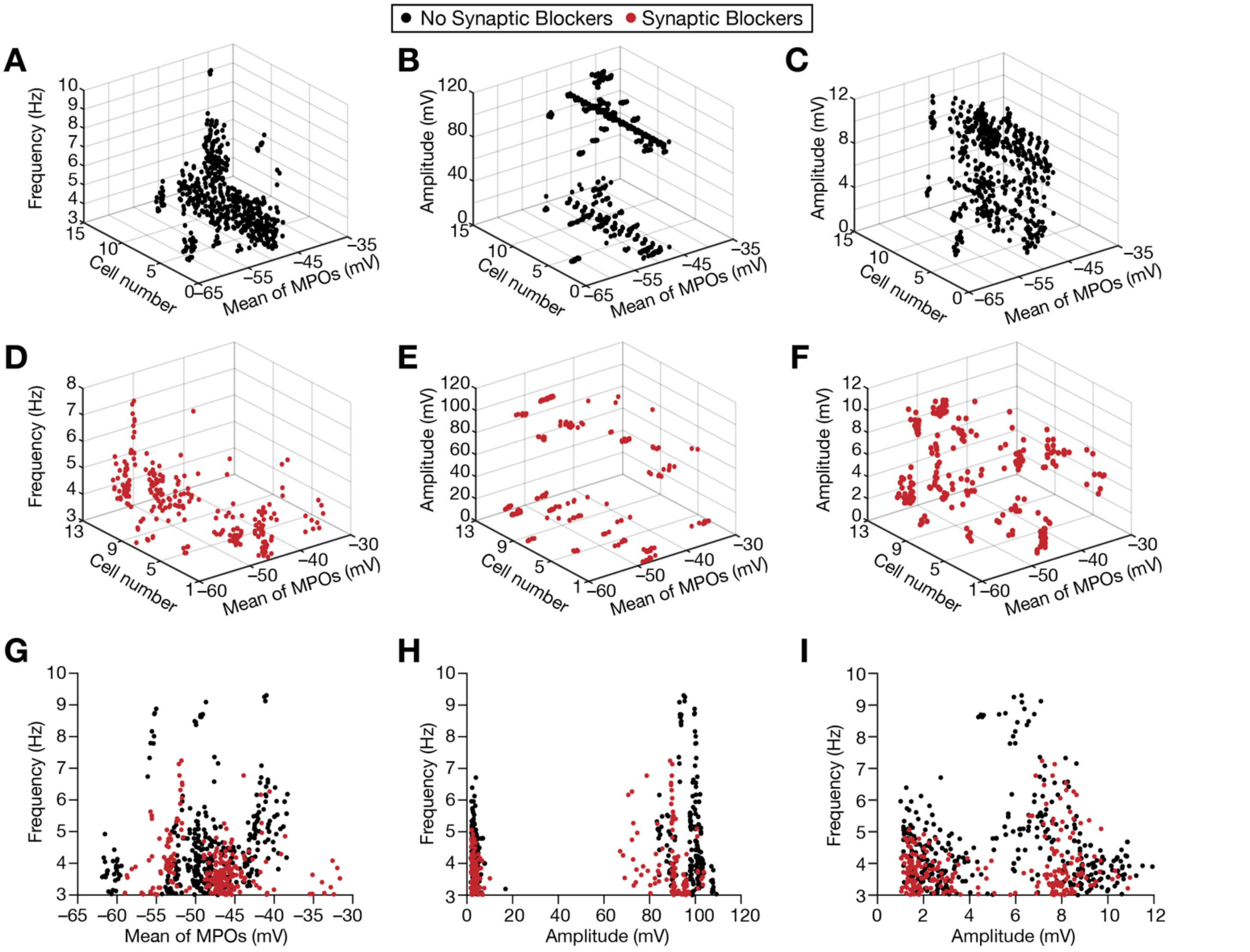

Supplementary Figure S12: Heterogeneities in peri-threshold intrinsic oscillations observed in electrophysiological recordings from rat LII MEC stellate cells. (A–C) 3D-plots depicting conjunctive heterogeneities in frequency (A) or amplitude (B–C) and mean membrane potential where oscillatory traces were detected, across different cells. Amplitude plots are shown for unfiltered (B) traces to emphasize the manifestation of mixed-mode oscillations, or for median-filtered (C) traces to elucidate the amplitude and mean voltages of these oscillations when action potentials were removed. These traces were obtained in the absence of synaptic receptor blockers. (D–F) Same as panels (A–C), but for recordings obtained in the presence of synaptic receptor blockers. (G–I) Data from (A–F) are depicted in 2D plots. Black and red dots represent measurements obtained without or with synaptic receptor blockers.

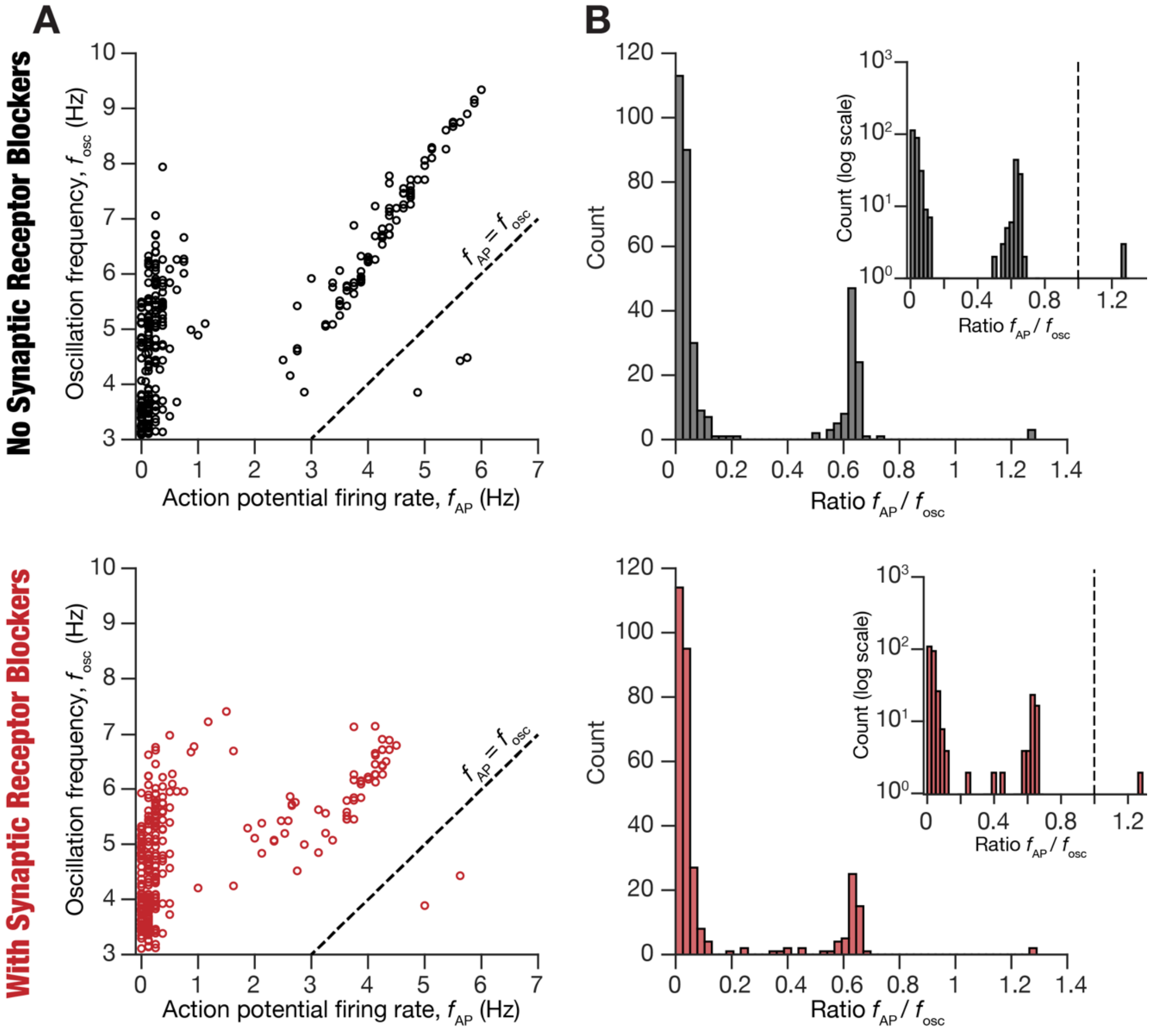

Supplementary Figure S13: The relationship between action potential firing rate and oscillation frequency distinguished different peri-threshold activity patterns in electrophysiological recordings. The top and the bottom panels represents outcomes of analyses of electrophysiological experiments performed without and with synaptic blockers, respectively. (A) Oscillation frequency plotted against action potential firing rate for each valid voltage trace. The dashed line represents the $f_{\mathrm{AP}}/f_{\mathrm{osc}} = 1$ line. f_{AP}: action potential firing rate. f_{osc}: oscillation frequency of the valid oscillatory trace. (B) Histogram of ratio between action potential firing rate f_{AP} and oscillation frequency f_{osc} across all valid voltage traces. Inset: histogram of $f_{\mathrm{AP}}/f_{\mathrm{osc}}$ in log scale. The dashed line represents the $f_{\mathrm{AP}}/f_{\mathrm{osc}} = 1$ line. The trimodal nature of the histogram might be noted. A large proportion of traces showed no spikes or very few spikes (proportion of traces with no spikes ($f_{\mathrm{AP}}/f_{\mathrm{osc}} = 0$): no synaptic blockers: 17.68%, with synaptic blockers: 17.43%; proportion of traces with $0 < f_{\mathrm{AP}}/f_{\mathrm{osc}} < 0.1$ where there are very few spikes ($f_{\mathrm{AP}} \leq 1$ Hz): no synaptic blockers: 51.3%, with synaptic blockers: 55.9%). The second mode contained traces where $f_{\mathrm{AP}}/f_{\mathrm{osc}}$ centered around 0.6 (proportion of traces with $0.5 \leq f_{\mathrm{AP}}/f_{\mathrm{osc}} \leq 0.7$: no synaptic blockers: 22.9%, with synaptic blockers: 17.11%), where the firing frequency was still less than the oscillation frequency. A very small proportion of traces had $f_{\mathrm{AP}}/f_{\mathrm{osc}} \geq 1$ (proportion of traces with $f_{\mathrm{AP}}/f_{\mathrm{osc}} \geq 1$: no synaptic blockers: 0.87%, with synaptic blockers: 0.66%). These analyses showed that spikes were sparse in most valid oscillatory traces showing mixed mode oscillations.

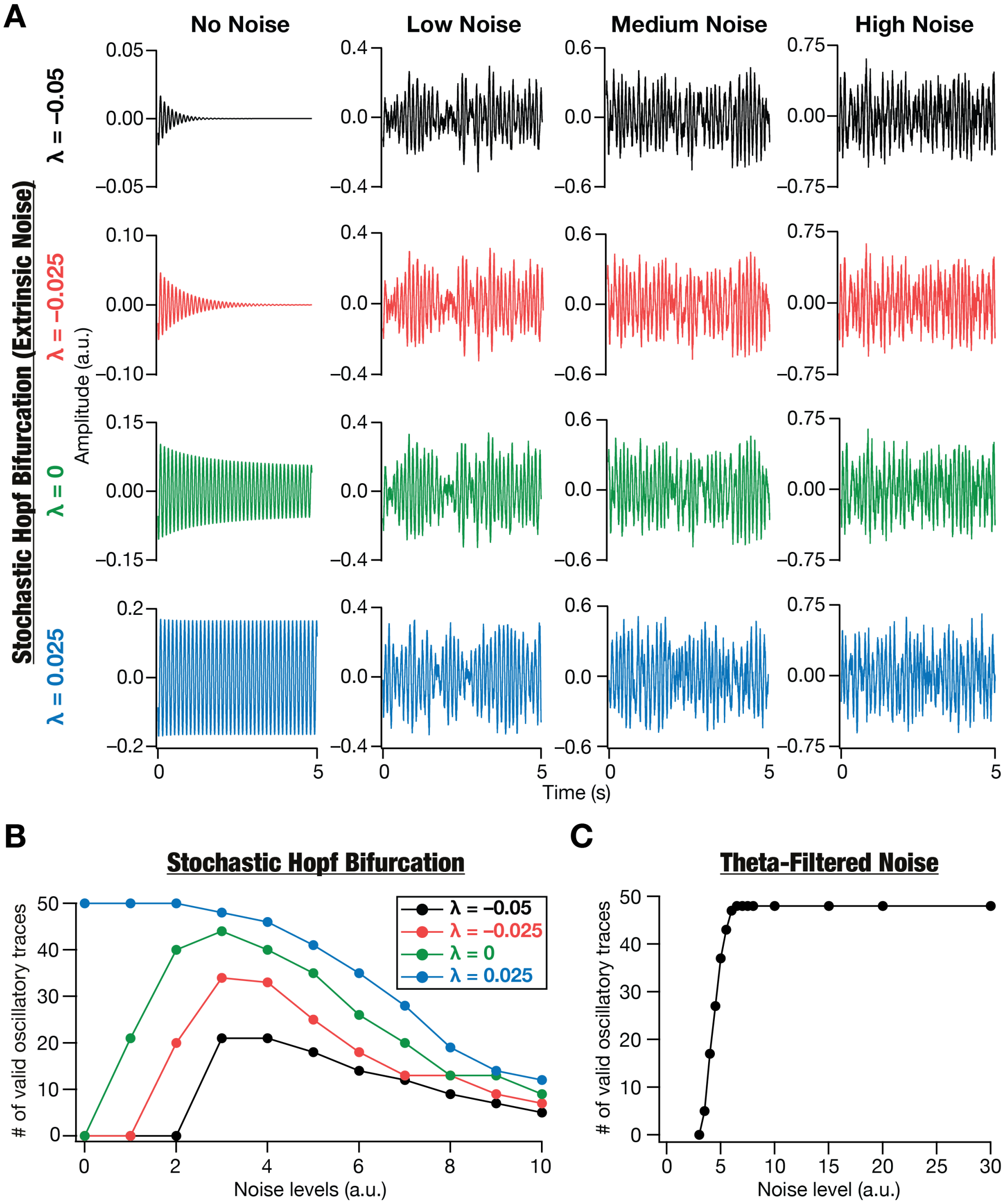

Supplementary Figure S14: Stochastic resonance distinguishes filtered noise traces from noise-induced dynamics of a deterministic system manifesting a bifurcation yielding stable limit cycles. (A) Impact of different levels of Gaussian white noise (Low, Medium, High) on the dynamics of a deterministic Hopf bifurcation system, shown for different values of the bifurcation parameter λ. Note the emergence of stable oscillations in the deterministic system ("No Noise") with $\lambda > 0$, and inward spirals with $\lambda \leq 0$. (B) The number of valid oscillatory traces at different levels of noise for various values of the bifurcation parameter λ. Validation was performed on the outcomes of 50 trials for each value of λ at level of noise. Note the manifestation of stochastic resonance when $\lambda \leq 0$: there is an optimal level of noise where the number of valid oscillatory traces is maximal, with the number falling on either side of this optimal level of noise. (C) The number of valid oscillatory traces at different levels of noise for various theta-filtered Gaussian white noise traces. Validation was performed on the outcomes of 50 trials for each level of noise. Notice a saturating monotonic increase in the number of valid traces with increase in noise level, pointing to the absence of stochastic resonance with theta-filtered oscillatory traces.

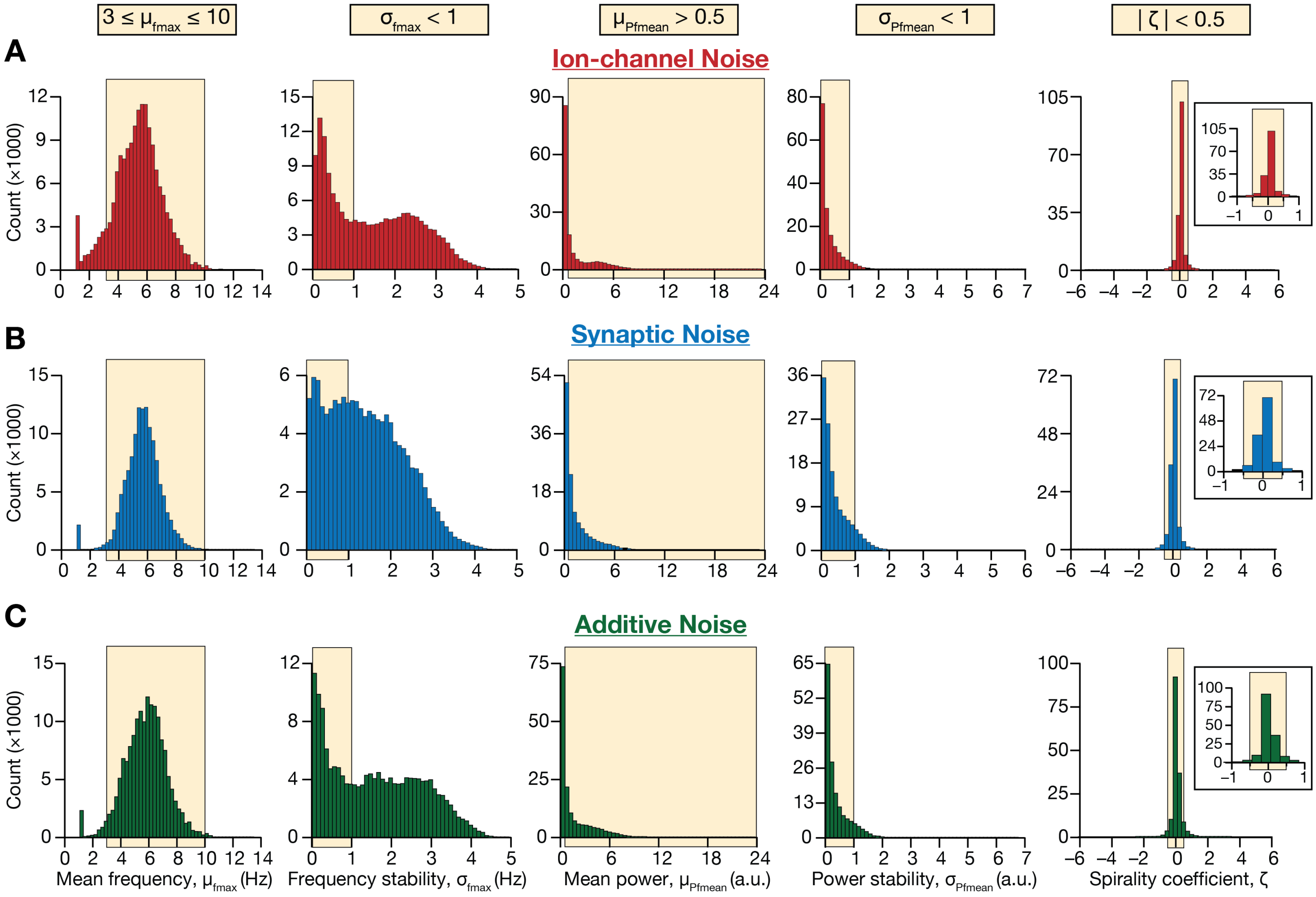

Supplementary Figure S15: Distributions of spectrogram-based quantitative metrics used for assessing robustness in theta-frequency oscillatory activity in a stochastic, heterogeneous population of entorhinal stellate cells. Distribution of the five spectrogram-based quantitative metrics for assessing robustness in theta-frequency oscillatory activity. *Column 1*, Mean frequency at maximal power, μ_{fmax}; *Column 2*, Standard deviation of frequency at maximal power, σ_{fmax}; *Column 3*, Mean power at mean frequency, μ_{Pfmean}; *Column 4*, Standard deviation of power at mean frequency, σ_{Pfmean}; *Column 5*, Spirality coefficient, ζ. This population of measurements were derived from all model neurons (n = 155), spanning all 21 current injection (I_{inj}) values, 10 independent trials for each level of the three forms of noise: (A) ion-channel noise, (B) synaptic noise, or (C) additive noise. The beige rectangle in each graph represents the bounds on the respective measurements for detection of a valid oscillatory trace.

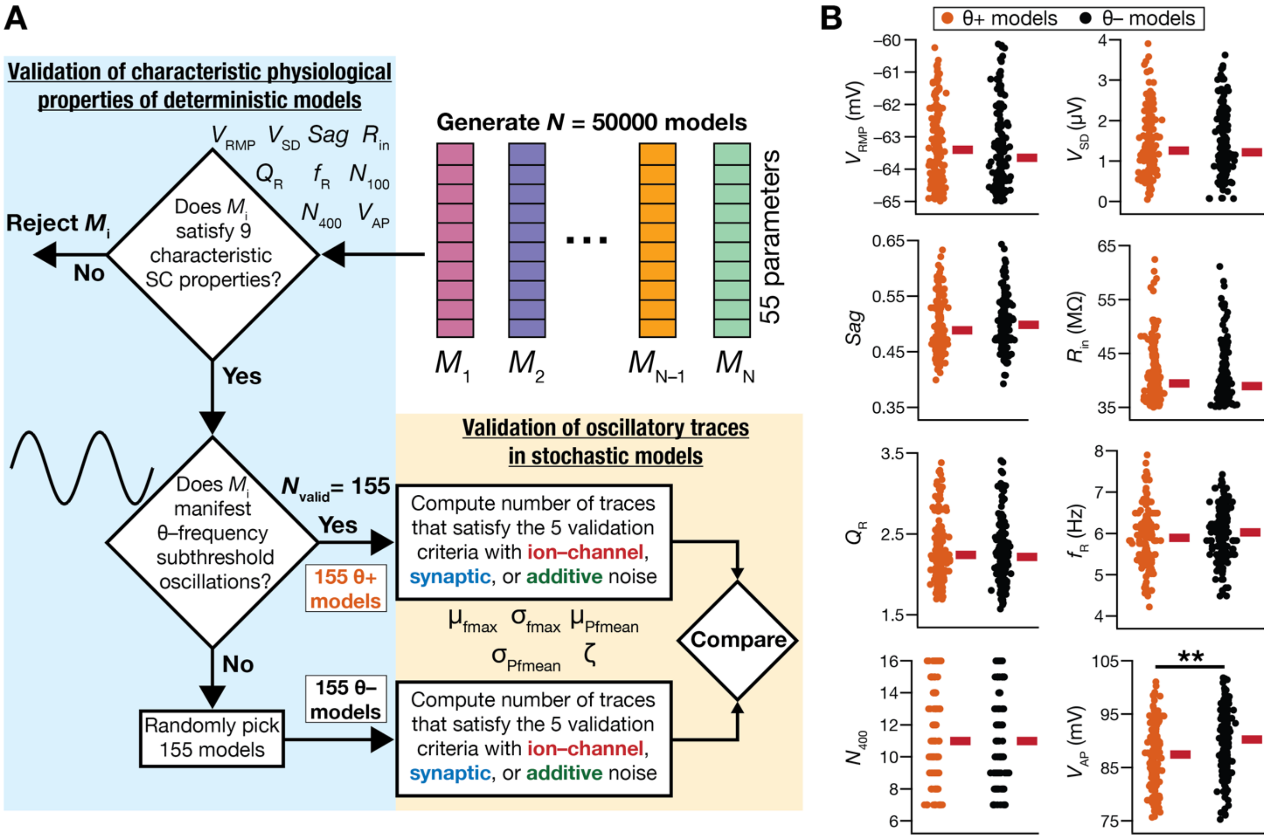

Supplementary Figure S16: Definitions and measurement distributions of θ+ and θ– stellate cell models. (A) Flowchart representing the two-stage validation process, the first involving characteristic physiological properties of deterministic models (blue rectangle) and the second concerned with oscillatory traces in stochastic models (beige rectangle). Deterministic models that satisfied all ten characteristic physiological properties of entorhinal cortical stellate cells (14) were defined as θ+ models. Models that satisfied nine of the ten characteristic physiological properties, but did not manifest deterministic, peri-threshold, theta-frequency oscillations were referred to as θ– models. (B) Distributions of the characteristic physiological properties of θ+ and θ– models. Shown are eight of the ten characteristic properties. As per the associated validation criterion, N_{100}, the number of action potentials fired for a 100 pA current injection, was zero for all θ+ and θ– models (14). The thick red line besides each measurement population represents the median for that population. *n* = 155 for both θ+ and θ– models. ***p*<0.005, Wilcoxon rank sum test. *p* value for V_{AP}= 2.24×10^{-4}. The *p* values for comparisons of all other measurements between θ+ and θ– models were >0.05.

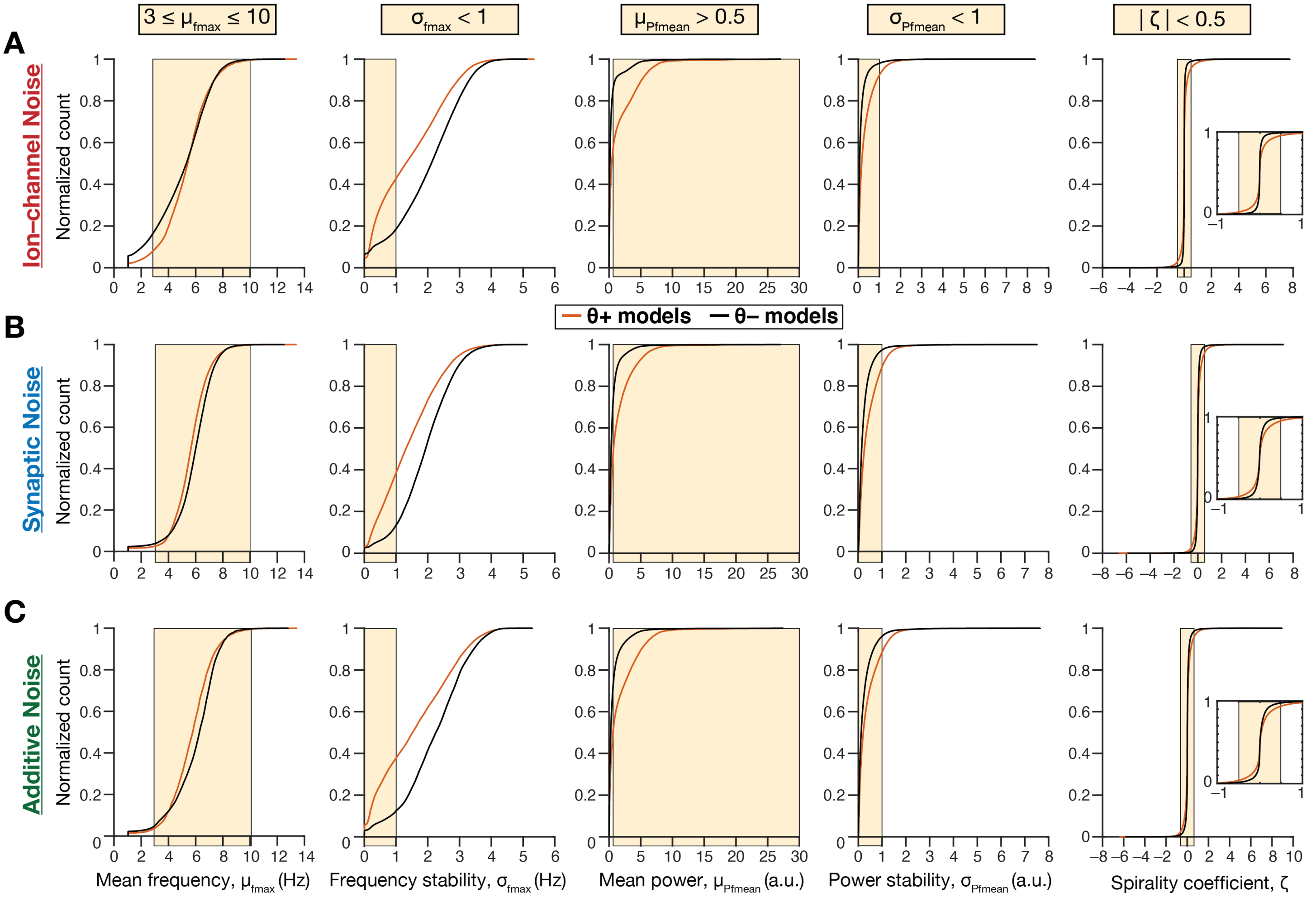

Supplementary Figure S17: Cumulative distributions of spectrogram-based quantitative metrics used for assessing robustness in theta-frequency oscillatory activity in θ+ and θ– model cell populations. Cumulative distributions of the five spectrogram-based quantitative metrics for assessing robustness in theta-frequency oscillatory activity in θ+ and θ– model cell populations. *Column 1*, Mean frequency at maximal power, μ_{fmax}; *Column 2*, Standard deviation of frequency at maximal power, σ_{fmax}; *Column 3*, Mean power at mean frequency, μ_{Pfmean}; *Column 4*, Standard deviation of power at mean frequency, σ_{Pfmean}; *Column 5*, Spirality coefficient, ζ. These populations of measurements were derived from all all θ+ ($n_{\theta+}$ = 155) and θ– ($n_{\theta-}$ = 155), spanning all 21 current injection (I_{inj}) values, 10 independent trials for each level of the three forms of noise: (A) ion-channel noise, (B) synaptic noise, or (C) additive noise. The beige rectangle in each graph represents the bounds on the respective measurements for detection of a valid oscillatory trace. Overall, the θ+ and θ– distributions for μ_{fmax} and ζ were comparable within their respective validation bounds. There was a larger fraction of θ– model traces (compared to θ+ model traces) that failed the frequency stability criterion (*i.e.*, σ_{fmax} ≤ 1 Hz). Although the power stability criterion on σ_{Pfmean} was satisfied by a larger proportion of θ– model traces (compared to θ+ model traces), this was simply because of the overall low power of oscillations in traces from θ– models. There was a higher proportion of θ– model traces that failed the minimum power requirement ($\mu_{\text{Pfmean}} > 0.5$) compared to their θ+ counterparts.

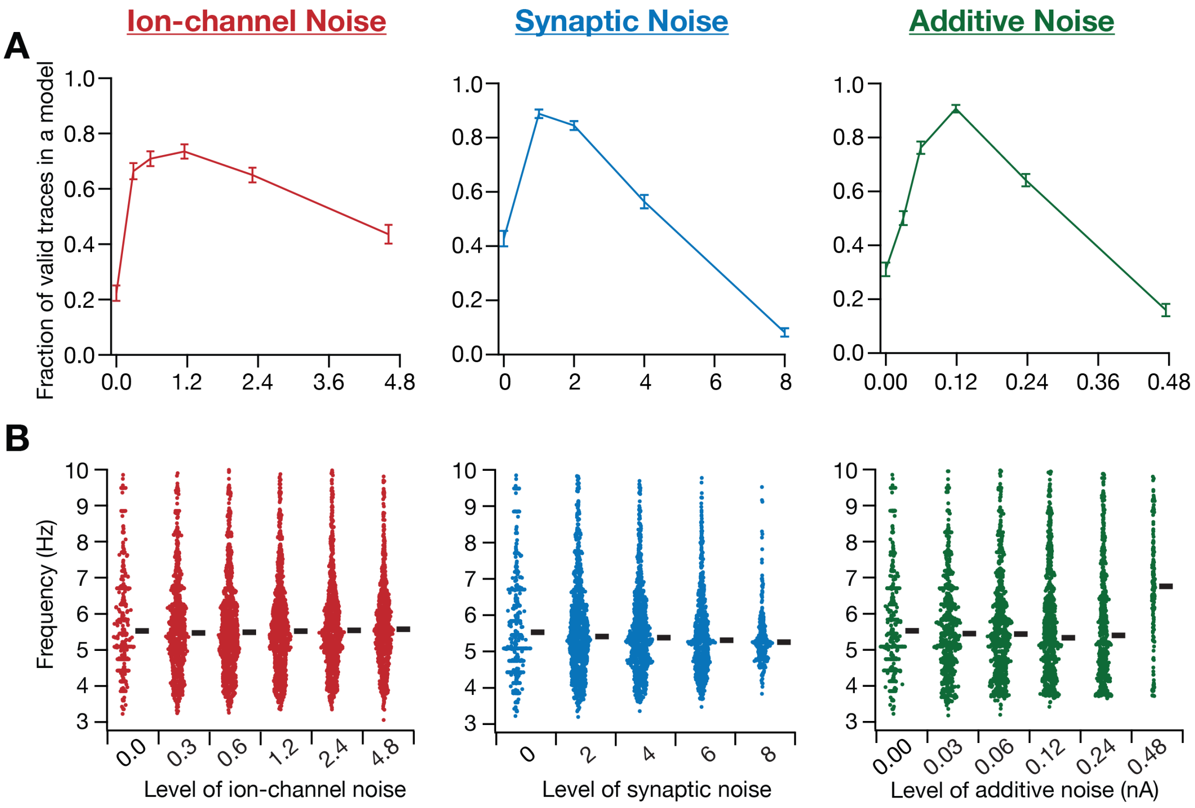

Supplementary Figure S18: Stochastic resonance in the emergence of peri-threshold theta-frequency oscillations at the single-neuron level. (A) The fraction of valid oscillatory traces, normalized for each neuron across noise levels, plotted (mean ± SEM) as a function of level of each form of noise. (B) Oscillatory frequency of all valid oscillatory traces spanning different levels of each form of noise. The thick black line beside each plot represents the median for those set of frequencies.

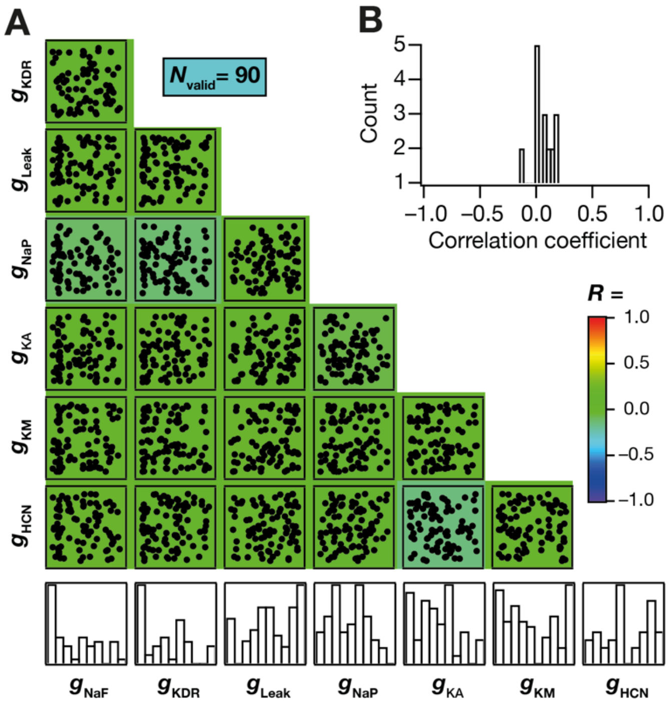

Supplementary Figure S19: Expression of cellular scale ion-channel degeneracy in a heterogeneous population of stochastic stellate cell models. (A) Pair-wise scatter plots between the 7 parameters for all the valid LII MEC stellate cell models (N_{valid} = 90) with stochastically gated ion channels. Individual scatter plots are overlaid on a heat map that depicts the pair-wise correlation coefficient computed for that scatter plot. The bottommost row depicts the histograms for the corresponding parameters in the valid heterogeneous population, showing a widespread distribution of the ion-channel conductances that yielded models with characteristic signatures of MEC stellate cells. (B) Distribution of correlation coefficient values of 21 unique pairs between 7 parameters from scatter plots in (A). All pairwise correlations are weak, suggesting the absence of pairwise compensations in these parameters towards achieving characteristic physiological signatures of MEC stellate cells.

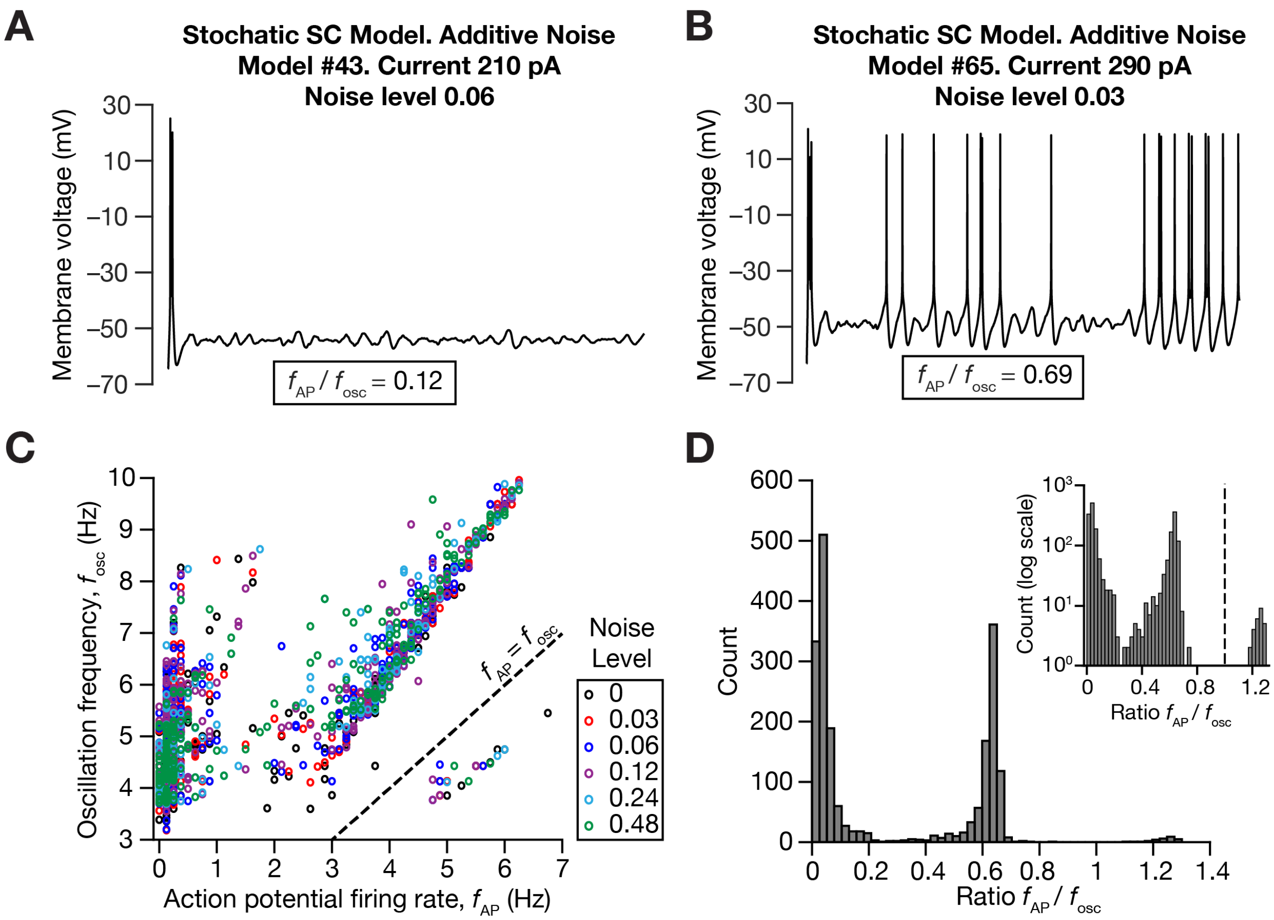

Supplementary Figure S20: The relationship between action potential firing rate and oscillation frequency distinguished different peri-threshold activity patterns in SC models with stochastically gated ion channels. (A–B) Example traces of valid oscillatory patterns with two different values of $f_{\mathrm{AP}}/f_{\mathrm{osc}}$. f_{AP}: action potential firing rate and f_{osc}: oscillation frequency of the valid oscillatory trace. Both traces span a 5-s period. (C) Oscillation frequency plotted against action potential firing rate for each valid voltage trace. The dashed line represents the $f_{\mathrm{AP}}/f_{\mathrm{osc}} = 1$ line. Different colors represent different noise levels. (D) Histogram of $f_{\mathrm{AP}}/f_{\mathrm{osc}}$ across all valid voltage traces. Inset: histogram of $f_{\mathrm{AP}}/f_{\mathrm{osc}}$ in log scale. The dashed line represents the $f_{\mathrm{AP}}/f_{\mathrm{osc}} = 1$ line. The trimodal nature of the histogram may be noted. A large proportion of traces showed no spikes or very few spikes (proportion of traces with no spikes ($f_{\mathrm{AP}}/f_{\mathrm{osc}} = 0$): 6.55%; proportion of traces with $0 < f_{\mathrm{AP}}/f_{\mathrm{osc}} < 0.1$ where there are very few spikes ($f_{\mathrm{AP}} \leq 1$ Hz): 46.8%). The second mode contained traces where $f_{\mathrm{AP}}/f_{\mathrm{osc}}$ centered around 0.6 (proportion of traces with $0.5 \leq f_{\mathrm{AP}}/f_{\mathrm{osc}} \leq 0.7$: 37.7%), where the f_{AP} was still less than f_{osc}. A very small proportion of traces had $f_{\mathrm{AP}}/f_{\mathrm{osc}} \geq 1$ owing to bursting behavior in some cycles (proportion of traces with $f_{\mathrm{AP}}/f_{\mathrm{osc}} \geq 1$: 1.18%). Together valid oscillatory traces showed pure subthreshold oscillations or mixed-mode oscillatory patterns where sub-threshold oscillations and spikes coexisted, with spikes skipping oscillatory cycles in these traces even in scenarios where $f_{\mathrm{AP}}/f_{\mathrm{osc}} > 1$ (examples with different $f_{\mathrm{AP}}/f_{\mathrm{osc}}$ are in Fig. S8 and panels *A–B*). These analyses showed that spikes were sparse in most valid oscillatory traces showing mixed mode oscillations.

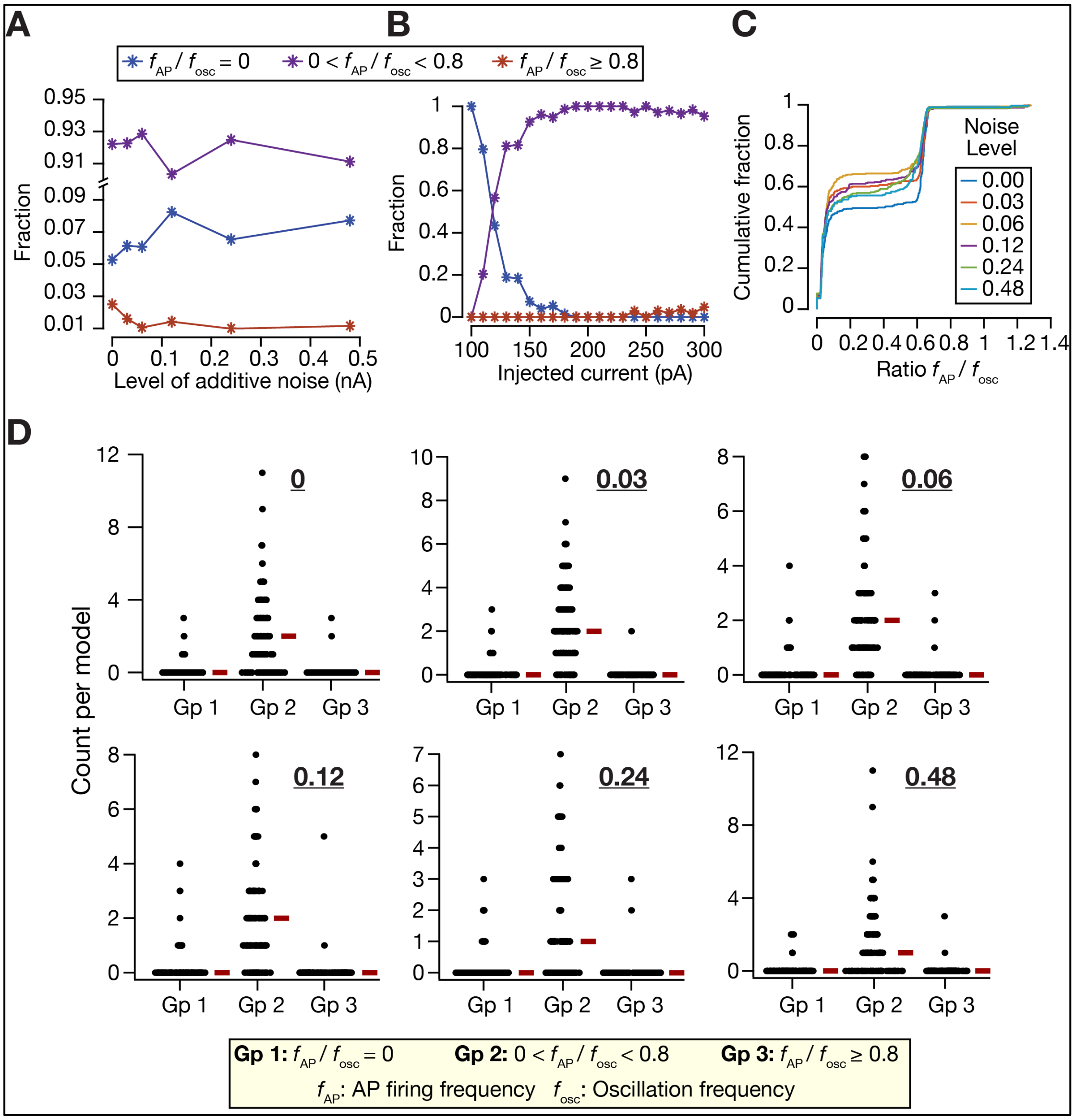

Supplementary Figure S21: Proportions of valid traces exhibiting distinct types of peri-threshold oscillatory activity for each model across different levels of noise in stochastic SC models. (A–B) Valid oscillatory traces were divided into three groups based on the relationship between action potential firing rate f_{AP} and oscillation frequency f_{osc}. Group 1 (blue): No spikes, only subthreshold oscillations ($f_{\mathrm{AP}}/f_{\mathrm{osc}} = 0$). Group 2 (purple): Rate of spikes less than oscillation frequency ($0 < f_{\mathrm{AP}}/f_{\mathrm{osc}} < 0.8$). Group 3 (red): Rate of spikes comparable to oscillation frequency ($f_{\mathrm{AP}}/f_{\mathrm{osc}} \geq 0.8$). The fraction of traces that belonged to each of these three groups were plotted for different types of noise as functions of level of noise (A) or the injected current, the bifurcation parameter (B). Across different types of noise and models, most traces belonged to the second group where the rate of spikes was less than the oscillation frequency ($0 < f_{\mathrm{AP}}/f_{\mathrm{osc}} < 0.8$), with a small fraction of traces falling into the first or the third groups (*A*). As a function of injected current, expectedly more valid Group 1 (sub-threshold oscillations) traces were present for lower current injections, switching to Group 2 with increasing values of current injections (*B*). Plots in panels A and B contain pooled valid traces for all current injection values and all noise levels, respectively. (C) Cumulative distribution of $f_{\mathrm{AP}}/f_{\mathrm{osc}}$ plotted for different levels of different forms of noise. Across different models and forms of noise, it may be noted that a vast majority of traces fall into either Group 1 or Group 2, indicating that the AP firing rate in these valid oscillatory traces was less than the oscillation frequency. For panel C, plots contain valid traces for all values of injected current at each noise level. (D) Bee-swarm plots represent the number of valid oscillatory traces in each model belonging to the three groups. The thick red line by the side of the bee-swarm plot showing the distributions represents the respective median value. The numbers provided on the top right of each plot indicate the noise level. All analyses were performed on the entire 5 s duration of the recorded traces. All analyses were performed with valid SC models with stochastically gated ion channels (n = 90) and on the entire 5 s duration of the recorded traces.

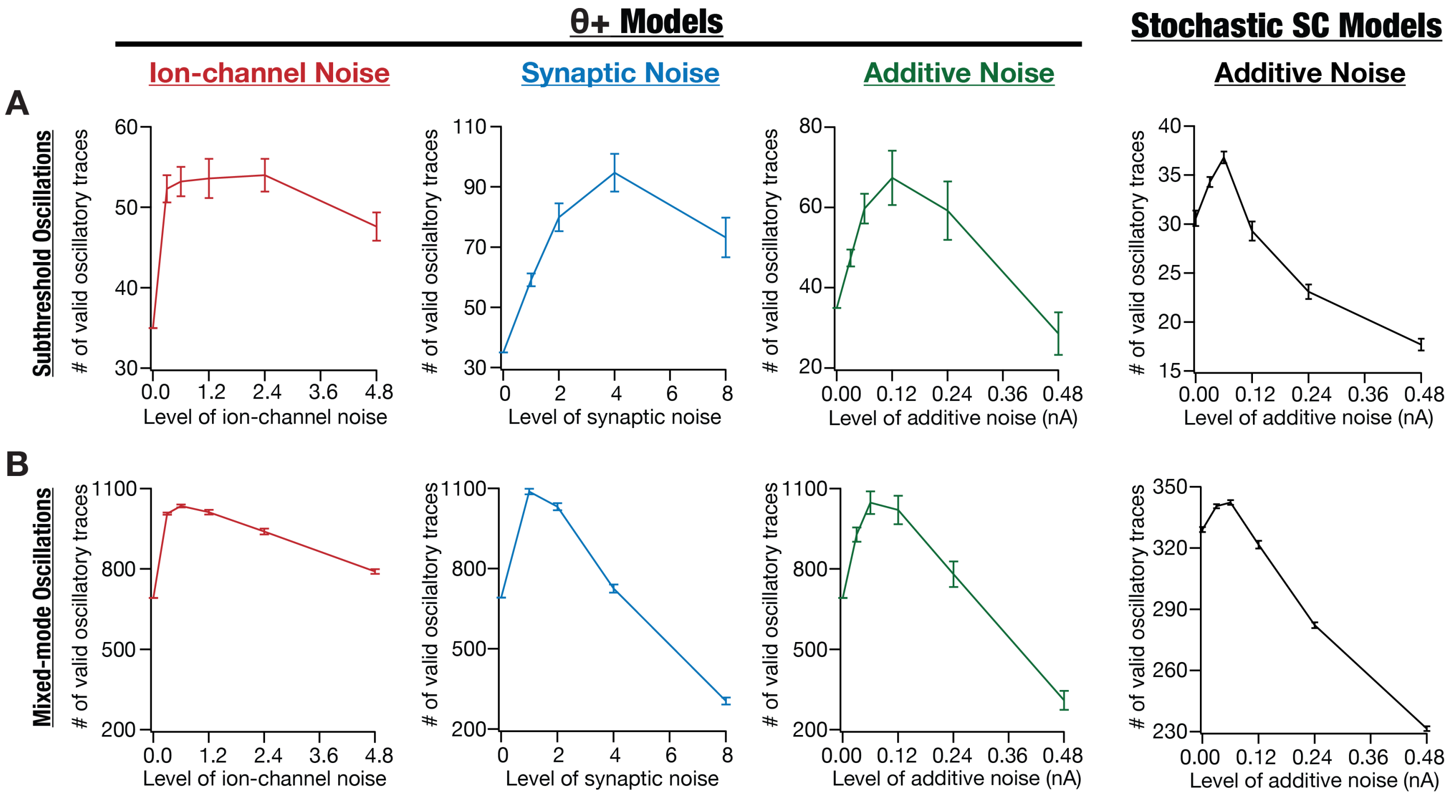

Supplementary Figure S22: Manifestation of stochastic resonance in subthreshold and mixed-mode oscillatory traces. Mean and SEM of number of valid subthreshold oscillatory traces (A) and valid mixed-mode oscillatory traces (B) across different noise levels and three distinct kinds of noise from θ+ model neurons with deterministically gated ion channels (*Columns 1–3*; corresponds to θ+ model neurons shown in Fig. 5*A–C*) and stochastically gated ion channels (*Column 4*; corresponds to Fig. 7*F*). The number of valid oscillatory traces is plotted as mean and SEM spanning 10 independent trials for each level of the three forms of noise, for all θ+ ($n_{\theta+}$ = 155) models (*Columns 1–3*). Mean and SEM of number of valid oscillatory traces from all SC models (*N* = 90) with stochastic gating ion channels, computed at six different levels of additive noise for 10 independent trials (*Column 4*).

Supplementary Table S1: Measurement statistics from stellate cells in the absence of synaptic blockers.

No.	Measurement	Mean	SEM	SD	CV	Median	IQR
1	V_{RMP} (mV)	–58.79	0.76	2.93	–0.05	–58.79	2.89
2	S_{α}	0.79	0.04	0.14	0.18	0.74	0.23
3	R_{in} (MΩ)	42.02	4.68	18.11	0.43	41.44	32.86
4	*Sag*	0.62	0.02	0.09	0.15	0.60	0.11
5	f_{R} (Hz)	6.10	0.42	1.64	0.27	5.91	2.26
6	$\lvert Z\rvert_{\mathrm{max}}$ (MΩ)	74.54	9.74	37.73	0.51	67.32	38.33
7	Q_{R}	1.79	0.11	0.43	0.24	1.79	0.66
8	Φ_{L} (rad.Hz)	0.58	0.12	0.45	0.77	0.41	0.60
9	V_{th} (mV)	–43.84	1.75	5.26	–0.12	–42.43	4.67
10	V_{AP} (mV)	103.61	1.56	4.69	0.05	105.07	5.34
11	*SFA*	0.39	0.07	0.19	0.49	0.39	0.28
12	T_{1AP} (ms)	28.84	8.83	26.49	0.92	27.20	17.35
13	T_{APHW} (ms)	0.80	0.07	0.20	0.24	0.72	0.08
14	$\left.\frac{dV}{dt}\right\rvert_{\mathrm{AP}}^{\mathrm{max}}$ (V/s)	441.82	24.13	72.39	0.16	419.91	119.63

Supplementary Table S2: Measurement statistics from stellate cells in the presence of synaptic blockers.

No.	Measurement	Mean	SEM	SD	CV	Median	IQR
1	V_{RMP} (mV)	–58.87	0.94	3.38	–0.06	–58.21	3.33
2	S_{α}	0.82	0.02	0.06	0.07	0.81	0.07
3	R_{in} (MΩ)	38.68	3.63	13.09	0.34	37.18	14.38
4	*Sag*	0.69	0.02	0.08	0.11	0.71	0.09
5	f_{R} (Hz)	5.32	0.34	1.22	0.23	5.25	1.55
6	$\lvert Z\rvert_{\mathrm{max}}$ (MΩ)	70.38	7.17	25.84	0.37	61.03	29.94
7	Q_{R}	1.53	0.07	0.26	0.17	1.51	0.32
8	Φ_{L} (rad.Hz)	0.40	0.06	0.20	0.51	0.40	0.31
9	V_{th} (mV)	–41.74	1.63	4.88	–0.12	–41.01	7.40
10	V_{AP} (mV)	100.30	1.85	5.56	0.06	100.62	4.20
11	*SFA*	0.36	0.10	0.21	0.60	0.23	0.24
12	T_{1AP} (ms)	49.63	26.19	78.57	1.58	22.42	19.34
13	T_{APHW} (ms)	0.71	0.02	0.06	0.08	0.70	0.10
14	$\left.\frac{dV}{dt}\right\rvert_{\mathrm{AP}}^{\mathrm{max}}$ (V/s)	394.21	16.69	50.06	0.13	421.13	92.16

SEM: Standard error of the mean; **SD**: Standard deviation; **CV**: Coefficient of variation; **IQR**: Interquartile range.

Supplementary Table S3: Range of parameters used in generating the heterogeneous model population of stellate cells with stochastic ion channels.

No	Parameter	Description	Min	Max
**1**	$\bar{g}_{\mathrm{Na}F}$ (mS/cm^2)	Maximal conductance of fast sodium (NaF) channel	1.61	6.52
**2**	$\bar{g}_{\mathrm{KDR}}$ (mS/cm^2)	Maximal conductance of delayed rectifier potassium (KDR) channel	0.86	3.45
**3**	$\bar{g}_{\mathrm{HCN}}$ (mS/cm^2)	Maximal conductance of Hyperpolarization-activated cyclic-nucleotide-gated (HCN) channel	0.65	2.6
**4**	$\bar{g}_{\mathrm{Na}P}$ (mS/cm^2)	Maximal conductance of Persistent sodium (NaP) channel	0.01	0.04
**5**	$\bar{g}_{KA}$ (mS/cm^2)	Maximal conductance of *A*-type potassium (KA) channel	0.0525	0.21
**6**	$\bar{g}_{\mathrm{KM}}$ (mS/cm^2)	Maximal conductance of *M*-type potassium (KM) channel	0.0525	0.21
**7**	g_{Leak} (mS/cm^2)	Maximal conductance of Leak channels	0.0125	0.05

Supplementary Table S4: Physiologically relevant range of LII stellate cell measurements. Experimental bounds on each intrinsic measurement involved in the validation process of stochastically generated models. The bounds were derived from previous electrophysiological measurements (4, 26), and are the same as (14).

No	Intrinsic electrophysiological measurement	Valid range
**1**	Resting membrane potential, V_{RMP} (mV)	–65 to –60
**2**	Sag ratio	0.35 to 0.65
**3**	Input resistance, R_{in} (MΩ)	35 to 65
**4**	Number of APs for a 100 pA step current for 500 ms, N_{100}	0
**5**	Number of APs for a 400 pA step current for 500 ms, N_{400}	7 to 16
**6**	AP amplitude, V_{AP} (mV)	>75

Supplementary Table S5: Roles of different ion-channel conductances in mediating different aspects of peri-threshold activity, assessed from this study and evidence from other studies. The role of individual ion-channel conductances were analyzed using virtual knockout models (Fig. 7*A* and (27)). Ion-channel degeneracy manifests as a result of the overlapping roles of different ion channels, the involvement of different mediating and modulating mechanisms for each physiological outcome, and the ability of several possible combinations to execute an emergent function (27-29). Heterogeneities in the expression profiles of these channels across different SC neurons introduce heterogeneities in the neuronal properties, including in peri-threshold oscillations. Despite heterogeneities in ion-channel expression, ion-channel degeneracy provides a substrate for robustness in physiology, by allowing for disparate structural components to elicit similar characteristic physiology. Finally, stochasticity in channel gating and synaptic properties introduces stochasticity in the bifurcations associated with the physiological properties and introducing stochastic resonance as an additional robustness mechanism. The references listed against each ion-channel subtype provide computational and electrophysiological/pharmacological lines of evidence related to that ion-channel subtype.

No.	Channel Subtype	Characteristic SC Peri-threshold Oscillatory Properties Associated with Channel
**1**	Fast sodium (NaF) channel	• Modulatory role in peri-threshold oscillations. Results in Fig. 7*A* show peri-threshold oscillations even in the absence of fast sodium channels<br>• Potential role in mediating the fast positive feedback loop abstracted in Fig. 8<br>• Mediating role in spike generation<br>• Role in spike clustering during mixed-mode oscillations through fast recovery from inactivation during strong afterhyperpolarization (AHP) following the previous spike<br>• **References**: (27, 30)
**2**	Delayed rectifier potassium (KDR) channel	• Modulatory role in peri-threshold oscillations. Results in Fig. 7*A* are consequent to depolarization-induced block upon deletion of KDR channels<br>• Mediating role in spike repolarization and AHP kinetics.<br>• Role in spike clustering during mixed-mode oscillations through strong AHP that is essential for post-inhibitory rebound and sodium-channel recovery from inactivation<br>• **References**: (27, 30)
**3**	Persistent sodium (NaP) channel	• Mediating role in peri-threshold theta-frequency oscillations. Results in Fig. 7*A* show complete abolition of peri-threshold oscillations in the absence of persistent sodium channels<br>• The principal mediating mechanism for the fast positive feedback loop abstracted in Fig. 8. Acts as an amplifying conductance<br>• Mediating role in rising phase of spikes<br>• Role in spike clustering during mixed-mode oscillations through amplification of post-inhibitory rebound that follows strong AHP following the previous spike<br>• **References**: (27, 30-40)
**4**	HCN channel	• Mediating role in peri-threshold theta-frequency oscillations. Results in Fig. 7*A* show reduced number of valid peri-threshold oscillatory traces in the absence of HCN channels.<br>• A principal mediating mechanism for the slow negative feedback loop abstracted in Fig. 8. Acts as a resonating conductance<br>• Mediating role in medium AHP (mAHP) following strong spike-mediated deactivation<br>• Mediating role in spike clustering during mixed-mode oscillations through amplification of mAHP that is essential for post-inhibitory rebound and sodium-channel recovery from inactivation<br>• Mediating role in spike clustering during mixed-mode oscillations by mediating post-inhibitory rebound following slow and strong activation by the AHP from the previous spike<br>• **References**: (27, 30, 31, 33-35, 37, 39-48)

Supplementary Table S5 (Continued)

No.	Channel Subtype	Characteristic SC Peri-threshold Oscillatory Properties Associated with Channel
**5**	*M*-type potassium (KM) channel	• Mediating role in peri-threshold theta-frequency oscillations. Results in Fig. 7*A* show complete abolition of peri-threshold oscillations in the absence of KM channels<br>• A principal mediating mechanism for the slow negative feedback loop abstracted in Fig. 8. Acts as a resonating conductance<br>• Mediating role in mAHP following strong spike-mediated activation<br>• Mediating role in spike clustering during mixed-mode oscillations through amplification of mAHP that is essential for post-inhibitory rebound and sodium-channel recovery from inactivation<br>• Mediating role in spike clustering during mixed-mode oscillations by mediating post-inhibitory rebound following slow and strong deactivation by the AHP from the previous spike<br>• **References**: (27, 30, 31, 40, 49)
**6**	*A*-type potassium (KA) channel	• Modulatory role in peri-threshold oscillations (Fig. 7*A*)<br>• Mediating role in spike repolarization and AHP amplitude and kinetics<br>• Role in spike clustering during mixed-mode oscillations through strong AHP that is essential for post-inhibitory rebound and sodium-channel recovery from inactivation<br>• **References**: (27, 30)
**7**	Calcium channels	• Modulatory role in peri-threshold oscillations (Fig. 7*A*)<br>• Mediating role in spike bursts and associated depolarization<br>• Role in spike clustering through activation of calcium-activated potassium channels, which lead to strong AHP that is essential for post-inhibitory rebound and sodium-channel recovery from inactivation<br>• **References**: (27, 30)
**8**	Calcium-activated potassium channels	• Modulatory role in peri-threshold oscillations (Fig. 7*A*)<br>• Mediating role in spike repolarization and AHP amplitude and kinetics<br>• Mediating role in spike bursts and associated AHP<br>• Role in spike clustering during mixed-mode oscillations through strong AHP that is essential for post-inhibitory rebound and sodium-channel recovery from inactivation<br>• **References**: (27, 30, 50, 51)

Supplementary Table S6: Characteristic properties of MEC stellate cells captured by the different abstractions and models assessed in this study. Electrophysiological recordings and associated analyses are presented in Figs. 1, 4*F–H*, S1–S4, S12–S13.

No.	Abstraction/Model	Characteristic SC Intrinsic Properties Captured by the Abstraction/Model
**1**	Theta-filtered noise **(Figs. 2*B*, S9*C*)**	• Arhythmic, variable-amplitude, variable-frequency activity with spectral signatures comparable to SC peri-threshold oscillations
**2**	Stochastic Hopf bifurcation<br>**(Figs. 2*C*, S5, S13*A–B*)**	• Arhythmic, variable-amplitude, variable-frequency stable oscillations with noise, with spectral signatures comparable to SC peri-threshold oscillations<br>• Input- or state-dependent emergence of oscillations (λ: bifurcation parameter)<br>• Stochastic bifurcations (with noise) leading to input- and state-dependent emergence of oscillations<br>• Stochastic resonance in the emergence of oscillations
**3**	Heterogeneous population of MEC SC models with **deterministic** ion channel models showing theta-frequency oscillations (**θ+ models**) + 3 different forms of noise<br>**(Figs. 3, 4*A–E*, 5–6, 7*A*, S6–S11, S15–S18, S22)**	• Heterogeneous distribution of 10 different signature sub- and supra-threshold electrophysiological properties of MEC SCs (Fig. S16) (14)<br>• Heterogeneous deterministic (14) and stochastic (with noise) bifurcations (I_{inj}: bifurcation parameter) leading to input- and state-dependent emergence of oscillations<br>• Heterogeneous, arhythmic, variable-amplitude, variable-frequency stable oscillations with noise. with spectral signatures matching SC oscillations<br>• Heterogeneous deterministic and stochastic (with noise) mixed-mode oscillations<br>• Heterogeneous clustered spiking pattern and cycle-skipping in spike generation<br>• Heterogeneous stochastic resonance in the emergence of oscillations<br>• Critical requirement of specific ion channels (NaP, HCN & KM) in the emergence of peri-threshold oscillations in the absence (14) and presence of noise (Fig. 7)<br>• Ion-channel degeneracy in the emergence of characteristic physiology
**4**	Heterogeneous population of MEC SC models with **deterministic** ion channel models not showing theta-frequency oscillations (**θ– models**) + 3 different forms of noise<br>**(Figs. 5, S16–S17)**	• Heterogeneous distribution of 9 different signature sub- and supra-threshold electrophysiological properties of MEC SCs (Fig. S16) (14)<br>• Heterogenous stochastic (with noise) bifurcations (I_{inj}: bifurcation parameter) leading to input- and state-dependent emergence of oscillations<br>• Heterogeneous, arhythmic, variable-amplitude, variable-frequency stable oscillations with noise, with spectral signatures matching SC oscillations<br>• Heterogeneous deterministic and stochastic (with noise) mixed-mode oscillations<br>• Heterogeneous clustered spiking pattern and cycle-skipping in spike generation<br>• Heterogeneous stochastic resonance in the emergence of oscillations
**5**	Heterogeneous population of MEC SC models with **stochastic** ion channel models + Additive noise<br>**(Figs. 7*B–G*, S19–S22)**	• Heterogeneous distribution of 6 different signature sub- and supra-threshold electrophysiological properties of MEC SCs<br>• Heterogeneous stochastic bifurcations (I_{inj}: bifurcation parameter) leading to input- and state-dependent emergence of oscillations<br>• Heterogeneous, arhythmic, variable-amplitude, variable-frequency stable oscillations with spectral signatures matching SC oscillations<br>• Heterogeneous stochastic mixed-mode oscillations<br>• Heterogeneous clustered spiking pattern and cycle-skipping in spike generation<br>• Heterogeneous stochastic resonance in the emergence of oscillations<br>• Ion-channel degeneracy in the emergence of characteristic physiology<br>• Impact of stochasticity in ion channel gating properties on peri-threshold activity
**6**	Generalized network motif with feedback loops + Additive noise<br>**(Fig. 8)**	• Deterministic or stochastic (with noise) bifurcations (I: bifurcation parameter) leading to input-dependent emergence of oscillations<br>• Arhythmic, variable-amplitude, variable-frequency stable oscillations (with noise)<br>• Stochastic resonance in the emergence of oscillations

SUPPLEMENTARY REFERENCES

1. S. Ashhad, R. Narayanan, Active dendrites regulate the impact of gliotransmission on rat hippocampal pyramidal neurons. *Proc Natl Acad Sci U S A* **113**, E3280-3289 (2016).
2. P. Mishra, R. Narayanan, Heterogeneities in intrinsic excitability and frequency-dependent response properties of granule cells across the blades of the rat dentate gyrus. *Journal of neurophysiology* **123**, 755-772 (2020).
3. P. Mishra, R. Narayanan, Ion-channel degeneracy: Multiple ion channels heterogeneously regulate intrinsic physiology of rat hippocampal granule cells. *Physiol Rep* **9**, e14963 (2021).
4. A. Boehlen, C. Henneberger, U. Heinemann, I. Erchova, Contribution of near-threshold currents to intrinsic oscillatory activity in rat medial entorhinal cortex layer II stellate cells. *Journal of neurophysiology* **109**, 445-463 (2013).
5. P. D. Dodson, H. Pastoll, M. F. Nolan, Dorsal-ventral organization of theta-like activity intrinsic to entorhinal stellate neurons is mediated by differences in stochastic current fluctuations. *J Physiol* **589**, 2993-3008 (2011).
6. L. M. Giocomo, M. E. Hasselmo, Time constants of h current in layer ii stellate cells differ along the dorsal to ventral axis of medial entorhinal cortex. *J Neurosci* **28**, 9414-9425 (2008).
7. J. S. Haas, A. D. Dorval, 2nd, J. A. White, Contributions of Ih to feature selectivity in layer II stellate cells of the entorhinal cortex. *J Comput Neurosci* **22**, 161-171 (2007).
8. J. S. Haas, J. A. White, Frequency selectivity of layer II stellate cells in the medial entorhinal cortex. *Journal of neurophysiology* **88**, 2422-2429 (2002).
9. L. M. Giocomo, E. A. Zilli, E. Fransen, M. E. Hasselmo, Temporal frequency of subthreshold oscillations scales with entorhinal grid cell field spacing. *Science* **315**, 1719-1722 (2007).
10. R. Narayanan, D. Johnston, Long-term potentiation in rat hippocampal neurons is accompanied by spatially widespread changes in intrinsic oscillatory dynamics and excitability. *Neuron* **56**, 1061-1075 (2007).
11. R. Narayanan, D. Johnston, The h channel mediates location dependence and plasticity of intrinsic phase response in rat hippocampal neurons. *J Neurosci* **28**, 5846-5860 (2008).
12. E. M. Izhikevich, *Dynamical Systems in Neuroscience: The Geometry of Excitability and Bursting* (MIT Press, Cambridge, MA, 2010).
13. G. B. Ermentrout, D. H. Terman, *Mathematical foundations of neuroscience* (Springer, New York, 2010).
14. D. Mittal, R. Narayanan, Degeneracy in the robust expression of spectral selectivity, subthreshold oscillations, and intrinsic excitability of entorhinal stellate cells. *J Neurophysiol* **120**, 576-600 (2018).
15. A. L. Hodgkin, A. F. Huxley, A quantitative description of membrane current and its application to conduction and excitation in nerve. *J Physiol* **117**, 500-544 (1952).
16. A. A. Faisal, L. P. J. Selen, D. M. Wolpert, Noise in the nervous system. *Nature Reviews Neuroscience* **9**, 292-303 (2008).
17. R. K. Rathour, R. Narayanan, Homeostasis of functional maps in active dendrites emerges in the absence of individual channelostasis. *Proc Natl Acad Sci U S A* **111**, E1787-1796 (2014).
18. R. C. Cannon, C. O'Donnell, M. F. Nolan, Stochastic ion channel gating in dendritic neurons: Morphology dependence and probabilistic synaptic activation of dendritic spikes. *PLoS Computational Biology* **6** (2010).
19. J. T. Dudman, M. F. Nolan, Stochastically gating ion channels enable patterned spike firing through activity-dependent modulation of spike probability. *PLoS computational biology* **5**, e1000290 (2009).
20. M. M. Shah, M. Migliore, I. Valencia, E. C. Cooper, D. A. Brown, Functional significance of axonal Kv7 channels in hippocampal pyramidal neurons. *Proceedings of the National Academy of Sciences of the United States of America* **105**, 7869-7874 (2008).

21. A. A. Prinz, C. P. Billimoria, E. Marder, Alternative to hand-tuning conductance-based models: construction and analysis of databases of model neurons. *Journal of neurophysiology* **90**, 3998-4015 (2003).
22. E. Marder, A. L. Taylor, Multiple models to capture the variability in biological neurons and networks. *Nat Neurosci* **14**, 133-138 (2011).
23. N. T. Carnevale, M. L. Hines, *The NEURON Book* (Cambridge University Press, Cambridge, 2006), DOI: 10.1017/CBO9780511541612.
24. F. R. Fernandez, J. A. White, Artificial synaptic conductances reduce subthreshold oscillations and periodic firing in stellate cells of the entorhinal cortex. *J Neurosci* **28**, 3790-3803 (2008).
25. E. Fransen, A. A. Alonso, C. T. Dickson, J. Magistretti, M. E. Hasselmo, Ionic mechanisms in the generation of subthreshold oscillations and action potential clustering in entorhinal layer II stellate neurons. *Hippocampus* **14**, 368-384 (2004).
26. H. Pastoll, H. L. Ramsden, M. F. Nolan, Intrinsic electrophysiological properties of entorhinal cortex stellate cells and their contribution to grid cell firing fields. *Frontiers in neural circuits* **6**, 17 (2012).
27. D. Mittal, R. Narayanan, Degeneracy in the robust expression of spectral selectivity, subthreshold oscillations and intrinsic excitability of entorhinal stellate cells. *Journal of neurophysiology* **120**, 576-600 (2018).
28. J. M. Goaillard, E. Marder, Ion Channel Degeneracy, Variability, and Covariation in Neuron and Circuit Resilience. *Annu Rev Neurosci* **44**, 335–357 (2021).
29. R. K. Rathour, R. Narayanan, Degeneracy in hippocampal physiology and plasticity. *Hippocampus* **29**, 980-1022 (2019).
30. H. Pastoll, H. L. Ramsden, M. F. Nolan, Intrinsic electrophysiological properties of entorhinal cortex stellate cells and their contribution to grid cell firing fields. *Frontiers in Neural Circuits* 10.3389/fncir.2012.00017, 1-21 (2012).
31. A. Boehlen, C. Henneberger, U. Heinemann, I. Erchova, Contribution of near-threshold currents to intrinsic oscillatory activity in rat medial entorhinal cortex layer II stellate cells. *Journal of Neurophysiology* **109**, 445-463 (2013).
32. A. D. Dorval Jr, J. A. White, Channel noise is essential for perithreshold oscillations in entorhinal stellate neurons. *Journal of Neuroscience* **25**, 10025-10028 (2005).
33. E. Fransén, A. A. Alonso, C. T. Dickson, J. Magistretti, M. E. Hasselmo, Ionic mechanisms in the generation of subthreshold oscillations and action potential clustering in entorhinal layer II stellate neurons. *Hippocampus* **14**, 368-384 (2004).
34. C. T. Dickson *et al.*, Properties and role of I(h) in the pacing of subthreshold oscillations in entorhinal cortex layer II neurons. *Journal of Neurophysiology* **83**, 2562-2579 (2000).
35. A. Alonso, R. Klink, Differential electroresponsiveness of stellate and pyramidal-like cells of medial entorhinal cortex layer II. *Journal of Neurophysiology* **70**, 128-143 (1993).
36. A. Alonso, R. R. Llinás, Subthreshold Na+-dependent theta-like rhythmicity in stellate cells of entorhinal cortex layer II. *Nature* **342**, 175-177 (1989).
37. R. Klink, A. Alonso, Ionic mechanisms for the subthreshold oscillations and differential electroresponsiveness of medial entorhinal cortex layer II neurons. *Journal of Neurophysiology* **70**, 144-156 (1993).
38. J. A. White, R. Klink, A. Alonso, A. R. Kay, Noise from voltage-gated ion channels may influence neuronal dynamics in the entorhinal cortex. *Journal of Neurophysiology* **80**, 262-269 (1998).
39. H. G. Rotstein, M. Wechselberger, N. Kopell, Canard Induced Mixed-Mode Oscillations in a Medial Entorhinal Cortex Layer II Stellate Cell Model. *Siam J Appl Dyn Syst* **7**, 1582-1611 (2008).
40. H. G. Rotstein, The shaping of intrinsic membrane potential oscillations: positive/negative feedback, ionic resonance/amplification, nonlinearities and time scales. *Journal of Computational Neuroscience* **42**, 133-166 (2017).

41. M. F. Nolan, J. T. Dudman, P. D. Dodson, B. Santoro, HCN1 channels control resting and active integrative properties of stellate cells from layer II of the entorhinal cortex. *Journal of Neuroscience* **27**, 12440-12451 (2007).
42. R. S. G. Jones, Synaptic and intrinsic properties of neurons of origin of the perforant path in layer II of the rat entorhinal cortex in vitro. *Hippocampus* **4**, 335-353 (1994).
43. L. M. Giocomo, M. E. Hasselmo, Time constants of h current in layer II stellate cells differ along the dorsal to ventral axis of medial entorhinal cortex. *Journal of Neuroscience* **28**, 9414-9425 (2008).
44. D. L. F. Garden, P. D. Dodson, C. O'Donnell, M. D. White, M. F. Nolan, Tuning of Synaptic Integration in the Medial Entorhinal Cortex to the Organization of Grid Cell Firing Fields. *Neuron* **60**, 875-889 (2008).
45. I. Erchova, G. Kreck, U. Heinemann, A. V. M. Herz, Dynamics of rat entorhinal cortex layer II and III cells: Characteristics of membrane potential resonance at rest predict oscillation properties near threshold. *Journal of Physiology* **560**, 89-110 (2004).
46. J. S. Haas, A. D. Dorval Ii, J. A. White, Contributions of Ih to feature selectivity in layer II stellate cells of the entorhinal cortex. *Journal of Computational Neuroscience* **22**, 161-171 (2007).
47. P. D. Dodson, H. Pastoll, M. F. Nolan, Dorsal-ventral organization of theta-like activity intrinsic to entorhinal stellate neurons is mediated by differences in stochastic current fluctuations. *Journal of Physiology* **589**, 2993-3008 (2011).
48. J. T. Dudman, M. F. Nolan, Stochastically gating ion channels enable patterned spike firing Through activity-dependent modulation of spike probability. *PLoS Computational Biology* **5** (2009).
49. M. Yoshida, A. Alonso, Cell-type-specific modulation of intrinsic firing properties and subthreshold membrane oscillations by the M(Kv7)-current in neurons of the entorhinal cortex. *Journal of Neurophysiology* **98**, 2779-2794 (2007).
50. F. R. Fernandez, J. A. White, Artificial synaptic conductances reduce subthreshold oscillations and periodic firing in stellate cells of the entorhinal cortex. *Journal of Neuroscience* **28**, 3790-3803 (2008).
51. F. A. Khawaja, A. A. Alonso, C. W. Bourque, Ca2+-dependent K+ currents and spike-frequency adaptation in medial entorhinal cortex layer II stellate cells. *Hippocampus* **17**, 1143-1148 (2007).
